# Genetic Stock Identification Reveals Mismatches Between Management Areas and Population Genetic Structure in a Migratory Pelagic Fish

**DOI:** 10.1111/eva.70030

**Published:** 2024-10-24

**Authors:** Gaute Wilhelmsen Seljestad, María Quintela, Dorte Bekkevold, Christophe Pampoulie, Edward D. Farrell, Cecilie Kvamme, Aril Slotte, Geir Dahle, Anne Grete Sørvik, Mats E. Pettersson, Leif Andersson, Arild Folkvord, Kevin A. Glover, Florian Berg

**Affiliations:** ^1^ Department of Biological Sciences University of Bergen Bergen Norway; ^2^ Institute of Marine Research (IMR) Bergen Norway; ^3^ National Institute of Aquatic Resources Technical University of Denmark Silkeborg Denmark; ^4^ Marine and Freshwater Research Institute Hafnarfjörður Iceland; ^5^ Killybegs Fishermen's Organisation Killybegs Donegal Ireland; ^6^ Science for Life Laboratory, Department of Medical Biochemistry and Microbiology Uppsala University Uppsala Sweden; ^7^ Department of Veterinary Integrative Biosciences, College of Veterinary Medicine and Biomedical Sciences Texas A&M University College Station Texas USA

**Keywords:** Atlantic herring, Baltic Sea, North Sea, Norwegian Sea, population assignment, single nucleotide polymorphism

## Abstract

Sustainable fisheries management is important for the continued harvest of the world's marine resources, especially as they are increasingly challenged by a range of climatic and anthropogenic factors. One of the pillars of sustainable fisheries management is the accurate identification of the biological units, i.e., populations. Here, we developed and implemented a genetic baseline for Atlantic herring harvested in the Norwegian offshore fisheries to investigate the validity of the current management boundaries. This was achieved by genotyping > 15,000 herring from the northern European seas, including samples of all the known populations in the region, with a panel of population‐informative SNPs mined from existing genomic resources. The final genetic baseline consisted of ~1000 herring from 12 genetically distinct populations. We thereafter used the baseline to investigate mixed catches from the North and Norwegian Seas, revealing that each management area consisted of multiple populations, as previously suspected. However, substantial numbers (up to 50% or more within a sample) of herring were found outside of their expected management areas, e.g., North Sea autumn‐spawning herring north of 62° N (average = 19.2%), Norwegian spring‐spawning herring south of 62° N (average = 13.5%), and western Baltic spring‐spawning herring outside their assumed distribution area in the North Sea (average = 20.0%). Based upon these extensive observations, we conclude that the assessment and management areas currently in place for herring in this region need adjustments to reflect the populations present. Furthermore, we suggest that for migratory species, such as herring, a paradigm shift from using static geographic stock boundaries towards spatial dynamic boundaries is needed to meet the requirements of future sustainable management regimes.

## 1 Introduction

In response to the escalating demand for food driven by a growing human population, the efficient and sustainable utilization of biological resources has become crucial. In this context, marine‐derived food is considered one of the most critical sources (Costello et al. 2020). Despite a general agreement on the importance of sea food, overexploitation and detrimental catch practices of marine resources represent a major and increasing challenge (FAO 2022). As a result, sustainability is a topic of rising concern among the public, scientists, and policy makers alike.

In pelagic fisheries, the most common method of harvest is trawling or purse‐seining (Watson, Revenga, and Kura 2006). However, these methods are of relatively low species selectivity, and the catch composition is typically not fully confirmed before it is taken onboard. Fishing vessels are entitled a certain catch quota for a given species, and species quotas by area are determined through negotiations between shareholders based on the input and advice of scientists (Hilborn et al. 2020). Fishing quotas are given for specific stocks (management units), which are often based upon geographical, pragmatic and/or politically determined borders that do not always align with the underlying biological units (i.e., populations; (Cadrin 2020)). Thus, for quota‐based fisheries to become truly sustainable, managed stocks and populations need to be aligned (Kerr et al. 2017). As such, it is essential to identify the biological populations that are present within a defined management area and assess their distribution in time and space.

Over the past decade, genetic studies, such as high‐throughput single nucleotide polymorphisms (SNP) genotyping, enabling the robust identification of population structure, including marine species, have become increasingly accessible (Andersson et al. 2024; Quintela et al. 2020). We can now design panels of genetic markers that can identify populations with a high degree of accuracy. By analyzing samples from known populations, one can make genetic reference population baselines, that can be used for assignment of individuals of unknown origin to their source populations (Anderson, Waples, and Kalinowski 2008). Genetic identification of known populations in mixed fishery can optimize fisheries management even in “real‐time,” e.g., by determining when an area where multiple populations are overlapping should be opened or closed for fishing (Dahle et al. 2018).

The Atlantic herring (*Clupea harengus* L.), hereon called herring, is a small pelagic schooling forage fish present throughout most of the north Atlantic including the Baltic Sea (Whitehead 1985, 1986). It has supported extensive fisheries since the 1100s or earlier, and plays a strong cultural role throughout many parts of Europe (Atmore et al. 2022; Barrett and Orton 2016). Herring are known for their extensive annual migrations (Corten 2002; Slotte and Fiksen 2000), typically, following the migration triangle between feeding grounds, spawning areas, and nursery habitats (Harden Jones 1968). Herring are total spawners depositing their sticky eggs on diverse substrates (Frost and Diele 2022) where different populations show distinct homing behavior (Moll et al. 2019; Ruzzante et al. 2006). Thus, herring populations can be described as sympatric discrete populations (Iles and Sinclair 1982) where “reproductively and genetically isolated populations […] may occupy overlapping habitats, at least during one phase of their lifetime” (Ciannelli et al. 2013) as they mainly mix on their feeding grounds (Berg et al. 2017). Furthermore, as a species, herring spawns throughout the entire year, with spawning times varying markedly between populations (dos Santos Schmidt et al. 2021) which are likely regulated by a combination of genetic and environmental variables (Han et al. 2020). However, switching of spawning seasons is known to occur at low rates, allowing for some potential gene flow to occur (Berg et al. 2021; Kerr et al. 2019).

**FIGURE 1:**
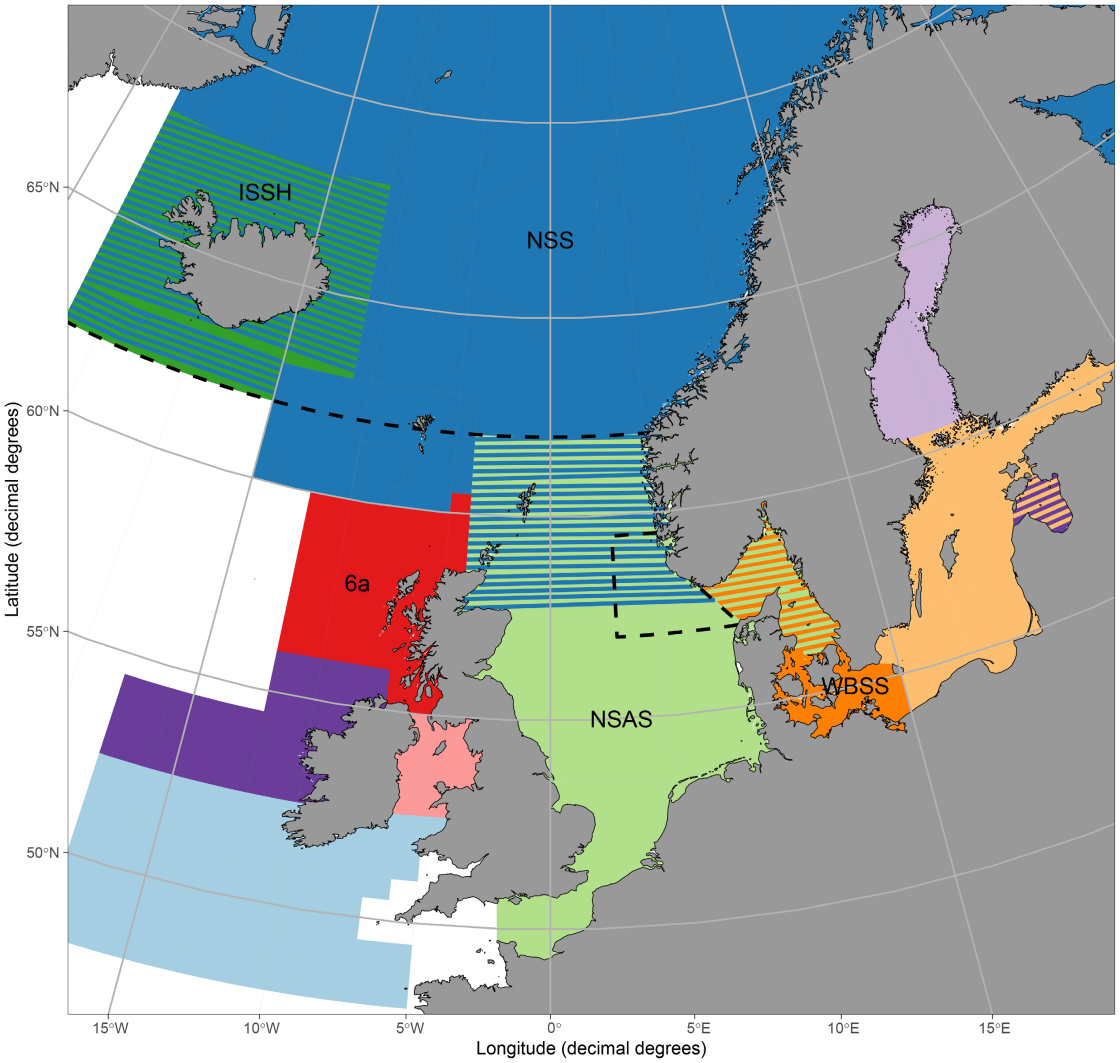
Map of the geographically defined management areas for the 11 stocks (management units in different colors) assessed by the International Council for the Exploration of the Sea (ICES) in the northeastern Atlantic. Stocks occurring in the Norwegian Sea and North Sea (study area) are the *Norwegian spring‐spawning* (*NSS*) herring stock, the *North Sea autumn‐spawning* (*NSAS*) herring stock, the *Icelandic summer‐spawning herring* (*ISSH*) stock, and the *western Baltic spring‐spawning* (*WBSS*) herring stock which are typically named after the putatively most abundant population (biological unit). Herring stocks in ICES subdivision 6a are genetically identified (Farrell et al. 2022). Overlapping management areas are indicated by cross hatched colors. The stippled line indicates the currently used management boundary between the exploited stocks at 62° N. The stippled box in the North Sea indicates the so‐called ‘transfer‐area’ where all herring catches (both from commercial catches and scientific surveys) are being split and allocated either to the *NSAS* or the *WBSS* herring stock for their assessments.

Recent studies have identified hundreds of loci associated with ecological adaptation in herring, such as spawning time, salinity, and temperature (Han et al. 2020; Lamichhaney et al. 2017; Martinez Barrio et al. 2016). Furthermore, four inversions and haplotype divergence among northern and southern herring populations have been identified (Han et al. 2020; Jamsandekar et al. 2023; Pettersson et al. 2019), often reflecting distinct evolutionary trajectories driven by adaptation to local environments (Formenti et al. 2022; Matschiner et al. 2022; Theissinger et al. 2023). Thus, herring does not only consist of multiple sympatric or spatially isolated populations, but also includes highly ecologically adapted ecotypes (Han et al. 2020). This complex population structure has been highlighted by multiple studies (Bekkevold et al. 2023; Farrell et al. 2022; í Kongsstovu et al. 2022; Pampoulie et al. 2024) demonstrating clear genetic differentiation between populations and ecotypes, but also a high degree of sympatry outside their spawning times. However, the genetic population structure along the Norwegian coast, in the Norwegian Sea and North Sea (Figure 1) and their alignment with specific management areas remains unknown.

In the northeastern Atlantic, the International Council for the Exploration of the Sea (ICES) currently gives advice for sustainable harvesting of 11 herring stocks defined by geographical areas (Figure 1). Stocks are typically named after the putatively most abundant population in that specific management area. Consequently, stock names (hereafter in *italic*) can be identical to a population name. Herring has a history of being overfished, and multiple stocks have experienced collapses or been on the brink of collapse (Dickey‐Collas et al. 2010; MacKenzie and Ojaveer 2018; Nichols 2001). Some of these stocks, such as *North Sea Autumn‐Spawning* (*NSAS*) and *Norwegian Spring‐Spawning* (*NSS*) herring, are part of major success stories of fisheries management resulting in stock recoveries due to major changes in their management (Dickey‐Collas et al. 2010; Toresen et al. 2019). Considering this, monitoring the population structure and their dynamics within management areas is important for the future sustainability of the fisheries. One major question is the validity of the current management boundary between *NSS* and *NSAS* stocks, set at 62° N in a rather arbitrary manner. Under the current regime, herring catches north of 62° N, both for surveys and commercial catches, are allocated to the *NSS* herring stock. On the other hand, herring caught south of 62° N and east of 4° W are typically allocated to the *NSAS* herring stock unless a specific stock identification method is used for splitting catches, as, e.g., done in the ‘transfer‐area’ where the *western Baltic spring‐spawning* (*WBSS*) herring stock is identified (Figure 1) (ICES 2023a). A similar geographic management boundary exists at 4° W where the *NSAS* and *6a North autumn‐spawning* herring stocks are separated for assessment and management, despite these two stocks being genetically the same population (Figure 1) (Farrell et al. 2022; ICES 2024). Additionally, other winter‐ and spring‐spawning populations in ICES subdivision 6a are now genetically separated from the *6a North autumn‐spawning* herring stock for assessment and management. Furthermore, both *NSS* and *NSAS* stocks are comprised of several populations and even mix with other stocks during their annual migrations (Berg et al. 2017).

Given the large economic and ecological importance of the highly abundant herring stocks in the north Atlantic, a detailed understanding of population genetic structure and mixing of these populations within and between management areas is needed. For example, to what degree are different populations present within a managed stock area, and to what degree does mixing occur between the management areas of *NSS* and *NSAS*. In order to address these and similar important issues, the present study was designed to:

1. Characterize the population genetic structure of herring in the Norwegian Sea and North Sea using samples from already identified populations and from putatively new populations.
2. Utilize the identified population structure as a genetic baseline to assign individual herring of mixed‐population catches from scientific surveys and commercial landings to their population of origin.
3. Investigate if the spatial distribution of populations aligns with the current management areas.

We addressed the above objectives, by genotyping > 15,000 herring from known populations and survey catches using a panel of 60 discriminatory SNPs selected from an extensive genomic data resource established by whole genome sequencing (Han et al. 2020).

## 2 Materials and Methods

### 2.1 Samples

**FIGURE 2:**
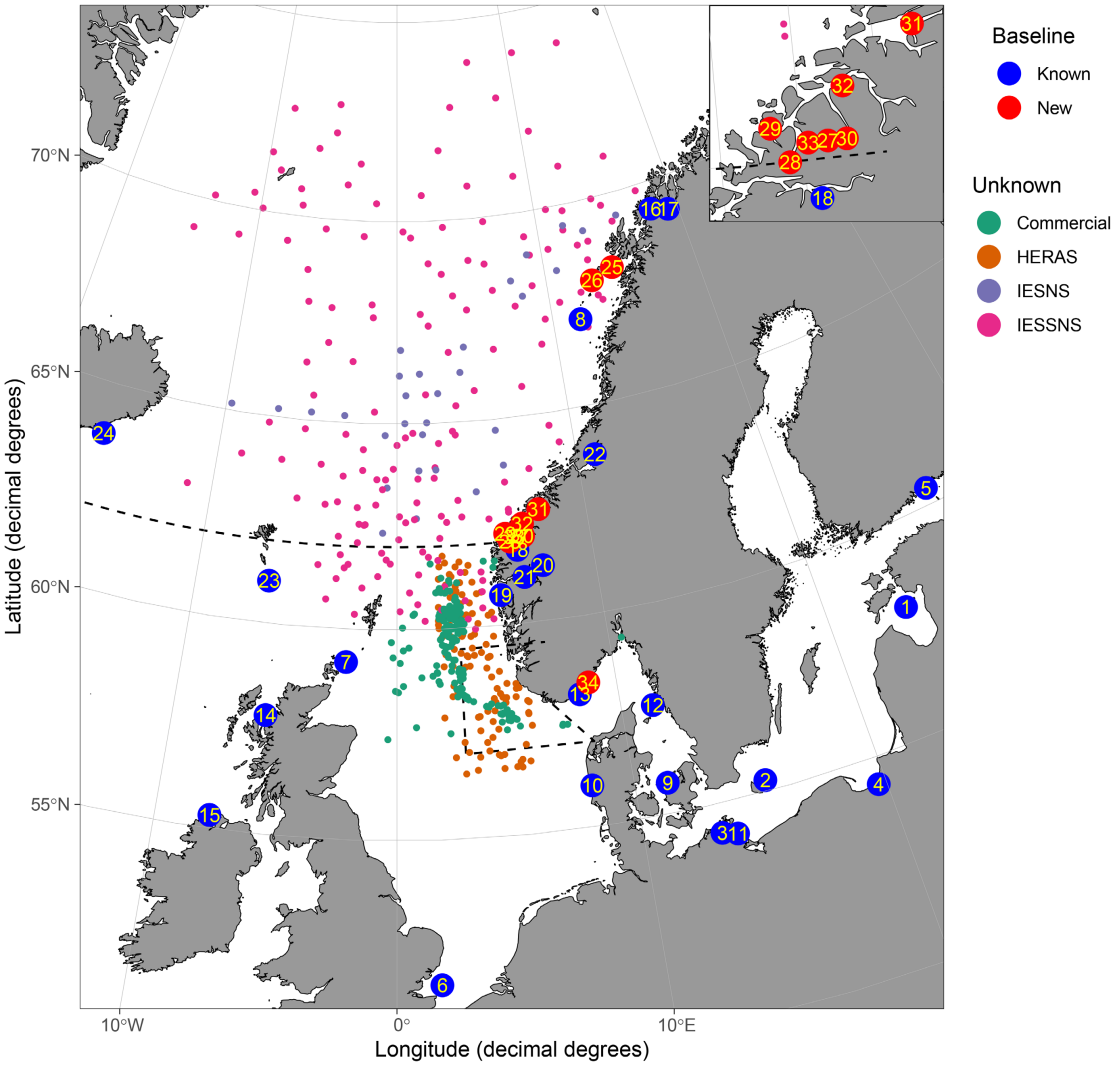
Map of sampling locations. Large points show baseline samples (see Table 1 for detailed information), and smaller points show mixed‐population samples from commercial catches and scientific surveys (HERAS, HERring Acoustic Survey; IESNS, International Ecosystem Survey in the Nordic Seas; IESSNS, International Ecosystem Summer Survey in the Nordic Seas) in 2019–2023. Inset, top right corner: Detailed map of the Sunnmøre area. The stippled lines are as in Figure 1.

This study covered almost the entire distribution area of herring in the greater North Sea ecoregion, Baltic Sea, Icelandic waters, Norwegian Sea as well as the Norwegian coast and its fjords (Figure 2). Three different types of samples were collected and/or (re)analyzed: (1) known reference baseline samples, i.e., individuals genetically identified as distinct spawning unit (Bekkevold et al. 2023; Farrell et al. 2022), (2) potentially undescribed populations that need to be include as baseline samples, and (3) mixed‐population samples from scientific surveys and commercial catches to be identified against the updated genetic baseline (Tables 1 and 2).

**TABLE 1:** Baseline samples of known (^1^Bekkevold et al. (2023); ^2^Farrell et al. (2022); ^3^this study) and putative new populations: Sampling sites according to geographically explicit locations for each putative baseline sample, year of capture, and total initially genotyped number of individuals that meet the threshold of acceptance of missing data (*N*). Genetically distinct populations included in the final baseline and number of individuals per population (N_Pop) after balancing the dataset and removal of outliers. Sampling sites displaying very little/no differentiation were merged into one population.

No.	Sampling site	Abbr.	Lat	Long	Year	*N*	Baseline	Population	N_Pop
1	Gulf of Riga	BASH_GR	58.02	23.50	2014	46	Known^1,3^	BASH	102
2	Bornholm Basin^a^	BASH_CB	55.26	15.33	2016	38	Known^1^		
3	Western Baltic Sea (Rügen)^a^	BASH_RU	54.21	13.42	2019	18	Known^1^		
4	Vistula Lagoon (Gdańsk)	CBSS_GD	54.37	19.67	2009	70	Known^1,3^	CBSS	82
5	Gulf of Finland	CBSS_GF	60.40	26.70	2009	41	Known^1^		
6	Southern Bight	Downs	51.63	1.68	2016	104	Known^1,3^	Downs	104
7	North Sea	NSAS	59.21	−2.38	2011	60	Known^1,3^	NSAS	60
8	Norwegian Sea	NSS	67.12	11.74	2021	140	Known^3^	NSS	140
9	Kattegat	WBSS_KA	55.73	11.37	2009	40	Known^1^	WBSS	122
10	Ringkøbing Fjord	WBSS_RF	55.97	8.24	2009	38	Known^1^		
11	Western Baltic Sea (Rügen)	WBSS_RU	54.21	13.62	2009	45	Known^1^		
12	Skagerrak East (Öckerö)	WBSS_SKE	57.60	11.40	2009	42	Known^1^	WBSS‐SK	63
13	Skagerrak West (Høvåg)	WBSS_SKW	58.15	8.27	2015	24	Known^3^		
14	The Minch, West of Scotland^b^	Sp‐6aN	57.81	−5.87	2018	29	Known^2^	Sp‐6a	58
15	Donegal^b^	Sp‐6aS	55.31	−7.75	2022	29	Known^3^		
16	Rossfjordvannet	ROSSFJ	69.32	18.27	2015	18	Known^3^	Pacific‐Hybrids	30
17	Balsfjorden	BALSFJ	69.18	19.22	2015	12	Known^3^		
18	Gloppenfjorden	GLOPFJ	61.78	6.17	2013	12	Known^3^	Local‐Fjords	108
19	Lindåspollen	Lindas	60.73	5.13	2010	35	Known^3^		
20	Lustrafjorden	LUFJ	61.34	7.37	2011, 2021	82	Known^3^		
21	Sognefjorden	SOGNEFJ	61.11	6.38	2015	28	Known^3^		
22	Trondheimsfjorden	THF	63.80	11.00	2020	205	Known^3^	THF	100
23	North Atlantic (Faroe Islands)	FASH	61.02	−6.38	2009	34	Known^1^	NASS	129
24	North Atlantic (Iceland)	ISSH	63.75	−16.39	2009, 2010	476	Known^3^		
25	North Atlantic (Lofoten)	NASH	68.20	14.50	2019	97	New		
26	Kirkefjorden	KIRKFJ	68.00	13.00	2021	94	New	^c^	
27	Austefjorden	AUFJ	62.10	6.31	2020	119	New	^c^	
28	Dalsfjorden	DAFJ	62.00	5.83	2020	76	New	^c^	
29	Gursken	GURS	62.20	5.63	2021	68	New	^c^	
30	Hjørundfjorden	HJFJ	62.10	6.54	2020	111	New	^c^	
31	Romsdalsfjorden	ROMSFJ	62.70	7.50	2019	32	New	^c^	
32	Sykkylven	SYKK	62.40	6.56	2020, 2022	136	New	^c^	
33	Volda	VOL	62.10	6.07	2020	39	New	^c^	
34	NSS‐Strandfjorden	NSS‐SF	58.30	8.53	2015	30	New	^c^	
	Total					2468			1098

^a^ Larvae samples.

^b^ Muscle tissue samples.

^c^ Samples were redundant and therefore removed from the final baseline.

**TABLE 2:** Total number of herring from mixed‐population samples collected and analyzed during scientific surveys (HERAS, HERring Acoustic Survey; IESNS, International Ecosystem Survey in the Nordic Seas; IESSNS, International Ecosystem Summer Survey in the Nordic Seas) and from commercial catches between 2019 and 2023.

Ecoregion	Source	Year	N
North Sea	HERAS	2019	672
North Sea	HERAS	2020	825
North Sea	HERAS	2021	785
North Sea	HERAS	2022	1188
North Sea	HERAS	2023	1247
North Sea	Commercial	2021	1232
North Sea	Commercial	2022	1947
North Sea	Commercial	2023	3453
Norwegian Sea	IESNS	2020	188
Norwegian Sea	IESNS	2021	273
Norwegian Sea	IESNS	2022	235
Norwegian Sea	IESSNS	2019	286
Norwegian Sea	IESSNS	2020	599
Norwegian Sea	IESSNS	2021	1174
Norwegian Sea	IESSNS	2022	1566
Total			15,670

The reference baseline was built either from fin clips (Samples 1, 4–13, 16–24) or muscle tissue (Samples 14–15, *N* = 58) of ripe and spawning individuals (stage 5–6) or newly hatched larvae (Samples 2–3, *N* = 56) collected on known spawning grounds for previously identified genetically distinct populations (Table 1). The maturity of herring was determined by visual inspection of gonads according to the following scale: immature = 1–2, maturing = 3–4, ripe = 5, spawning = 6, spent = 7 and recovering = 8 (Mjanger et al. 2019). Most of these samples have been described in detail in Farrell et al. (2022) and Bekkevold et al. (2023) as well as summarized in the Supporting Information. Additional reference baseline samples were collected from Norwegian spring spawners (NSS), Icelandic summer spawners (ISSH), spring spawners from the Norwegian Skagerrak coast (WBSS_SKW), as well as samples from multiple fjords along the Norwegian west coast. Samples from previously described fjord populations were collected from Balsfjorden (Jørstad and Pedersen 1986; Mikkelsen et al. 2018), Rossfjordvatnet (Hognestad 1994; Mikkelsen et al. 2018), Gloppenfjorden (Libungan et al. 2015), Lindåspollen (Johannessen et al. 2009; Lie, Dahl, and Ostvedt 1978), Lustrafjorden (Aasen 1952), Sognefjorden (dos Santos Schmidt et al. 2021), and Trondheimsfjorden (Runnström 1941). Herring from Balsfjorden and Rossfjordvannet are known to be hybrids between Atlantic and Pacific herring (*C. pallasii*) (Mikkelsen et al. 2018; Pettersson et al. 2023).

Potentially undescribed baseline samples were collected on local spawning grounds along the Norwegian coast during both spring‐ and autumn‐spawning seasons (Table 1: Samples 25–33). Based on their maturity stage, spring‐ and autumn‐spawning herring were identified co‐occurring during both spawning seasons on these spawning grounds. Thus, these samples do not represent “true” baselines sample consisting of spawning individuals only. To avoid a mixture of autumn‐ and spring‐spawning herring, and since both are often genetically distinct (Bekkevold et al. 2023; Lamichhaney et al. 2017), individuals were first genetically identified (Table 3, see the Supporting Information for details). Further analyses (detailed below) were needed to identify if herring from these samples represented either new distinct populations or populations already in the reference baseline. Potential baseline samples were included in the final baseline when herring were genetically identified as new distinct populations. Otherwise, they were discarded because they did not represent “true” baseline samples due to the mixing of different spawning types. These samples were collected from several fjords in Sunnmøre (Norway) near the main spawning grounds of NSS herring (Austefjorden, Dalsfjorden, Gursken, Hjørundfjorden, Romsdalsfjorden, Sykkylven, and Volda), near the Lofoten Islands (Kirkefjorden and inner Vestfjorden [Sample 25]), and along the Norwegian Skagerrak coast (Sample 34, assigned as NSS based on their otolith shape) (Eggers et al. 2014) (Figure 2; Table 1).

**TABLE 3:** Potentially new baseline: Distribution of individuals identified as genetic autumn spawners (_gas) and genetic spring spawners (_gss) after running STRUCTURE at *K* = 2. A threshold of ancestry to cluster of *q* ≥ 0.70 was used and the individuals not meeting this requirement were left unassigned (na) and thus removed from subsequent analyses.

Sample	Autumn spawners (_gas)	Spring spawners (_gss)	na
North Atlantic (Lofoten)	46	45	6
Kirkefjorden	90	3	1
Austefjorden	48	61	10
Dalsfjorden	28	46	2
Gursken	25	27	16
Hjørundfjorden	2	109	0
Romsdalsfjorden	25	7	0
Sykkylven	6	124	6
Volda	6	30	3
NSS‐Strandfjorden	30	0	0
Total	306	452	44

Mixed‐population samples were collected during three international scientific surveys in the North Sea and Norwegian Sea with potentially high mixing aggregations of herring populations (Table 2). Samples were collected on the Norwegian parts of the HERring Acoustic Survey (HERAS) in June–July 2019–2023, the “International Ecosystem Survey in the Nordic Seas” (IESNS) in May 2020–2022, and the “International Ecosystem Summer Survey in the Nordic Seas” (IESSNS) in July 2019–2022. Furthermore, samples from commercial catches between 2021 and 2023 from the North Sea were included. Random samples of 30–50 individuals per catch were collected from both survey and commercial samples. All HERAS samples collected were genotyped and analyzed for the current study. For other surveys and commercial catches, a subset of samples, selected based on the probability of mixing, were genotyped and any abundance estimates would be biased due to this selection. The mixed‐population samples were genetically assigned using the final baseline presented in this study.

### 2.2 SNP Panel and Molecular Analyses

DNA was extracted from all herring (*N* = 18,138) in 96‐well plates using the Qiagen DNeasy 96 Blood & Tissue Kit or by Beckman Coulter DNAdvance–Genomic DNA Isolation Kit on a Biomek i5 Automated Workstation following manufacturer's instructions (Beckman Coulter 2021; Qiagen 2016).

A panel of SNPs distinguishing between populations assumed to occur in the Norwegian Sea and North Sea was established from mining genomic data from the herring genome (Han et al. 2020; Pettersson et al. 2019) following a similar set of criteria as described in Bekkevold et al. (2023). It should be noted that the 6a spring‐spawning herring (Sp‐6a) samples were added after the SNP panel was designed and as such the panel may not be optimized to discriminate this population from other populations. Otherwise, all baseline populations were considered during the SNP panel development. In short, SNPs were selected based on sequence data (Han et al. 2020) and their inferences about genomic regions influenced by selective sweeps, as well as association with characteristics such as spawning time, salinity, and geography. Additional markers identified by Han et al. (2020) to discriminate populations along the Norwegian coast and inside the fjords were also included. The final panel consisted of 60 SNPs (Table S1) covering 20 chromosomes all associated with selective outlier regions from Han et al. (2020). In contrast to Bekkevold et al. (2023), which focused on establishing a SNP panel for population differentiation primarily in the North Sea‐Baltic Sea transition area, the SNPs chosen here were selected to primarily differentiate populations likely present in the North Sea, Norwegian Sea and along the Norwegian coast. Therefore, the number of SNPs to discriminate among populations within the Baltic Sea, e.g., southern vs. northern central Baltic herring or WBSS from inner Danish water vs. WBSS from Rügen (Bekkevold et al. 2023), was reduced in the present panel to only identify the overall groups. One out of the 60 SNPs displayed linkage with the sex determination gene (Rafati et al. 2020), i.e., it was not informative on population/stock level, and was thus not included in the following baseline analysis. For the final panel, primers and unextended primers (UEP) were organized originally in four multiplexes but later in three multiplexes using the Assay Design software (Agena Bioscience) for high throughput genotyping on an Agena MassARRAY iPLEX Platform (Agena Bioscience n.d.). We investigated how many individuals from the baseline samples would be removed by choosing three levels of acceptance of missing data (5.2%, 9.2% and 25%). Finally, a compromise threshold was decided at 25% to prevent depleting samples with few individuals. Along all the process of baseline building, efforts were made to balance the number of individuals per sample to avoid overrepresentation of populations. However, it is worth mentioning that in the final baseline, consisting of genotypes from 1098 individuals, only 9 of the individuals (i.e., < 1%) reached the maximum of 25% missing data whereas 416 individuals (38%) displayed ≤ 5.2% missing data.

### 2.3 Statistical Genetic Analyses

Genetic structure among the baseline samples was assessed using different approaches. A principal component analysis (PCA) was conducted using the function *dudi.pca* in the R‐package (R Core Team 2022) *ade4* (Dray and Dufour 2007) after replacing missing data with the mean allele frequencies, using unscaled allele frequencies (scale = FALSE). Pairwise *F*_ST_ (Weir and Cockerham 1984) was computed with Arlequin v.3.5.1.2 (Excoffier, Laval, and Schneider 2005) with significance assessed after 10,000 permutations. Likewise, the relationships among samples were also examined using the Discriminant Analysis of Principal Components (DAPC; Jombart, Devillard, and Balloux 2010) implemented in *adegenet* (Jombart 2008). To avoid overfitting, both the optimal number of principal components and discriminant functions to be retained were determined using the cross‐validation function (Jombart and Collins 2015; Miller, Cullingham, and Peery 2020). In addition, the Bayesian clustering approach implemented in STRUCTURE v.2.3.4 (Pritchard, Stephens, and Donnelly 2000) and conducted with *ParallelStructure* (Besnier and Glover 2013), was used to identify genetic groups under a model assuming admixture and correlated allele frequencies without using population information. Ten runs per group (*N* = 2–15) were used with a burn‐in period consisting of 100,000 replications and a run length of 1,000,000 Markov‐chain Monte Carlo (MCMC) iterations.

For the set of samples integrating the final baseline, observed (*H*_o_) and unbiased expected heterozygosity (*uH*_e_) as well as inbreeding coefficient (*F*_IS_) were computed with GenAlEx v6.1 (Peakall and Smouse 2006). Since some populations had quite low number of individuals, we estimated *uH*_e_ to assess the genetic variation within a population (Nei and Roychoudhury 1974). The False Discovery Rate (FDR) correction of Benjamini and Hochberg (1995) was applied to *p*‐values to control for Type I errors.

### 2.4 Baseline Construction

A two‐step approach was used to construct the baseline for assigning mixed‐population samples. First all known and previously described reference baseline samples were included. Since the number of SNPs discriminating among Baltic Sea samples were reduced, discrimination power of the SNP panel to distinguish all Baltic Sea herring populations from other reference samples was tested. Second, the baseline was extended to include previously undescribed baseline samples appearing as genetically distinct populations after data mining. The establishment of the final baseline included several rounds of testing and merging of samples, steps that are extensively described in the Supporting Information.

### 2.5 Testing Baseline Accuracy and the Assignment of Mixed‐Population Samples

The accuracy of the SNP panel to assign unknown herring to populations identified in the established baseline was estimated independently by the R‐packages *rubias* (Moran and Anderson 2019) and *assignPOP* (Chen et al. 2018). While *rubias* is applying Bayesian inference from the conditional genetic stock identification model (Moran and Anderson 2019) for the assignment of unknown individuals, machine learning functions such as support vector machines (SVMs) are used in *assignPOP* (Chen et al. 2018). Thus, *assignPOP* is not solely restricted to the use of genetic data and other non‐genetic data can be used for classifications.

Using *rubias*, the individual assignment accuracy of the baseline was tested by leave‐one‐out (LOO) analysis as well as cross‐validation simulations. For the LOO analysis, self‐assignments of all baseline individuals were calculated independently. The proportion of individuals assigned to the population from which they were collected was used as an estimator of baseline accuracy. For the cross‐validation simulations, a MCMC cross‐validation was performed to produce 500 simulated mixtures of 100 individuals (multi‐locus genotypes), drawn randomly without replacement among baseline samples, and assigning them to a population. In contrast to the LOO analysis, this method splits the baseline data into different subsets for each simulation using the Dirichlet parameter for simulating the proportions of reporting units (Moran and Anderson 2019). One subset is used as “new baseline” and the others as mixture samples to estimate the self‐assignment accuracy of the baseline. To evaluate the baseline performance among populations the mean squared error (MSE) was calculated between estimated and simulated proportions. For assignment of mixed‐population samples, individual posterior assignment probabilities were estimated by a fully Bayesian model conditional on the reference allele frequencies of the baseline populations with a parametric bootstrapping correction (2000 MCMC iterations, 100 burn‐in, 100 bootstraps). Bootstrap corrected posterior means of membership in each grouping were used to assign individuals to their most likely baseline population.

Using *assignPOP*, the baseline accuracy was tested with both the Monte‐Carlo (proportions of training dataset = 50%, 70%, and 90%) and *K*‐fold cross‐validation (*K* = 3, 4, 5, and 15) using the default settings. Both methods split the baseline data into a training and a test dataset where the test was used to estimate predictive accuracy of the training dataset. For the Monte‐Carlo cross‐validation a fixed proportion (50%, 70%, and 90%) of each baseline population was kept in the training set, and 100 iterations were estimated. With this random selection of individuals, it is not guaranteed that all individuals will be part of the test dataset. Therefore, the *K*‐fold cross‐validation was performed by splitting the data into *K* groups (in our case 3, 4, 5, and 15 groups). One group was used as the test dataset and the remaining groups as the training dataset. Then *K* assignment tests were performed until every group was tested. In this way, it was guaranteed that each individual was tested once. Furthermore, different classification models such as linear discriminant analysis (LDA), random forest, SVM, and naïve Bayes were tested for the cross‐validation. SVM yielded highest self‐assignment accuracy results for the baseline and was therefore used for assignment of mixed‐population samples, performing a one‐time final assignment test for each unknown individual. During the assignment test, the predicted baseline population and its posterior probability of membership to each population of the baseline are estimated for every individual from the mixed‐population samples. All assignments of mixed‐population samples were conducted against the full baseline.

## 3 Results

### 3.1 Baseline Construction

**FIGURE 3:**
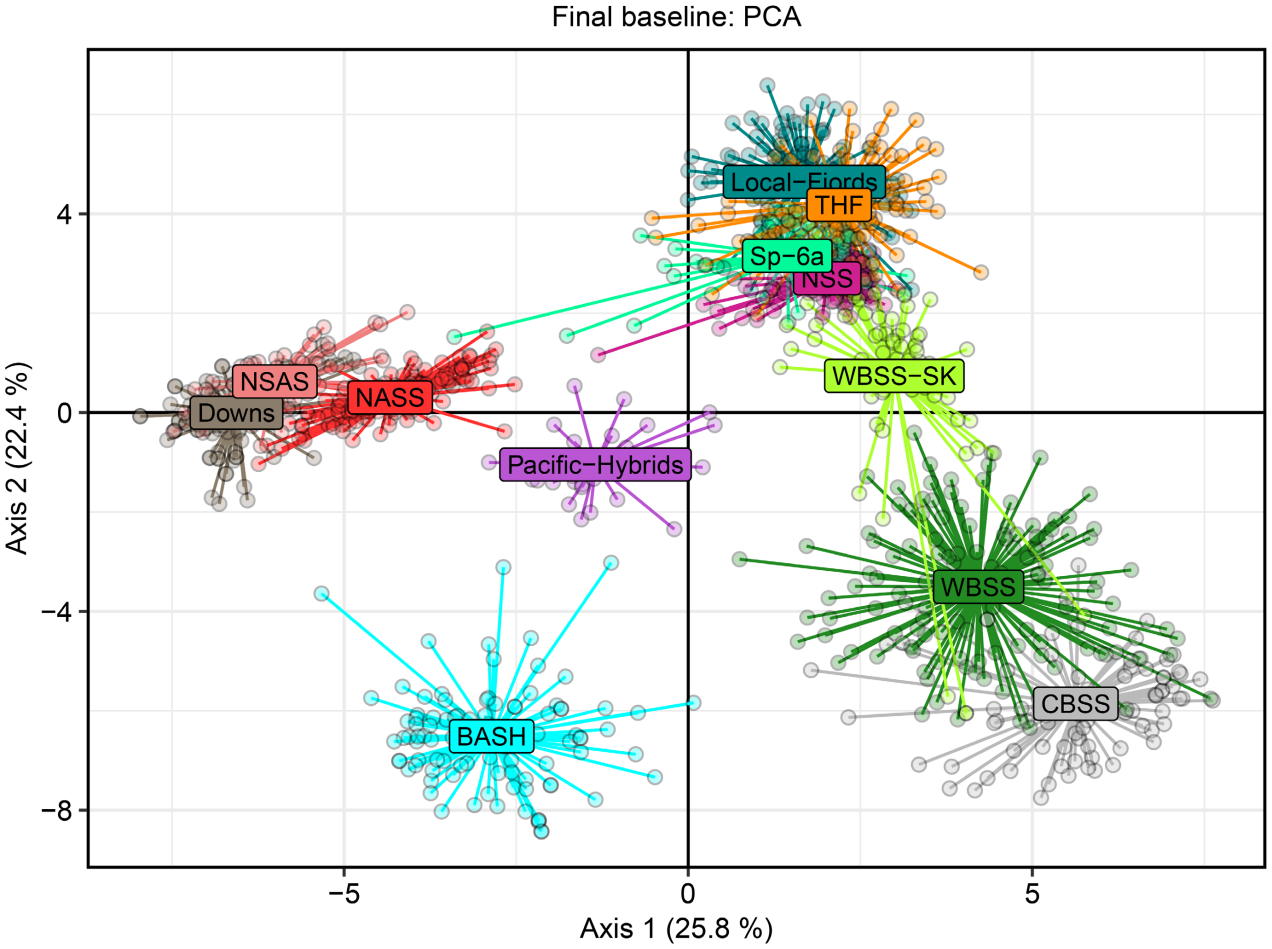
Final baseline: Principal component analysis (PCA) biplot of the final baseline including 12 distinct herring populations: Baltic autumn‐spawning herring (BASH), central Baltic spring‐spawning herring (CBSS), Downs, local fjord populations, northeast Atlantic summer‐/autumn‐spawning herring (NASS), North Sea autumn‐spawning herring (NSAS), Norwegian spring‐spawning herring (NSS), spring‐spawning herring in ICES subdivision 6a (Sp‐6a), Trondheimsfjord herring (THF), western Baltic spring‐spawning herring (WBSS), western Baltic spring‐spawning herring in the Skagerrak (WBSS‐SK), and Pacific herring hybrids. Spawning time (autumn/winter negative, spring positive) represents the major driver of the differentiation displayed in the first axis. While the second axis seems to represent geographical/environmental distribution, and likely also multiple defining features e.g., differences in salinity.

The final baseline consisted of 1098 individuals distributed across 12 genetic groups regarded as genetically distinct populations (Table 1). The nine SNPs associated with spawning time represented the major driver of differentiation, as displayed by the first axis of the PCA biplot (Figure 3), whereas the second axis primarily reflected the differentiation among samples according to geography/ecosystems. Axes 1 and 2 (Figure S8a,b) of the Discriminant Analysis of Principal Components (DAPC) agreed with the PCA pattern whereas the third axis differentiated the Pacific‐hybrids (Figure S8b,c). However, when discarding the Pacific‐hybrids from the baseline, the second axis of the DAPC singled the BASH sample out from the remaining ones (Figure S10).

All 12 putative populations in the baseline were significantly differentiated from each other, with pairwise *F*_ST_ values across all markers ranging from 0.12 to 0.86 (Table S6). These high *F*_ST_ values occur because the SNPs had been selected on the basis of displaying show strong differentiation between populations of herring (Han et al. 2020). The lowest genetic differentiation was found between CBSS and WBSS (*F*_ST_ = 0.12). In general, the Pacific hybrids had high levels of differentiation towards all other populations. Excluding the Pacific‐hybrids, the largest level of differentiation was found between NSS and Downs (*F*_ST_ = 0.83; Table S6). Based on the targeted panel of SNPs, autumn spawners showed lower levels of genetic diversity than spring spawners in terms of percentage of polymorphic loci, as well as observed and expected heterozygosity without any measure reaching statistical significance (Kruskal‐Wallis *p* > 0.05 in all cases; Table S7).

### 3.2 Accuracy of the Baseline for Individual Assignment

All cross‐validation methods demonstrated that the genetic baseline developed here had a high accuracy of assigning individual herring to their population of origin. The highest self‐assignment accuracy of 93% was reached using the leave‐one‐out cross‐validation of *rubias* (Table 4), but only slightly higher than the Monte‐Carlo or *K*‐Fold cross‐validation of *assignPOP* using the SVM classification model (89%, Tables S8 and S9; Figure S11). In the following analysis, we focused on *rubias* results only, but present the *assignPOP* results in the Supporting Information for comparison (Tables S8–S11; Figures S11–S13). The mean square errors (MSEs) of the simulation study were overall low (Table 4). The spring‐spawning herring from ICES subdivision 6a (Sp‐6a) had the lowest self‐assignment rates, mostly misassigned as NSS (Table 4). In general, mis‐assigned individuals mostly were assigned to geographically neighboring populations or to the same spawning season (i.e., spring vs. autumn).

**TABLE 4:** Cross‐validation of the genetic baseline using the on leave‐one‐out in *rubias*. Shaded files show the assignments to their population of origin. Mean square error (MSE) of self‐assignment rates was calculated. Total assignment accuracy of the baseline is 93.68%.

True pop	MSE	Predicted population											
		BASH	CBSS	Downs	Local‐Fjords	NASS	NSAS	NSS	Pacific‐Hybrids	Sp‐6a	THF	WBSS	WBSS‐SK
BASH	9.02E‐06	100	0	0	0	0	0	0	0	0	0	0	0
CBSS	1.09E‐04	0	96.3	0	0	0	0	0	0	0	0	3.7	0
Downs	4.05E‐05	0	0	97.1	0	0	2.9	0	0	0	0	0	0
Local‐Fjords	1.87E‐04	0	0	0	88.9	0	0	4.6	0	0.9	5.6	0	0
NASS	7.06E‐05	0	0	0	0	94.6	5.4	0	0	0	0	0	0
NSAS	1.20E‐04	0	0	3.3	0	1.7	95	0	0	0	0	0	0
NSS	4.93E‐04	0	0	0	0	0.7	0	97.1	0	2.2	0	0	0
Pacific‐Hybrids	8.86E‐06	0	0	0	0	0	0	0	100	0	0	0	0
Sp‐6a	3.04E‐04	0	0	0	0	0	1.7	15.5	0	82.8	0	0	0
THF	1.15E‐04	0	0	0	4	0	0	2	0	1	93	0	0
WBSS	2.27E‐04	0	7.4	0	0	0	0	0	0	0	0	89.3	3.3
WBSS‐SK	2.40E‐04	0	0	0	1.6	0	0	3.2	0	0	0	9.5	85.7

### 3.3 Assignments of the Mixed‐Population Samples

**FIGURE 4:**
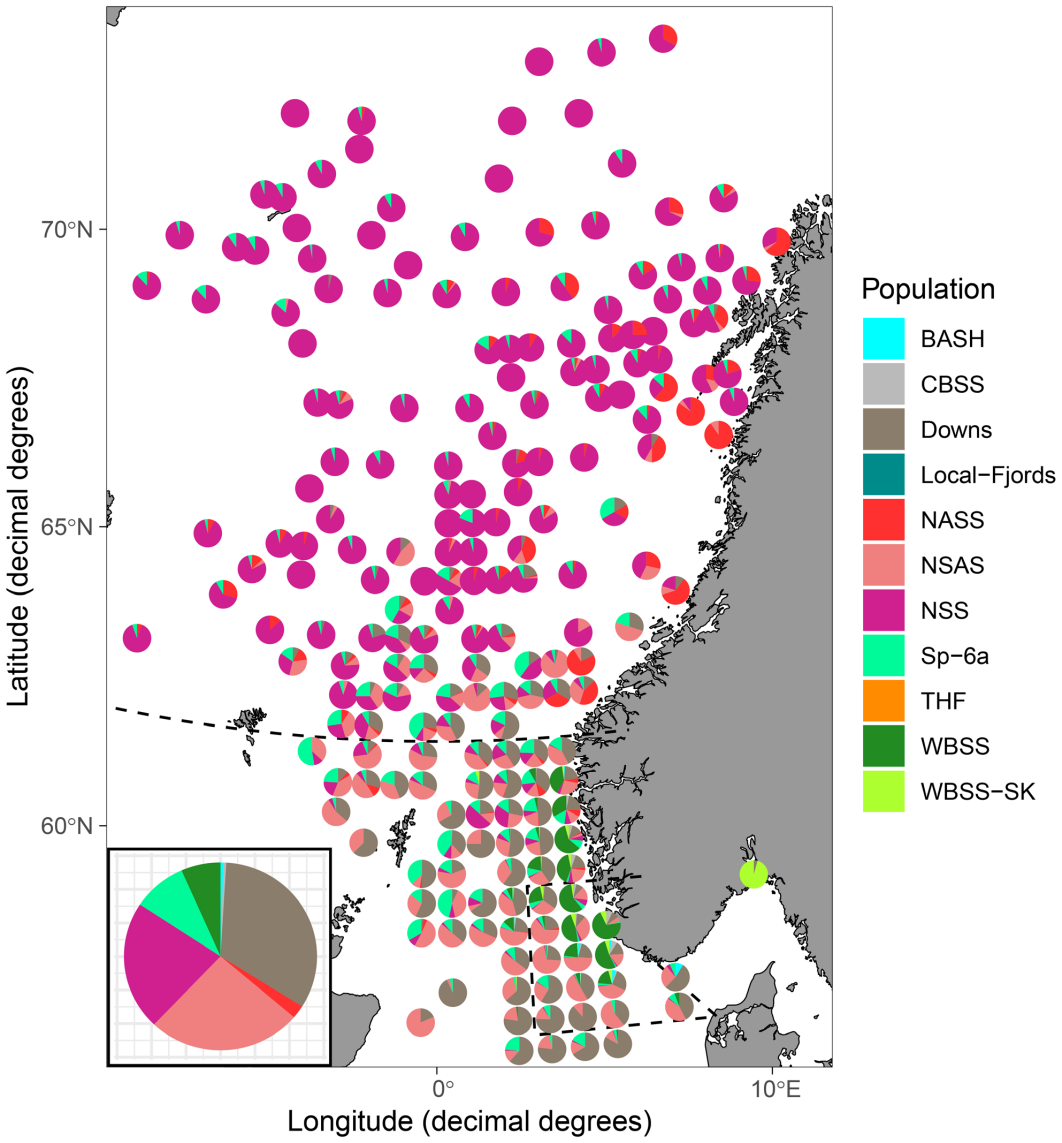
Assignment results based on *rubias* for all mixed‐population samples combined for all years and ICES rectangles (1° longitude × 0.5° latitude). Note that the size of the pie charts does not reflect neither catch size nor sample size. Pie chart in the left corner represents the overall distribution of all samples combined, actual proportions of stocks per samples are provided in Figure 5. Population abbreviations are explained in the legend of Figure 3.

**FIGURE 5:**
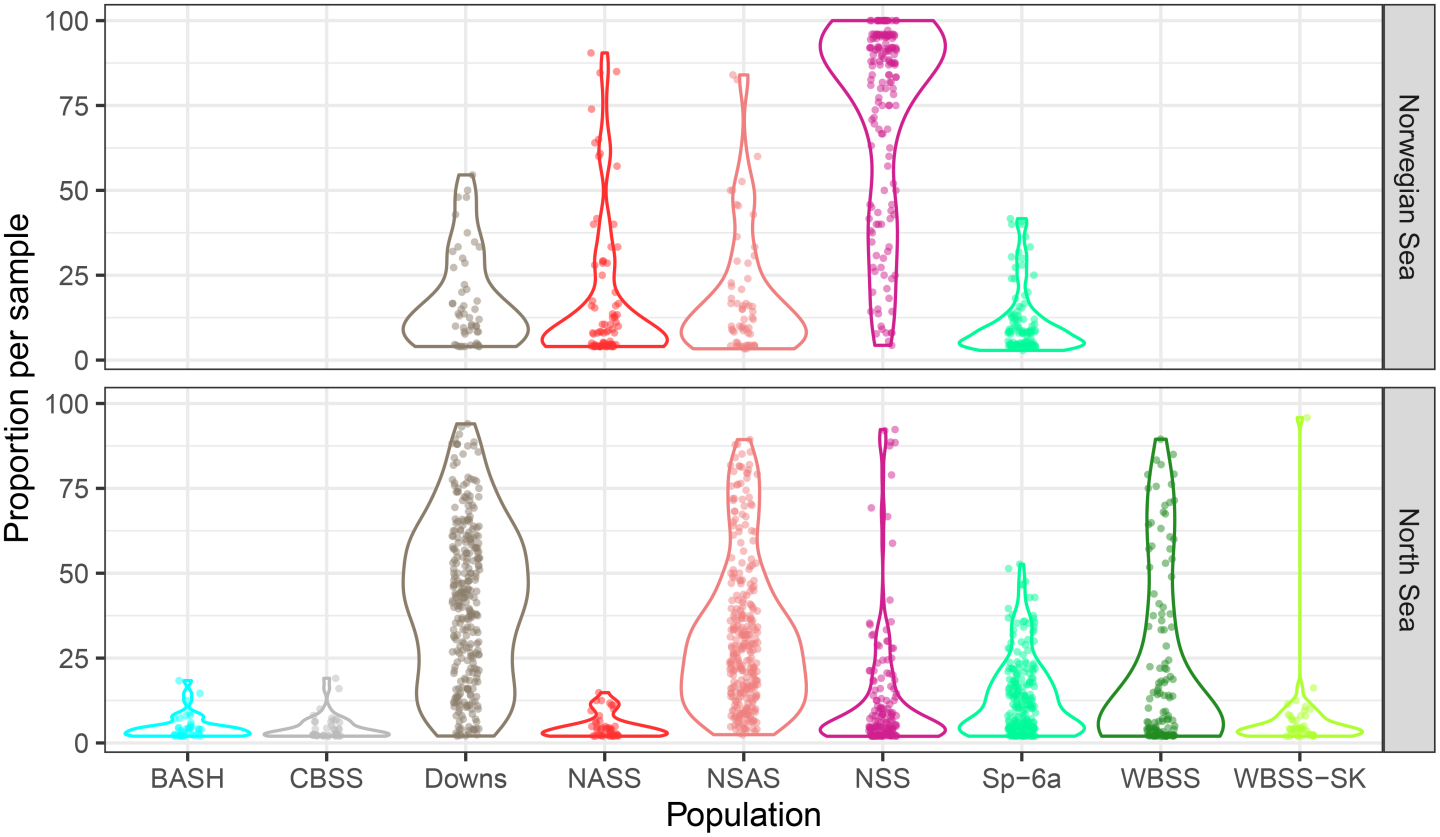
Violin plot showing the distribution of population proportions for all individual mixed‐population samples (*N* = 457, indicated by points) in the Norwegian Sea (north of 62° N) and the North Sea (south of 62° N). Assignment results based on *rubias* for all mixed‐population samples. Note that the absence (null observation) per populations is not included. Population abbreviations are explained in the legend of Figure 3.

In total, 15,132 herring from mixed‐population samples captured during commercial fisheries and scientific surveys between 2019 and 2023 were assigned to the baseline samples with an average posterior probability of 97.8% and 74.0% for *rubias* and *assignPOP*, respectively. The two different assignment methods in general had a high overall agreement (81.0%; Table S10). It should be noted that the posterior assignment probability slightly decreased (slope = −0.002) when the number of missing SNPs increased. The assignments of mixed‐population samples demonstrated that populations were identified outside their geographically defined management areas, such as NSAS or Downs herring found north of 62° N in the Norwegian Sea, WBSS outside the ‘transfer‐area’ in the North Sea, and Sp‐6a in the North Sea and Norwegian Sea (Figure 4; Table S11). The proportions of Sp‐6a herring in the eastern part of the North Sea were significant, constituting up to 50% of any given sample and 12.3% on average (Figure 5). Similarly, catches in the Norwegian Sea and in the North Sea contained up to 84% NSAS (average = 19.2%) and up to 92% NSS (average = 13.5%) herring, respectively (Figure 5). Populations associated with the Baltic Sea, such as BASH, CBSS, and WBSS, were identified repeatedly far north of the ‘transfer‐area’ and thus outside their management area, but not north of 62° N (Figure 5; Table S11).

## 4 Discussion

In the present study, we established a genetic baseline for Atlantic herring and used it to investigate whether the current management boundaries for this species in the North and Norwegian Seas align with the distributions of the populations present in these areas. After analysis of > 15,000 herring sampled in the period 2019–2023, we identified several significant misalignments between the current management areas and the distribution of genetically distinct populations within them. Specifically, none of the existing geographic management boundaries, e.g., at 62° N, at 4° W or the so‐called “transfer‐area”, are valid because multiple herring populations migrate beyond these boundaries in substantial numbers during their annual migrations. We therefore conclude that the current static stock definitions for Atlantic herring in this region do not align with the dynamic distribution of the underlying genetically distinct populations. Furthermore, we suggest that for migratory species, such as herring, a paradigm shift from using static geographic limited stock boundaries towards spatial dynamic boundaries may help to support sustainable fisheries management policy in the future. Identification tools as presented here, are essential to achieve this paradigm shift (Cadrin et al. 2023).

A mismatch between geographically defined assessment units, management areas and populations, as observed in this study, is common for highly migratory pelagic species (Fuentes‐Pardo et al. 2023; Quintela et al. 2020). As a migratory pelagic species, it could be expected that herring are not limited by their geographically defined management areas. Therefore, static management paradigms that are often used in fisheries management worldwide are, and will be, increasingly problematic under projected climate changes, where substantial shifts in distribution of marine populations are expected (Barange et al. 2009; Pecl et al. 2017). Climate‐driven poleward shifts have already been documented for several species (Fossheim et al. 2015; Perry et al. 2005), and can lead to disproportionate harvesting, collapse of especially smaller populations, and consequently the potential extirpation of genetic variation and local adaptations (Utter and Ryman 1993). In the current changing environment, counteracting this becomes more important, as high genetic variation is thought to increase the potential resilience to changing environments (Kardos et al. 2021; Lande 1988; Lande and Shannon 1996).

Whole‐genome sequencing data have shown that herring populations display different evolutionary trajectories linked to their spawning characteristics, show high level of adaptation, and require specific spawning conditions (Han et al. 2020; Pettersson et al. 2019). While herring populations demonstrate homing to well characterized spawning grounds (Moll et al. 2019; Ruzzante et al. 2006) with only limited gene flow (Berg et al. 2021; Pettersson et al. 2023), these populations mix at feeding grounds located at the limit of the Norwegian Sea and surrounding regions where fisheries mainly occur (Pampoulie et al. 2024). Therefore, there is an urgent need to properly characterize the distribution of these herring populations. Real‐time assessment of their composition in fisheries catches will ensure their sustainable exploitation and avoid overexploitation of the smallest populations. An effective management option is to close specific region for the commercial herring fishery to protect the presumed small local and resident herring populations as done for several fjords along the Norwegian coast (Røttingen 1987; Slotte et al. 2016).

In the light of our results, we suggest that the extent of spatial and temporal mixing between herring populations, including the dynamics and distribution patterns, needs further investigation, for example, the northward extension of North Sea autumn‐spawning (NSAS), and Downs herring from the North Sea into the Norwegian Sea. Furthermore, the dynamics of spring‐spawning herring from ICES subdivision 6a (Sp‐6a) in the North Sea and Norwegian Sea needs to be monitored. This group comprises two potential populations: 6aS spring and 6aN spring and neither could be identified in the past, and nor could their contribution to the stock assessments in the respective management areas be evaluated. Theoretically, distinct populations showing similar dynamics and life‐history traits can be treated as a single stock for simplicity and pragmatic reasons (Kerr et al. 2017). On the other hand, this lumping of populations has implications for adjacent management areas where assessment and management are at the population level since the population mortality is not being accounted for (Kell et al. 2009). Nevertheless, such practice requires detailed elaboration of the populations to ensure that they do display similar dynamics and life‐histories. Additionally, close examination of exploitation rates and detailed monitoring is required to ensure that none of the populations are being overexploited (Cadrin et al. 2023). In the present study, although we presented data for up to 5 years, qualitative estimation of mixing over time was not applicable due to the nonrandom selection of mixed‐population samples.

Even though the current genetic baseline needs to be validated further by the inclusion of temporal samples, it has already been shown that several of the selected markers were robust at decadal time scales (Bekkevold et al. 2023; Farrell et al. 2021). For instance, the 53 population samples analyzed by Han et al. (2020) were collected over a 39‐year timeframe (1978–2017), and a PCA analysis based on the 794 most informative genetic markers did not reveal any genetic heterogeneity within populations related to age of sampling. This is in line with previous studies on Atlantic herring (Atmore et al. 2024; Kerr et al. 2019; Larsson et al. 2010) and other marine species (Jorde et al. 2018; Pinsky et al. 2021). Thus, it can be expected that also the additional markers, mainly differentiating local fjord populations, are temporally robust. Despite the robustness of these markers as well as solid populations statistics, a typical shortcoming of the current SNP panel is that it cannot account for populations not included in the proposed baseline. A potential missing population is the 6aS winter‐spawning herring, which is currently the largest population in ICES subdivision 6a (ICES 2023a). To date, the presence of this population in the North Sea has not been robustly evaluated but it has been identified east of the 4° W (Farrell et al. 2021). Therefore, as long as all known herring populations are not included in a baseline, there is a scenario that some of the herring identified as belonging to one of the populations in the baseline actually originates from another unidentified population that has undergone similar selection pressures and thus overlaps genetically. An example of this are 6aS winter‐spawning herring that are currently not included in the presented baseline, which would be if present in the mixed‐population samples likely be misassigned as Downs as they are genetically similar (Farrell et al. 2022; Han et al. 2020). Furthermore, adding baseline populations after the development of the SNP panel, such as the Sp‐6a herring, might result in lower self‐assignment rates because the ability to discriminate them is not optimized. With the applied SNP panel, Sp‐6a herring showed the highest discrepancies between assignment models typically mis‐assigned as NSS, despite the clear genetic differentiation between the two populations (*F*_ST_ = 0.33). Lastly, we have applied the assignment models with their default parameters without optimization, application of assignment thresholds or without a multiple‐step hierarchical approach as done by Farrell et al. (2022), i.e., that each herring was directly assigned to the population with the highest assignment rate. Especially the *assignPOP* model would benefit from being trained and optimized to increase the current agreement of 81% between the two assignment methods. The consequences of discrepancies between assignment models for the stock assessment needs to be investigated in the future.

Similar to the situation described above, the north Atlantic summer‐spawning (NASS) herring were identified as a genetic unit based on the SNP panel consisting of potentially three biologically distinct populations: Icelandic summer‐spawning herring (ISSH), Faroes autumn‐spawning herring (FASH), and Norwegian autumn‐spawning herring (NASH). These three putative populations were not included in the full genome sequencing study (Han et al. 2020) that has been mined to identify the SNPs used in the present study, and as such new markers and future work is needed to provide a final answer about whether NASS constitutes three genetically distinct population or not. However, given that NASS herring were only identified in the mixed‐population samples close to the Norwegian coast in the current study, and not in the area between the Norwegian coast, Iceland, and the Faroe Islands, it is most likely that these fish represent NASH, rather than ISSH or FASH. This finding is also supported by Pampoulie et al. (2024), demonstrating that ISSH occur in the southern coastal areas of Iceland. In addition, whole genome‐sequencing identified small genetic differences between FASH and ISSH, although not clear enough to discriminate them (í Kongsstovu et al. 2022).

### 4.1 Management Implications

This is the first study to use genetic assignment to evaluate the population composition of extensive mixed‐stock catches of Atlantic herring in both the North and Norwegian Seas. Several mismatches between the current management boundaries and population distributions were observed. While the direct implications for the different stocks have been discussed in detail in the Supporting Information, we focus here on the general implications. The most significant of the observed mismatches is that NSAS (up to 84%) and NSS (up to 92%) herring were captured in significant proportions north and south of 62° N, respectively. Clearly, the over 50‐year‐old management boundary separating the *NSAS* and *NSS* stocks at 62° N is insufficient, and therefore, in need of modification. Instead of estimating survey indices per area, one could now estimate abundance indices per population. Furthermore, it should be evaluated which populations can be combined as a managed stock rather than defining stocks by static boundaries. Creating a mixed‐stock fishery zone in this area could provide a potential solution as has been done with the ‘transfer area’ in the eastern part of the North Sea. However, we suggest that in the face of climate change which is modifying the distributions of many species towards the poles, a paradigm shift is now needed where population identification tools are applied to assess and manage herring stocks with dynamic boundaries instead of static geographic areas. Such a regime could be monitored with genetics tools and harvest adjusted “real‐time” as has been done in northern Norway for the opening and shutting of the inner spawning grounds where Northeast Arctic and Norwegian coastal cod may overlap (Dahle et al. 2018). Furthermore, increasing population size led to the expansion of their distribution as observed for NSS herring due to the strong 2016‐yearclass (ICES 2023b), or the occurrence of Sp‐6a herring in the North Sea. In addition, genetically identified NSS herring of this 2016‐yearclass were found in spawning conditions near the coast of Shetland (ICES 2024). This allocation of nontraditional spawning grounds can lead to interbreeding with other spring spawning populations, such as Sp‐6a. The impact on the genetic diversity and population dynamics of such sudden changes in distribution patterns needs to be investigated which was not possible with the current SNP panel. In conclusion, this study provides a tool that should be used in future studies to investigate the effects of population dynamics and to adjust stock management regimes.

## Conflicts of Interest

The authors declare no conflicts of interest.

## Supporting information


Data S1.

## Data Availability

The data that support the findings of this study are openly available at the Dryad Digital Repository: http://doi.org/10.5061/dryad.rfj6q57kb.
