# Supplementary material for: Genetic Stock Identification Reveals Mismatches Between Management Areas and Population Genetic Structure in a Migratory Pelagic Fish
Source: Evol Appl. 2024 Oct 24;17(10):e70030. doi: 10.1111/eva.70030 (PMC11502719; doi:10.1111/eva.70030)
Supplement: Supplementary file 1 — Data S1. [file EVA-17-e70030-s001.zip › eva70030-sup-0001-Supinfo.docx]

**SUPPLEMENTARY MATERIAL**

**MATERIALS AND METHODS**

*Baseline samples*

Baseline samples used in this study described in Farrell *et al.*, (2022) consisted of muscle tissue from spring-spawning herring from the west of Ireland and Britain in ICES subdivision 6a North (6aN). An addition sample of the same spring-spawning population from ICES subdivision 6a South (6aS) near Donegal was genotyped and has not been used in any published work so far. Further, baseline samples described in Bekkevold *et al.*, (2023) were partly used in this study. Samples consisted of tissue or DNA from Baltic Sea autumn-spawning herring from the Gulf of Riga (BASH_GR), Bornholm Basin (BASH_CB), and Greifswald Bay near Rügen (BASH_RU), central Baltic Sea spring spawners from the Gulf of Finland (CBSS_GF) and the Vistula Lagoon near Gdańsk (CBSS_GD), western Baltic spring spawners collected on their main spawning ground near Rügen (WBSS_RU), inner Danish waters (Ringkøbing fjord, WBSS_RF), the Kattegat (WBSS_KA), and the eastern Skagerrak (WBSS_SKE), Downs herring, North Sea autumn spawners (NSAS), and Faroes autumn-spawning herring (FASH). In some cases (e.g., BASH_GR, CBSS_GD, NSAS or Downs), the different samples than originally published in Bekkevold *et al.*, (2023) were used. Thus, the sample size and collection years might vary. However, all samples have been collected in the same spawning grounds from ripe and spawning herring.

*Design of the SNP panel*

The panel used in the study of the main text was developed through the selection of specific SNPs from Han *et al.*, 2020) following the approach described in Bekkevold *et al.*, (2023 supplementary material).

### Reference baseline samples

Along all the process of baseline building, efforts were made to balance the number of individuals per sample to avoid overrepresentation of particular groups. This was particularly the case when merging samples displaying no genetic differentiation. The reference baseline consisted of a total of 1,098 fish collected across 24 sampling locations (Table 1). The first axis of the Principal Component Analysis (PCA) biplot (Fig. S1a), explaining 24.7% of the variance, was mainly driven by spawning time (*i.e.,* autumn spawners on the negative side of the X-axis and spring spawners at X>0). The second PCA axis accounted for 23.5% of the variance and was geographically/environmentally driven. In the Discriminant Analysis of Principal Components (DAPC) built after retaining 40 principal components and 3 discriminant functions, the first and second axes revealed a pattern of differentiation in agreement with the PCA pattern (Fig. S2a-b) whereas the third axis neatly singled out the Atlantic-Pacific hybrids from the remaining samples (Fig. S2b-c). The first dichotomic division in the dendrogram derived from pairwise *F*_ST_ (Fig. S1b) accounted for spawning time with an average differentiation of 0.6 between spring and autumn spawners (when discarding the Pacific hybrids). Some of the samples within each spawners group displaying little to null differentiation were pooled to increase sampling size and therefore statistical power (see Table 1). Pooling of the samples was conducted following the ramification of the *F*_ST_-derived dendrogram assisted by geographic information (Table S2). Merging affected the Baltic autumn-spawning herring (BASH), Central Baltic spring spawners (CBSS), western Baltic spring spawners (WBSS), WBSS in Skagerrak, Faroese-Icelandic herring, Atlantic-Pacific hybrids, spring-spawning herring in ICES subarea 6a (Sp-6a), and the local Norwegian fjord populations (except Trondheimsfjorden). Furthermore, the two WBSS_SK samples were kept separated from the remaining WBSS as the *F*_ST_ ranged between 0.095 and 0.334 (Table S2).

The PCA biplot resulting of the merged samples (Fig. S3a) reflected their distinctness in agreement with pairwise *F*_ST_ matrix (Fig. S3b). In addition to spawning time being a major driver of differentiation, the first and second DAPC axes (Fig. S4a-b) also reflected geographic distance between sampling areas unlike the third axis, which again, separated the Pacific hybrids (Fig. S4b-c).

### Potentially new baseline samples

Fish collected in different spawning locations were divided according to spawning time using a combination of biological and genetic information. In a first step, STRUCTURE was conducted at K=2 on a subset of 577 individuals for which the maturity stage was available to discriminate between spring and non-spring (autumn/winter/summer) spawners. The genetic information was provided by nine informative loci on spawning time (*i.e.* Uher_224, Uher_244, Uher_256, Uher_259, Uher_294, Uher_325, Uher_337, Uher_347, Uher_29_014; see Table S1). A threshold of ancestry of q≥0.70 was chosen to distribute the individuals into their corresponding clusters. Thus, from the 238 individuals that were identified as non-spring spawners using their maturity stage, 191 belonged to cluster 1, 34 belonged to cluster 2 and 13 were left unassigned. Similarly, from the 339 identified spring spawners, 203 belonged to cluster 2, 98 belonged to cluster 1 and 38 did not meet the threshold requirements. This information allowed to calibrate genetic spring spawners (_gss) and genetic autumn spawners (_gas). Hence, the total 802 individuals from potentially new baseline samples (Table 3) were divided into 306 autumn spawners, 452 spring spawners and 44 non-assigned individuals, which were thus discarded from further analyses. Likewise, samples with N≤4 were also disregarded from any analysis due to the lack of statistical power. The PCA biplot (Fig. S5a) illustrates that spawning time accounts for 87.2% of the observed variation.

The differentiation between spring and autumn spawners ranged between *F_ST_* 0.43 and 0.81 (Table S4), and the ramification of the *F*_ST_-based dendrogram aided to pool those fjord samples displaying low-null differentiation (Fig. S5b). All spring-spawning samples were merged into a single unit called “Fjord_gss” due to their low/null levels of differentiation. Within autumn spawners, NASH_gas was highly differentiated from the remaining samples *F_ST_* ≥ 0.35 and was therefore kept separately. The remaining seven autumn spawners samples (*F_ST_* ranging between 0.000 and 0.04) were pooled together under the name of “Fjord_gas”.

### Building the final baseline

The datasets corresponding to the known reference baseline, the fjord spring spawners and the fjord autumn spawners were jointly analysed (Fig. S6, Table S5). Very low differentiation was registered between baseline sample NSS and Fjord_gss (*F*_ST_=0.03), thus the fjord sample was dismissed. Likewise, the Fjord_gas sample was not significantly different from baseline sample NSAS (*F*_ST_=0.000, *P*=0.94) and therefore the redundant fjord sample was also discarded from the final version of the baseline. Despite being significantly different from zero, the comparatively low levels of differentiation recorded between FASH_ISSH and NASH_gas allowed merging them as North Atlantic summer/autumn spawners (NASS). Pairwise *F*_ST_ (Table S6), genetic diversity per sample (Table S7), and all graphical outputs (see Fig. S7 to S10) were produced after balancing out the sampling sizes. All samples in the baseline are highly differentiated from each other with *F*_ST_ ranging from 0.12 to 0.87 (Table S6).

### Comparison between rubias and assingPOP

The two different assignment methods in general had a high overall agreement (81.0%; Table S10 and Fig. 4). There was almost no discrepancy for herring assigned to populations from the brackish Baltic Sea and their transition zone. The biggest discrepancy between the two models occurred when assigning autumn/winter-spawning herring from NSAS, Downs, and NASS, and between spring-spawning herring from NSS and Sp-6a which also clustered according to the PCA (Fig. 3). However, this might be an artefact of not having the most informative markers selected for the panel. For these discrepancies, *rubias* clearly favors NSAS instead of Downs, whereas *assignPOP* favors Sp-6a over NSS (Table S10).

**DISCUSSION**

*Norwegian Sea and the Norwegian coastline*

The Norwegian Sea is the main management area of the *NSS* herring stock, which has been assumed to consist of NSS herring almost exclusively. Additionally, the management area of the *NSS* herring stock extends into Icelandic waters where it overlaps with the management area of the *Icelandic summer-spawning herring (ISSH)* stock (Fig 1.). Here, we clearly demonstrate that other populations are mixed with NSS herring during survey periods and since no population identification is applied during the data collection, all populations combined are included in the biomass estimate of the *NSS* herring stock. Especially notable are significant amounts of NSAS and Downs herring north of the management boundary at 62°N. Mixed-population samples in the southern part of the Norwegian Sea rarely have more than 25% NSS herring. Other populations, such as NASH near Lofoten (Husebø et al. 2005) and local fjord populations (Pampoulie et al. 2015; Pettersson et al. 2023), have been known to exist within the geographical delimitation of this stock. With the established genetic baseline, all biological units can now for the first time be identified from mixed-population samples. The actual abundance of these populations is unknown, and they have so far been neglected in the stock assessment.

The local fjord herring, Trondheimsfjorden (THF) herring and Pacific herring hybrid populations (Balsfjorden and Rossfjordvannet) have not been identified in mixed-population samples, except neglectable amounts of THF herring (<1%) according to *assignPOP*. The local fjord herring are resident populations most likely not leaving the fjords (Berg et al. 2017), with no or limited gene flow to NSS herring. However, Lindås herring and THF herring are known to migrate along the coast potentially forming a metapopulation with NSS herring (Johannessen et al. 2014; Sørensen 2012). Still, lack of interbreeding is supported by genetics, as they otherwise would lack a distinct genetic profile. As such, there is no need for the stock assessment to consider these resident fjord populations.

*North Sea and adjacent waters*

In the North Sea, three main spawning grounds have been defined for NSAS herring and one for winter-spawning Downs herring (Payne 2010). There seems to be no genetic difference between among NSAS spawning grounds, whereas Downs herring are a separate genetically distinct population (Bekkevold et al. 2023; Han et al. 2020). It has been assumed that NSAS is the main population contributing to the *NSAS* herring stock. However, the genetically highly distinct Downs herring are assessed as part of this stock, and their dynamics have been monitored for the stock assessment based on larval indices (ICES 2023a). Using genetics, we will in the future be able to monitor the population dynamics of juveniles and adults throughout the management areas. There seems to be a tendency of increasing abundance of Downs during the HERAS surveys from 2019-2023 based on the genetic samples analyzed (Suppl. Fig. 14-15), which needs to be verified in future studies estimating the actual abundance. This increase might be linked to climate change and consequently warmer temperatures during spawning which are favorable for winter-spawners (Toomey et al. 2023). Further work is also required to robustly assess the presence of 6aS winter-spawning herring, which may form part of the herring assigned as Down herring in the mixed samples in the current analysis.

Spring-spawning herring have previously been documented both on the eastern (Berg et al. 2021; Mueller et al. 2023) and western side of the North Sea as well as west of Scotland and northwest of Ireland (Frost and Diele 2022). In the current study, Sp-6a herring typically occurring west of Scotland and northwest of Ireland were identified for the first time in the entire North Sea and even north for 62°N. This population was not identified in mixed-population samples from the North Sea in 2019, but only appear in samples from 2020 and later (Suppl. Fig. 14-15). Their population dynamics such as year-class strength need to be investigated since it seems that this population is contributing a considerable proportion of the herring caught in the commercial fisheries and scientific cruises in this region in the latest years. The autumn-spawning herring in ICES subdivision 6aN off the north of Scotland and the North Sea belong to the same population (Farrell et al. 2022). Newly autumn-hatched larvae from this region drift towards the nursery grounds in the eastern North Sea (Corten 2013). It needs to be investigated if larvae of spring-spawning herring from subdivision 6a also drifted into the North Sea and utilize the same nursery grounds as NSAS herring explaining. The presence of the Sp-6a population in the North Sea was previously unknown. Using the genetic population identification method established in this study and considering their caveats, the proportion of herring identified as Sp-6a can now be monitored and their potential effects on the *NSAS* herring stock assessment evaluated. However further work is needed to refine the ability to discriminate between these herring and the NSS.

Another spring-spawning population is NSS herring, occurring with increasing numbers in the North Sea from 2019-2023 both during the HERAS and in commercial catches. This southward extension is linked to the strong 2016-yearclass (ICES 2023b) and may be an indication of reutilization of former NSS spawning grounds along the southwest coast of Norway (Berg et al. 2017; Dragesund et al. 1997). Spawning NSS herring have in recent times occasionally been observed and identified along the southern Norwegian coast, in the Skagerrak (Eggers et al. 2014), and around Shetland (ICES 2024).

Herring typically associated with the *WBSS* herring stock are known to migrate into the North Sea during their summer feeding period (Bekkevold et al. 2023; Berg et al. 2017), and are in data collection separated from the *NSAS* stock in the ‘transfer-area’ for the stock assessments (Fig. 1). In this study, we provide the first evidence of WBSS herring occurring in non-negligible proportions outside the ‘transfer-area’ in the North Sea. The *WBSS* herring stock is comprised of several spring-spawning herring populations (e.g., WBSS and WBSS-SK; Bekkevold *et al.*, 2023). Similarly, we also identified other Baltic Sea herring populations (BASH and CBSS) not only in the ‘transfer-area’, but also in the management area of the *NSAS* herring stock.

Discrepancies between stocks and populations may impact the perception of stock status. For the *WBSS* herring stock, which currently is below Blim, meaning having reduced reproductive capacity, and 10-times less abundant than the *NSAS* herring stock, such discrepancies can have major detrimental impact. The total amount of herring catches in the ‘transfer-area’ in 2022 exceeded the estimated abundance of the entire *WBSS* herring stock (ICES 2023a). This highlights the need for precise identification methods and adequate definition of management areas. Further studies are needed to assess the actual impact of misassignments on individual stocks, as e.g., 1% mis-assigned WBSS herring might have no impact to the *NSAS* herring stock, but vice versa can have huge implications.

**REFERENCES**

Bekkevold, D., Berg, F., Polte, P., Bartolino, V., Ojaveer, H., Mosegaard, H., et al. (2023). Mixed-stock analysis of Atlantic herring (*Clupea harengus*): a tool for identifying management units and complex migration dynamics. *ICES Journal of Marine Science*, *80*(1), 173–184. https://doi.org/10.1093/icesjms/fsac223

Berg, F., Slotte, A., Johannessen, A., Kvamme, C., Clausen, L. W., & Nash, R. D. M. (2017). Comparative biology and population mixing among local, coastal and offshore Atlantic herring (*Clupea harengus*) in the North Sea, Skagerrak, Kattegat and western Baltic. *PLOS ONE*, *12*(10), e0187374. https://doi.org/10.1371/journal.pone.0187374

Berg, F., Østgaard, H. D., Slotte, A., Andersson, L., & Folkvord, A. (2021). A combination of genetic and phenotypic characterization of spring- and autumn-spawning herring suggests gene flow between populations. *ICES Journal of Marine Science*, *78*(2), 694–703. https://doi.org/10.1093/icesjms/fsaa046

Corten, A. (2013). Recruitment depressions in North Sea herring. *ICES Journal of Marine Science*, *70*(1), 1–15. https://doi.org/10.1093/icesjms/fss187

Dragesund, O., Johannessen, A., & Ulltang, Ø. (1997). Variation in migration and abundance of Norwegian spring spawning herring (*Clupea harengus* L.). *Sarsia*, *82*(2), 97–105. https://doi.org/10.1080/00364827.1997.10413643

Eggers, F., Slotte, A., Libungan, L. A., Johannessen, A., Kvamme, C., Moland, E., et al. (2014). Seasonal Dynamics of Atlantic Herring (*Clupea harengus* L.) Populations Spawning in the Vicinity of Marginal Habitats. *PLOS ONE*, *9*(11), e111985. https://doi.org/10.1371/journal.pone.0111985

Farrell, E. D., Andersson, L., Bekkevold, D., Campbell, N., Carlsson, J., Clarke, M. W., et al. (2022). A baseline for the genetic stock identification of Atlantic herring, *Clupea harengus*, in ICES Divisions 6.a, 7.b–c. *R. Soc. Open Sci.*, *9*(9). https://doi.org/10.1098/rsos.220453

Frost, M., & Diele, K. (2022). Essential spawning grounds of Scottish herring: current knowledge and future challenges. *Reviews in Fish Biology and Fisheries*, *32*, 721–744. https://doi.org/10.1007/s11160-022-09703-0

Han, F., Jamsandekar, M., Pettersson, M. E., Su, L., Fuentes-Pardo, A. P., Davis, B. W., et al. (2020). Ecological adaptation in Atlantic herring is associated with large shifts in allele frequencies at hundreds of loci. *eLife*, *9*(e61076). https://doi.org/10.7554/elife.61076

Husebø, Å., Slotte, A., Clausen, L. A. W., & Mosegaard, H. (2005). Mixing of populations or year class twinning in Norwegian spring spawning herring? *Marine and Freshwater Research*, *56*(5), 763–772. https://doi.org/10.1071/MF04170

ICES. (2023a). *HERRING ASSESSMENT WORKING GROUP FOR THE AREA SOUTH OF 62° N (HAWG)* (No. 5:23) (pp. 1–850). Copenhagen: ICES. https://doi.org/10.17895/ices.pub.22182034

ICES. (2023b). *Working Group of International Pelagic Surveys (WGIPS)* (No. 5:74) (p. 122). Copenhagen: ICES. https://doi.org/10.17895/ices.pub.23607303

ICES. (2024). *Second Workshop on Stock Identification and Allocation of Catches of Herring to Stocks (WKSIDAC2; outputs from 2023 meeting)* (No. 6:5) (p. 67). Copenhagen: ICES. https://doi.org/10.17895/ices.pub.24998747

Johannessen, A., Skaret, G., Langård, L., Slotte, A., Husebø, Å., & Fernö, A. (2014). The Dynamics of a Metapopulation: Changes in Life-History Traits in Resident Herring that Co-Occur with Oceanic Herring during Spawning. *PLoS ONE*, *9*(7), e102462. https://doi.org/10.1371/journal.pone.0102462

Mueller, J., dos Santos Schmidt, T. C., Seljestad, G. W., Clemmesen, C., Gröger, J. P., & Berg, F. (2023). Analysis of reproductive traits reveals complex population dynamics on a small geographical scale in Atlantic herring. *Frontiers in Marine Science*, *10*. https://doi.org/10.3389/fmars.2023.978694

Pampoulie, C., Slotte, A., Óskarsson, G. J., Helyar, S. J., Jónsson, Á., Ólafsdóttird, G., et al. (2015). Stock structure of Atlantic herring *Clupea harengus* in the Norwegian Sea and adjacent waters. *MEPS*, *522*, 219–230. https://doi.org/10.3354/meps11114

Payne, M. R. (2010). Mind the gaps: a state-space model for analysing the dynamics of North Sea herring spawning components. *ICES Journal of Marine Science*, *67*(9), 1939–1947. https://doi.org/10.1093/icesjms/fsq036

Pettersson, M. E., Fuentes-Pardo, A. P., Rochus, C. M., Enbody, E. D., Bi, H., Väinölä, R., & Andersson, L. (2023). A Long-Standing Hybrid Population Between Pacific and Atlantic Herring in a Subarctic Fjord of Norway. *Genome Biology and Evolution*, *15*(5). https://doi.org/10.1093/gbe/evad069

Sørensen, O. B. (2012). Comparative biology and population dynamics between Trondheimsfjord herring and Norwegian spring spawning herring, implications for management (Master’s thesis). University of Bergen, Bergen, Norway.

Toomey, L., Giraldo, C., Loots, C., Mahé, K., Marchal, P., & MacKenzie, K. (2023). Impact of temperature on Downs herring (*Clupea harengus*) embryonic stages: First insights from an experimental approach. *PLOS ONE*, *18*(4), e0284125. https://doi.org/10.1371/JOURNAL.PONE.0284125

**TABLES and FIGURES**

**Table S1.** Overview of SNP markers used in the final panel. Including number and position on chromosome, primer name, splitting information, and flanking region.

See Supplementary table excel file

**Table S2**. Reference baseline: Genetic differentiation between geographically explicit sampling sites. Heatmap of pairwise *F*_ST_ assessed for the total 59 SNPs (lower diagonal) and *P*-values after 10,000 permutations (upper diagonal). Green colours indicate low differentiation and increase towards red to show larger differentiation. Boldface fonts depict significant differentiation after FDR correction. Samples displaying null to very low genetic differentiation will be merged for subsequent analyses.

|  | **BALSFJ** | **ROSSFJ** | **BASH-RU** | **BASH-CB** | **BASH-GR** | **Sp-6aN** | **Sp-6aS** | **NSS** | **ISSH** | **FASH** | **NSAS** | **Downs** | **THF** | **Lindas** | **LUFJ** | **SOGNEFJ** | **GLOPFJ** | **CBSS-GD** | **CBSS-GF** | **WBSS-RU** | **WBSS-KA** | **WBSS-RF** | **WBSS-SKE** | **WBSS-SKW** |
| --- | --- | --- | --- | --- | --- | --- | --- | --- | --- | --- | --- | --- | --- | --- | --- | --- | --- | --- | --- | --- | --- | --- | --- | --- |
| **BALSFJ** | ***** | 0.071 | **0.000** | **0.000** | **0.000** | **0.000** | **0.000** | **0.000** | **0.000** | **0.000** | **0.000** | **0.000** | **0.000** | **0.000** | **0.000** | **0.000** | **0.000** | **0.000** | **0.000** | **0.000** | **0.000** | **0.000** | **0.000** | **0.000** |
| **ROSSFJ** | 0.023 | ***** | **0.000** | **0.000** | **0.000** | **0.000** | **0.000** | **0.000** | **0.000** | **0.000** | **0.000** | **0.000** | **0.000** | **0.000** | **0.000** | **0.000** | **0.000** | **0.000** | **0.000** | **0.000** | **0.000** | **0.000** | **0.000** | **0.000** |
| **BASH-RU** | 0.686 | 0.700 | ***** | 0.148 | **0.000** | **0.000** | **0.000** | **0.000** | **0.000** | **0.000** | **0.000** | **0.000** | **0.000** | **0.000** | **0.000** | **0.000** | **0.000** | **0.000** | **0.000** | **0.000** | **0.000** | **0.000** | **0.000** | **0.000** |
| **BASH-CB** | 0.715 | 0.718 | 0.008 | ***** | **0.000** | **0.000** | **0.000** | **0.000** | **0.000** | **0.000** | **0.000** | **0.000** | **0.000** | **0.000** | **0.000** | **0.000** | **0.000** | **0.000** | **0.000** | **0.000** | **0.000** | **0.000** | **0.000** | **0.000** |
| **BASH-GR** | 0.667 | 0.669 | 0.068 | 0.054 | ***** | **0.000** | **0.000** | **0.000** | **0.000** | **0.000** | **0.000** | **0.000** | **0.000** | **0.000** | **0.000** | **0.000** | **0.000** | **0.000** | **0.000** | **0.000** | **0.000** | **0.000** | **0.000** | **0.000** |
| **Sp-6aN** | 0.754 | 0.776 | 0.673 | 0.715 | 0.679 | ***** | 0.139 | **0.000** | **0.000** | **0.000** | **0.000** | **0.000** | **0.000** | **0.000** | **0.000** | **0.000** | **0.000** | **0.000** | **0.000** | **0.000** | **0.000** | **0.000** | **0.000** | **0.000** |
| **Sp-6aS** | 0.710 | 0.734 | 0.617 | 0.674 | 0.643 | 0.011 | ***** | **0.000** | **0.000** | **0.000** | **0.000** | **0.000** | **0.000** | **0.000** | **0.000** | **0.000** | **0.000** | **0.000** | **0.000** | **0.000** | **0.000** | **0.000** | **0.000** | **0.000** |
| **NSS** | 0.875 | 0.879 | 0.822 | 0.830 | 0.799 | 0.386 | 0.429 | ***** | **0.000** | **0.000** | **0.000** | **0.000** | **0.000** | **0.000** | **0.000** | **0.000** | **0.000** | **0.000** | **0.000** | **0.000** | **0.000** | **0.000** | **0.000** | **0.000** |
| **ISSH** | 0.775 | 0.771 | 0.539 | 0.557 | 0.493 | 0.697 | 0.641 | 0.754 | ***** | 0.845 | **0.000** | **0.000** | **0.000** | **0.000** | **0.000** | **0.000** | **0.000** | **0.000** | **0.000** | **0.000** | **0.000** | **0.000** | **0.000** | **0.000** |
| **FASH** | 0.787 | 0.785 | 0.537 | 0.550 | 0.480 | 0.712 | 0.649 | 0.781 | 0.000 | ***** | **0.000** | **0.000** | **0.000** | **0.000** | **0.000** | **0.000** | **0.000** | **0.000** | **0.000** | **0.000** | **0.000** | **0.000** | **0.000** | **0.000** |
| **NSAS** | 0.758 | 0.753 | 0.567 | 0.590 | 0.564 | 0.649 | 0.573 | 0.818 | 0.565 | 0.563 | ***** | **0.000** | **0.000** | **0.000** | **0.000** | **0.000** | **0.000** | **0.000** | **0.000** | **0.000** | **0.000** | **0.000** | **0.000** | **0.000** |
| **Downs** | 0.803 | 0.792 | 0.618 | 0.629 | 0.625 | 0.722 | 0.661 | 0.841 | 0.673 | 0.675 | 0.183 | ***** | **0.000** | **0.000** | **0.000** | **0.000** | **0.000** | **0.000** | **0.000** | **0.000** | **0.000** | **0.000** | **0.000** | **0.000** |
| **THF** | 0.562 | 0.592 | 0.611 | 0.654 | 0.637 | 0.411 | 0.419 | 0.527 | 0.604 | 0.599 | 0.647 | 0.708 | ***** | **0.000** | **0.000** | **0.000** | **0.000** | **0.000** | **0.000** | **0.000** | **0.000** | **0.000** | **0.000** | **0.000** |
| **Lindas** | 0.626 | 0.662 | 0.625 | 0.678 | 0.661 | 0.345 | 0.328 | 0.663 | 0.690 | 0.681 | 0.621 | 0.699 | 0.154 | ***** | 0.101 | 0.861 | 0.712 | **0.000** | **0.000** | **0.000** | **0.000** | **0.000** | **0.000** | **0.000** |
| **LUFJ** | 0.615 | 0.640 | 0.630 | 0.674 | 0.665 | 0.378 | 0.368 | 0.634 | 0.659 | 0.651 | 0.591 | 0.662 | 0.179 | 0.007 | ***** | 0.074 | 0.881 | **0.000** | **0.000** | **0.000** | **0.000** | **0.000** | **0.000** | **0.000** |
| **SOGNEFJ** | 0.691 | 0.724 | 0.689 | 0.726 | 0.699 | 0.403 | 0.379 | 0.692 | 0.739 | 0.742 | 0.683 | 0.750 | 0.091 | 0.000 | 0.010 | ***** | 0.303 | **0.000** | **0.000** | **0.000** | **0.000** | **0.000** | **0.000** | **0.000** |
| **GLOPFJ** | 0.668 | 0.715 | 0.662 | 0.712 | 0.685 | 0.462 | 0.419 | 0.766 | 0.763 | 0.767 | 0.685 | 0.755 | 0.132 | 0.000 | 0.000 | 0.000 | ***** | **0.000** | **0.000** | **0.000** | **0.000** | **0.000** | **0.000** | **0.000** |
| **CBSS-GD** | 0.625 | 0.659 | 0.567 | 0.620 | 0.569 | 0.587 | 0.575 | 0.752 | 0.734 | 0.731 | 0.736 | 0.789 | 0.568 | 0.574 | 0.590 | 0.621 | 0.591 | ***** | **0.000** | **0.000** | **0.000** | **0.000** | **0.000** | **0.000** |
| **CBSS-GF** | 0.651 | 0.679 | 0.584 | 0.627 | 0.559 | 0.659 | 0.643 | 0.783 | 0.738 | 0.738 | 0.761 | 0.811 | 0.602 | 0.636 | 0.642 | 0.681 | 0.660 | 0.070 | ***** | **0.000** | **0.000** | **0.000** | **0.000** | **0.000** |
| **WBSS-RU** | 0.587 | 0.621 | 0.511 | 0.569 | 0.524 | 0.577 | 0.565 | 0.733 | 0.700 | 0.697 | 0.713 | 0.768 | 0.547 | 0.561 | 0.579 | 0.606 | 0.577 | 0.037 | 0.137 | ***** | **0.000** | **0.000** | **0.000** | **0.000** |
| **WBSS-KA** | 0.518 | 0.557 | 0.501 | 0.574 | 0.543 | 0.426 | 0.412 | 0.668 | 0.646 | 0.636 | 0.620 | 0.695 | 0.493 | 0.437 | 0.477 | 0.485 | 0.442 | 0.139 | 0.266 | 0.110 | ***** | 0.652 | **0.000** | **0.000** |
| **WBSS-RF** | 0.513 | 0.553 | 0.477 | 0.556 | 0.531 | 0.390 | 0.378 | 0.652 | 0.637 | 0.625 | 0.604 | 0.683 | 0.481 | 0.405 | 0.454 | 0.451 | 0.403 | 0.128 | 0.262 | 0.106 | 0.000 | ***** | **0.000** | **0.000** |
| **WBSS-SKE** | 0.544 | 0.578 | 0.524 | 0.601 | 0.578 | 0.219 | 0.211 | 0.562 | 0.620 | 0.610 | 0.544 | 0.637 | 0.404 | 0.276 | 0.333 | 0.321 | 0.294 | 0.315 | 0.434 | 0.307 | 0.112 | 0.095 | ***** | **0.012** |
| **WBSS-SKW** | 0.601 | 0.646 | 0.609 | 0.669 | 0.630 | 0.270 | 0.268 | 0.582 | 0.694 | 0.698 | 0.655 | 0.732 | 0.336 | 0.282 | 0.326 | 0.351 | 0.334 | 0.349 | 0.461 | 0.334 | 0.138 | 0.118 | 0.022 | ***** |

**Table S3**. Reference baseline: Genetic differentiation after merging geographically explicit sampling sites. Heatmap of pairwise *F*_ST_ assessed for the total 59 SNPs (lower diagonal) and *P*-values after 10,000 permutations (upper diagonal). Green colours indicate low differentiation and increase towards red to show larger differentiation. Boldface fonts depict significant differentiation after FDR correction.

|  | **Pacific-Hybrids** | **BASH** | **Sp-6a** | **NSS** | **FASH_ISSH** | **NSAS** | **Downs** | **THF** | **Local-Fjords** | **CBSS** | **WBSS** | **WBSS-SK** |
| --- | --- | --- | --- | --- | --- | --- | --- | --- | --- | --- | --- | --- |
| **Pacific-Hybrids** | ***** | **0.000** | **0.000** | **0.000** | **0.000** | **0.000** | **0.000** | **0.000** | **0.000** | **0.000** | **0.000** | **0.000** |
| **BASH** | 0.675 | ***** | **0.000** | **0.000** | **0.000** | **0.000** | **0.000** | **0.000** | **0.000** | **0.000** | **0.000** | **0.000** |
| **Sp-6a** | 0.745 | 0.662 | ***** | **0.000** | **0.000** | **0.000** | **0.000** | **0.000** | **0.000** | **0.000** | **0.000** | **0.000** |
| **NSS** | 0.865 | 0.757 | 0.330 | ***** | **0.000** | **0.000** | **0.000** | **0.000** | **0.000** | **0.000** | **0.000** | **0.000** |
| **FASH_ISSH** | 0.773 | 0.494 | 0.672 | 0.750 | ***** | **0.000** | **0.000** | **0.000** | **0.000** | **0.000** | **0.000** | **0.000** |
| **NSAS** | 0.736 | 0.513 | 0.607 | 0.806 | 0.531 | ***** | **0.000** | **0.000** | **0.000** | **0.000** | **0.000** | **0.000** |
| **Downs** | 0.772 | 0.549 | 0.676 | 0.829 | 0.641 | 0.179 | ***** | **0.000** | **0.000** | **0.000** | **0.000** | **0.000** |
| **THF** | 0.600 | 0.655 | 0.445 | 0.544 | 0.642 | 0.643 | 0.702 | ***** | **0.000** | **0.000** | **0.000** | **0.000** |
| **Local-Fjords** | 0.658 | 0.686 | 0.411 | 0.613 | 0.693 | 0.619 | 0.676 | 0.140 | ***** | **0.000** | **0.000** | **0.000** |
| **CBSS** | 0.645 | 0.578 | 0.611 | 0.720 | 0.728 | 0.720 | 0.767 | 0.598 | 0.631 | ***** | **0.000** | **0.000** |
| **WBSS** | 0.533 | 0.521 | 0.445 | 0.575 | 0.624 | 0.592 | 0.649 | 0.505 | 0.512 | 0.122 | ***** | **0.000** |
| **WBSS-SK** | 0.574 | 0.599 | 0.236 | 0.492 | 0.639 | 0.553 | 0.633 | 0.387 | 0.347 | 0.371 | 0.166 | ***** |

**Table S4**. Fjords baseline: Genetic differentiation after merging geographically explicit sampling sites displaying null or very low genetic differentiation. Heatmap of pairwise *F*_ST_ assessed for the total 59 SNPs (lower diagonal) and *P*-values after 10,000 permutations (upper diagonal). Green colours indicate low differentiation and increase towards red to show larger differentiation. Boldface fonts depict significant differentiation after FDR correction. Genetic autumn spawners are indicated with “_gas” whereas genetic spring spawners are indicated with “_gss” endings. Samples displaying null to very low genetic differentiation will be merged for subsequent analyses. Sampling size in samples in red font was ≤7.

|  | **AUFJ_gas** | **DAFJ_gas** | **GURS_gas** | **HJFJ_gas** | **NASH_gas** | **ROMSFJ_gas** | **SYKK_gas** | **VOL_gas** | **AUFJ_gss** | **DAFJ_gss** | **GURS_gss** | **KIRKFJ_gss** | **NASH_gss** | **NSS-SF_gss** | **ROMSFJ_gss** | **SYKK_gss** | **VOL_gss** |
| --- | --- | --- | --- | --- | --- | --- | --- | --- | --- | --- | --- | --- | --- | --- | --- | --- | --- |
| **AUFJ_gas** | * | 0.854 | 0.621 | 0.301 | **0.000** | 0.269 | 0.557 | 0.392 | **0.000** | **0.000** | **0.000** | **0.000** | **0.000** | **0.000** | **0.000** | **0.000** | **0.000** |
| **DAFJ_gas** | 0.000 | * | 0.861 | 0.638 | **0.000** | 0.397 | 0.662 | 0.977 | **0.000** | **0.000** | **0.000** | **0.000** | **0.000** | **0.000** | **0.000** | **0.000** | **0.000** |
| **GURS_gas** | 0.000 | 0.000 | * | 0.428 | **0.000** | 0.190 | 0.182 | 0.550 | **0.000** | **0.000** | **0.000** | **0.000** | **0.000** | **0.000** | **0.000** | **0.000** | **0.000** |
| **HJFJ_gas** | 0.001 | 0.000 | 0.000 | * | **0.000** | 0.309 | **0.000** | 0.819 | **0.000** | **0.000** | **0.000** | **0.000** | **0.000** | **0.000** | **0.000** | **0.000** | **0.000** |
| **NASH_gas** | 0.394 | 0.402 | 0.424 | 0.399 | * | **0.000** | **0.000** | **0.000** | **0.000** | **0.000** | **0.000** | **0.000** | **0.000** | **0.000** | **0.000** | **0.000** | **0.000** |
| **ROMSFJ_gas** | 0.008 | 0.000 | 0.024 | 0.004 | 0.349 | * | 0.060 | 0.080 | **0.000** | **0.000** | **0.000** | **0.000** | **0.000** | **0.000** | **0.000** | **0.000** | **0.000** |
| **SYKK_gas** | 0.000 | 0.000 | 0.005 | 0.022 | 0.483 | 0.044 | * | 0.036 | **0.000** | **0.000** | **0.000** | **0.000** | **0.000** | **0.000** | **0.000** | **0.000** | **0.000** |
| **VOL_gas** | 0.000 | 0.000 | 0.000 | 0.000 | 0.499 | 0.035 | 0.015 | * | **0.000** | **0.000** | **0.000** | **0.000** | **0.000** | **0.000** | **0.000** | **0.000** | **0.000** |
| **AUFJ_gss** | 0.627 | 0.637 | 0.670 | 0.631 | 0.559 | 0.700 | 0.691 | 0.743 | * | 0.052 | 0.453 | 0.052 | 0.163 | 0.999 | 0.992 | 0.159 | 0.053 |
| **DAFJ_gss** | 0.578 | 0.580 | 0.607 | 0.593 | 0.565 | 0.632 | 0.659 | 0.697 | 0.027 | * | 0.041 | **0.000** | **0.000** | 0.263 | **0.009** | 0.047 | 0.112 |
| **GURS_gss** | 0.543 | 0.548 | 0.561 | 0.565 | 0.441 | 0.566 | 0.635 | 0.660 | 0.000 | 0.040 | * | **0.001** | 0.089 | 0.281 | 0.525 | 0.498 | 0.145 |
| **KIRKFJ_gss** | 0.723 | 0.735 | 0.767 | 0.714 | 0.665 | 0.793 | 0.758 | 0.813 | 0.013 | 0.108 | 0.070 | * | 0.044 | **0.002** | 0.396 | **0.009** | **0.001** |
| **NASH_gss** | 0.666 | 0.673 | 0.699 | 0.672 | 0.595 | 0.723 | 0.725 | 0.765 | 0.008 | 0.095 | 0.018 | 0.013 | * | 0.011 | 0.959 | 0.081 | **0.002** |
| **NSS-SF_gss** | 0.561 | 0.554 | 0.561 | 0.587 | 0.495 | 0.532 | 0.649 | 0.634 | 0.000 | 0.007 | 0.008 | 0.037 | 0.027 | * | 0.396 | 0.513 | 0.281 |
| **ROMSFJ_gss** | 0.616 | 0.616 | 0.630 | 0.637 | 0.540 | 0.626 | 0.697 | 0.712 | 0.000 | 0.054 | 0.000 | 0.002 | 0.000 | 0.004 | * | 0.313 | 0.070 |
| **SYKK_gss** | 0.542 | 0.534 | 0.528 | 0.581 | 0.461 | 0.460 | 0.655 | 0.649 | 0.056 | 0.106 | 0.000 | 0.187 | 0.087 | 0.001 | 0.026 | * | 0.749 |
| **VOL_gss** | 0.480 | 0.475 | 0.486 | 0.504 | 0.428 | 0.482 | 0.596 | 0.627 | 0.075 | 0.051 | 0.046 | 0.221 | 0.153 | 0.017 | 0.086 | 0.001 | * |

**Table S5**. Reference and potentially new baseline: Heatmap of pairwise *F*_ST_ assessed for the total 59 SNPs (lower diagonal) and *P*-values after 10,000 permutations (upper diagonal). Green colours indicate low differentiation and increase towards red to show larger differentiation. Boldface fonts depict significant differentiation after FDR correction. Genetic autumn spawners are indicated with “_gas” whereas genetic spring spawners are indicated with “_gss” endings.

|  | **Pacific-Hybrids** | **BASH** | **Sp-6a** | **NSS** | **Fjords_gss** | **FASH_ISSH** | **NASH_gas** | **NSAS** | **Fjords_gas** | **Downs** | **THF** | **Local-Fjords** | **CBSS** | **WBSS** | **WBSS-SK** |
| --- | --- | --- | --- | --- | --- | --- | --- | --- | --- | --- | --- | --- | --- | --- | --- |
| **Pacific-Hybrids** | ***** | **0.000** | **0.000** | **0.000** | **0.000** | **0.000** | **0.000** | **0.000** | **0.000** | **0.000** | **0.000** | **0.000** | **0.000** | **0.000** | **0.000** |
| **BASH** | 0.681 | ***** | **0.000** | **0.000** | **0.000** | **0.000** | **0.000** | **0.000** | **0.000** | **0.000** | **0.000** | **0.000** | **0.000** | **0.000** | **0.000** |
| **Sp-6a** | 0.797 | 0.663 | ***** | **0.000** | **0.000** | **0.000** | **0.000** | **0.000** | **0.000** | **0.000** | **0.000** | **0.000** | **0.000** | **0.000** | **0.000** |
| **NSS** | 0.904 | 0.759 | 0.411 | ***** | **0.000** | **0.000** | **0.000** | **0.000** | **0.000** | **0.000** | **0.000** | **0.000** | **0.000** | **0.000** | **0.000** |
| **Fjords_gss** | 0.863 | 0.735 | 0.246 | 0.028 | ***** | **0.000** | **0.000** | **0.000** | **0.000** | **0.000** | **0.000** | **0.000** | **0.000** | **0.000** | **0.000** |
| **FASH_ISSH** | 0.777 | 0.509 | 0.667 | 0.755 | 0.684 | ***** | 0.001 | **0.000** | **0.000** | **0.000** | **0.000** | **0.000** | **0.000** | **0.000** | **0.000** |
| **NASH_gas** | 0.757 | 0.466 | 0.648 | 0.789 | 0.701 | 0.026 | ***** | **0.000** | **0.000** | **0.000** | **0.000** | **0.000** | **0.000** | **0.000** | **0.000** |
| **NSAS** | 0.763 | 0.562 | 0.629 | 0.840 | 0.771 | 0.556 | 0.474 | ***** | 0.940 | **0.000** | **0.000** | **0.000** | **0.000** | **0.000** | **0.000** |
| **Fjords_gas** | 0.790 | 0.609 | 0.646 | 0.814 | 0.761 | 0.566 | 0.492 | 0.000 | ***** | **0.000** | **0.000** | **0.000** | **0.000** | **0.000** | **0.000** |
| **Downs** | 0.809 | 0.603 | 0.704 | 0.861 | 0.806 | 0.647 | 0.592 | 0.124 | 0.095 | ***** | **0.000** | **0.000** | **0.000** | **0.000** | **0.000** |
| **THF** | 0.619 | 0.661 | 0.471 | 0.570 | 0.542 | 0.628 | 0.602 | 0.654 | 0.706 | 0.712 | ***** | **0.000** | **0.000** | **0.000** | **0.000** |
| **Local-Fjords** | 0.673 | 0.693 | 0.433 | 0.635 | 0.591 | 0.686 | 0.654 | 0.630 | 0.676 | 0.685 | 0.139 | ***** | **0.000** | **0.000** | **0.000** |
| **CBSS** | 0.664 | 0.579 | 0.659 | 0.764 | 0.737 | 0.744 | 0.721 | 0.753 | 0.789 | 0.795 | 0.643 | 0.672 | ***** | **0.000** | **0.000** |
| **WBSS** | 0.532 | 0.514 | 0.468 | 0.593 | 0.573 | 0.616 | 0.580 | 0.609 | 0.662 | 0.662 | 0.542 | 0.550 | 0.135 | ***** | **0.000** |
| **WBSS-SK** | 0.580 | 0.590 | 0.237 | 0.494 | 0.426 | 0.604 | 0.555 | 0.552 | 0.615 | 0.634 | 0.418 | 0.380 | 0.408 | 0.183 | ***** |

**Table S6**. Final baseline: Heatmap of pairwise *F*_ST_ assessed for the total 59 SNPs (lower diagonal) and *P*-values after 10,000 permutations (upper diagonal). Green colours indicate low differentiation and increase towards red to show larger differentiation. Boldface font depict significant differentiation after FDR correction.

|  | **Pacific-Hybrids** | **BASH** | **NASS** | **NSAS** | **Downs** | **THF** | **NSS** | **Sp-6a** | **Local-Fjords** | **CBSS** | **WBSS** | **WBSS-SK** |
| --- | --- | --- | --- | --- | --- | --- | --- | --- | --- | --- | --- | --- |
| **Pacific-Hybrids** | ***** | **0.000** | **0.000** | **0.000** | **0.000** | **0.000** | **0.000** | **0.000** | **0.000** | **0.000** | **0.000** | **0.000** |
| **BASH** | 0.675 | ***** | **0.000** | **0.000** | **0.000** | **0.000** | **0.000** | **0.000** | **0.000** | **0.000** | **0.000** | **0.000** |
| **NASS** | 0.767 | 0.500 | ***** | **0.000** | **0.000** | **0.000** | **0.000** | **0.000** | **0.000** | **0.000** | **0.000** | **0.000** |
| **NSAS** | 0.736 | 0.513 | 0.503 | ***** | **0.000** | **0.000** | **0.000** | **0.000** | **0.000** | **0.000** | **0.000** | **0.000** |
| **Downs** | 0.772 | 0.549 | 0.618 | 0.179 | ***** | **0.000** | **0.000** | **0.000** | **0.000** | **0.000** | **0.000** | **0.000** |
| **THF** | 0.599 | 0.655 | 0.659 | 0.643 | 0.701 | ***** | **0.000** | **0.000** | **0.000** | **0.000** | **0.000** | **0.000** |
| **NSS** | 0.865 | 0.756 | 0.730 | 0.806 | 0.828 | 0.543 | ***** | **0.000** | **0.000** | **0.000** | **0.000** | **0.000** |
| **Sp-6a** | 0.745 | 0.662 | 0.668 | 0.607 | 0.676 | 0.444 | 0.329 | ***** | **0.000** | **0.000** | **0.000** | **0.000** |
| **Local-Fjords** | 0.664 | 0.693 | 0.714 | 0.632 | 0.686 | 0.194 | 0.633 | 0.443 | ***** | **0.000** | **0.000** | **0.000** |
| **CBSS** | 0.645 | 0.578 | 0.739 | 0.720 | 0.767 | 0.597 | 0.719 | 0.611 | 0.642 | ***** | **0.000** | **0.000** |
| **WBSS** | 0.533 | 0.521 | 0.639 | 0.592 | 0.649 | 0.505 | 0.575 | 0.445 | 0.530 | 0.122 | ***** | **0.000** |
| **WBSS-SK** | 0.574 | 0.599 | 0.652 | 0.553 | 0.633 | 0.386 | 0.492 | 0.236 | 0.378 | 0.371 | 0.166 | ***** |

**Table S7**. Genetically distinct populations in the final baseline: Summary statistics including the number of individuals per population (N_Pop), percentage of polymorphic loci (Poly. loci), mean ± standard error of observed (H_o_) and unbiased expected (uH_e_) heterozygosity, as well as inbreeding coefficient (F_IS_).

| **Population** | **N_pop** | **Poly. loci** | ***H*_o_** | **u*H*_e_** | ***F*_IS_** |
| --- | --- | --- | --- | --- | --- |
| Pacific_hybrids | 30 | 64.4 | 0.122 ± 0.022 | 0.125 ± 0.020 | 0.016 ± 0.036 |
| BASH | 102 | 86.4 | 0.191 ± 0.020 | 0.198 ± 0.021 | 0.022 ± 0.016 |
| NASS | 129 | 67.8 | 0.106 ± 0.019 | 0.111 ± 0.019 | 0.054 ± 0.011 |
| NSAS | 60 | 71.2 | 0.121 ± 0.017 | 0.128 ± 0.019 | 0.067 ± 0.031 |
| Downs | 104 | 67.8 | 0.126 ± 0.021 | 0.115 ± 0.018 | -0.022 ± 0.033 |
| THF | 100 | 89.8 | 0.215 ± 0.023 | 0.215 ± 0.021 | 0.007 ± 0.025 |
| NSS | 140 | 71.2 | 0.059 ± 0.014 | 0.055 ± 0.013 | -0.030 ± 0.005 |
| Sp-6a | 58 | 66.1 | 0.129 ± 0.023 | 0.132 ± 0.023 | 0.004 ± 0.018 |
| Local_fjords | 108 | 89.8 | 0.197 ± 0.022 | 0.209 ± 0.021 | 0.056 ± 0.025 |
| CBSS | 82 | 81.4 | 0.195 ± 0.019 | 0.188 ± 0.018 | -0.036 ± 0.016 |
| WBSS | 122 | 91.5 | 0.252 ± 0.024 | 0.250 ± 0.023 | -0.001 ± 0.018 |
| WBSS-SK | 63 | 96.6 | 0.256 ± 0.025 | 0.258 ± 0.024 | 0.002 ± 0.020 |

**Table S8**. Monte-Carlo cross-validation based on assignPOP using a support vector machine (SVM) classification model. Total estimates for combined proportions of training dataset (50%, 70%, and 90%). Total assignment accuracy of the baseline is 89.37%.

| **True pop** | **Predicted population** | | | | | | | | | | | |
| --- | --- | --- | --- | --- | --- | --- | --- | --- | --- | --- | --- | --- |
|  | **BASH** | **CBSS** | **Downs** | **Local-Fjords** | **NASS** | **NSAS** | **NSS** | **Pacific-Hybrids** | **Sp-6a** | **THF** | **WBSS** | **WBSS-SK** |
| BASH | 100 | 0 | 0 | 0 | 0 | 0 | 0 | 0 | 0 | 0 | 0 | 0 |
| CBSS | 0 | 87 | 0 | 0 | 0 | 0 | 0 | 0 | 0 | 0 | 13 | 0 |
| Downs | 0 | 0 | 96.8 | 0 | 0 | 3.2 | 0 | 0 | 0 | 0 | 0 | 0 |
| Local-Fjords | 0 | 0 | 0 | 82.7 | 0 | 0 | 1.7 | 0 | 0 | 15.6 | 0 | 0.01 |
| NASS | 0 | 0 | 0.02 | 0 | 96.8 | 3.2 | 0 | 0 | 0 | 0 | 0 | 0 |
| NSAS | 0 | 0 | 14.6 | 0 | 6.8 | 78.5 | 0 | 0 | 0 | 0 | 0 | 0 |
| NSS | 0 | 0 | 0 | 0 | 0.3 | 0 | 97.5 | 0 | 2.15 | 0 | 0 | 0 |
| Pacific-Hybrids | 0 | 0 | 0 | 0 | 0 | 0 | 0 | 99.9 | 0 | 0.02 | 0 | 0.1 |
| Sp-6a | 0 | 0 | 0.2 | 0 | 0.1 | 1.9 | 13.1 | 0 | 84.8 | 0 | 0 | 0 |
| THF | 0 | 0 | 0 | 17.8 | 0 | 0 | 1.4 | 0 | 1.1 | 79.6 | 0 | 0.1 |
| WBSS | 0 | 12.7 | 0 | 0 | 0 | 0 | 0 | 0 | 0 | 0 | 85.5 | 1.9 |
| WBSS-SK | 0 | 2 | 0 | 0.5 | 0 | 0 | 1.2 | 0 | 1.7 | 0.6 | 10.6 | 83.4 |

**Table S9**. *K*-Fold cross-validation based on assignPOP using a support vector machine (SVM) classification model. Total estimates combined for K = 3, 4, 5, and 15. Total assignment accuracy of the baseline is 89.45%.

| **True pop** | **Predicted population** | | | | | | | | | | | |
| --- | --- | --- | --- | --- | --- | --- | --- | --- | --- | --- | --- | --- |
|  | **BASH** | **CBSS** | **Downs** | **Local-Fjords** | **NASS** | **NSAS** | **NSS** | **Pacific-Hybrids** | **Sp-6a** | **THF** | **WBSS** | **WBSS-SK** |
| BASH | 100 | 0 | 0 | 0 | 0 | 0 | 0 | 0 | 0 | 0 | 0 | 0 |
| CBSS | 0 | 87.2 | 0 | 0 | 0 | 0 | 0 | 0 | 0 | 0 | 12.8 | 0 |
| Downs | 0 | 0 | 95.5 | 0 | 0 | 4.5 | 0 | 0 | 0 | 0 | 0 | 0 |
| Local-Fjords | 0 | 0 | 0 | 83.5 | 0 | 0 | 1.7 | 0 | 0 | 14.8 | 0 | 0 |
| NASS | 0 | 0 | 0 | 0 | 96.9 | 3.1 | 0 | 0 | 0 | 0 | 0 | 0 |
| NSAS | 0 | 0 | 12.8 | 0 | 7.8 | 79.4 | 0 | 0 | 0 | 0 | 0 | 0 |
| NSS | 0 | 0 | 0 | 0 | 0.5 | 0 | 97.3 | 0 | 2.2 | 0 | 0 | 0 |
| Pacific-Hybrids | 0 | 0 | 0 | 0 | 0 | 0 | 0 | 100 | 0 | 0 | 0 | 0 |
| Sp-6a | 0 | 0 | 0 | 0 | 0 | 1.7 | 14 | 0 | 84.3 | 0 | 0 | 0 |
| THF | 0 | 0 | 0 | 17.2 | 0 | 0 | 1.7 | 0 | 1 | 80 | 0 | 0 |
| WBSS | 0 | 13.7 | 0 | 0 | 0 | 0 | 0 | 0 | 0 | 0 | 84.4 | 1.9 |
| WBSS-SK | 0 | 2.6 | 0 | 0 | 0 | 0 | 0 | 0 | 1.6 | 1.6 | 9.3 | 84.9 |

**Table S10**. Assignment results of mixed-stock samples against the established baseline based on assignment models provided by the R packages *assignPOP* and *rubias*. Total agreement between the two assignment methods is 81.0%. Frequency of assigned populations (N %) were estimated for both classification methods for comparison. See Fig. 4 and Table S11 for spatial distribution.

| **assignPOP** | **rubias** | | | | | | | | | | | |  |
| --- | --- | --- | --- | --- | --- | --- | --- | --- | --- | --- | --- | --- | --- |
|  | **BASH** | **CBSS** | **Downs** | **Local-Fjords** | **NASS** | **NSAS** | **NSS** | **Pacific-Hybrids** | **Sp-6a** | **THF** | **WBSS** | **WBSS-SK** | **N (%)** |
| BASH | 70 | 0 | 1 | 0 | 0 | 4 | 0 | 0 | 0 | 0 | 12 | 0 | 0.56 |
| CBSS | 0 | 48 | 0 | 0 | 0 | 0 | 0 | 0 | 0 | 0 | 107 | 0 | 0.99 |
| Downs | 1 | 0 | 4610 | 0 | 0 | 1458 | 0 | 0 | 2 | 0 | 0 | 0 | 38.74 |
| Local-Fjords | 0 | 0 | 0 | 0 | 0 | 0 | 2 | 0 | 11 | 0 | 0 | 2 | 0.10 |
| NASS | 3 | 0 | 1 | 0 | 355 | 128 | 4 | 0 | 12 | 0 | 0 | 0 | 3.21 |
| NSAS | 2 | 0 | 518 | 0 | 0 | 2408 | 0 | 0 | 0 | 0 | 0 | 0 | 18.69 |
| NSS | 0 | 0 | 0 | 0 | 1 | 0 | 2826 | 0 | 24 | 0 | 0 | 0 | 18.19 |
| Pacific-Hybrids | 0 | 0 | 4 | 0 | 0 | 22 | 595 | 0 | 1337 | 0 | 0 | 0 | 0 |
| Sp-6a | 0 | 0 | 0 | 0 | 0 | 0 | 15 | 0 | 1 | 0 | 0 | 0 | 12.50 |
| THF | 0 | 24 | 0 | 0 | 0 | 0 | 0 | 0 | 0 | 0 | 901 | 5 | 0.10 |
| WBSS | 0 | 0 | 0 | 0 | 0 | 0 | 15 | 0 | 26 | 0 | 9 | 106 | 5.93 |
| WBSS-SK | 70 | 0 | 1 | 0 | 0 | 4 | 0 | 0 | 0 | 0 | 12 | 0 | 1.00 |
| N (%) | 0.49 | 0.46 | 32.76 | 0 | 2.27 | 25.65 | 22.06 | 0 | 9.02 | 0 | 6.57 | 0.72 | 100.00 |

**Table S11**. Frequency of assigned populations (N %) in the Norwegian Sea and North Sea (border at 62° N) were estimated for both classification methods, *assignPOP* and *rubias*, for direct comparison. See Fig. 4 for spatial distribution. Note that these numbers do not represent the true distribution because the samples were not randomly selected.

| **Method** | **Sea** | **BASH** | **CBSS** | **Downs** | **Local-Fjords** | **NASS** | **NSAS** | **NSS** | **Sp-6a** | **THF** | **WBSS** | **WBSS-SK** |
| --- | --- | --- | --- | --- | --- | --- | --- | --- | --- | --- | --- | --- |
| *assignPOP* | Norwegian Sea | 0 | 0 | 3.46 | 20.00 | 60.64 | 6.08 | 77.41 | 36.57 | 62.50 | 0 | 5.77 |
|  | North Sea | 100.00 | 100.00 | 96.54 | 80.00 | 39.36 | 93.92 | 22.59 | 63.43 | 37.50 | 100.00 | 94.23 |
|  | **Total N** | **87** | **155** | **6,071** | **15** | **503** | **2,928** | **2,851** | **1,958** | **16** | **930** | **156** |
| *rubias* | Norwegian Sea | 0 | 0 | 3.56 | 0 | 79.78 | 5.57 | 77.96 | 17.83 | 0 | 0 | 0 |
|  | North Sea | 100.00 | 100.00 | 96.44 | 0 | 20.22 | 94.43 | 22.04 | 82.17 | 0 | 100.00 | 100.00 |
|  | **Total N** | **76** | **72** | **5,134** | **0** | **356** | **4,020** | **3,457** | **1,413** | **0** | **1,029** | **113** |

| a)  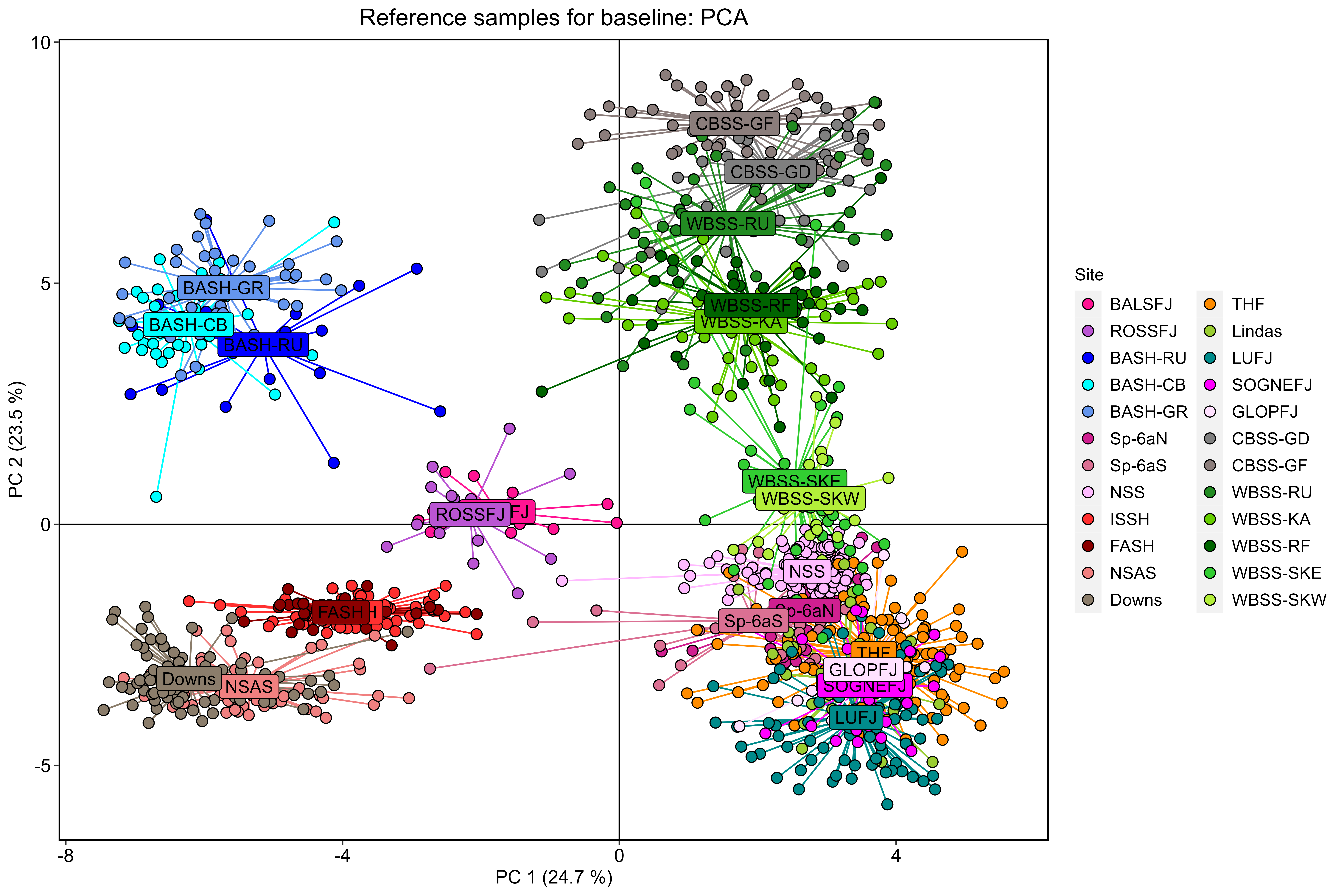 |
| --- |
| b)  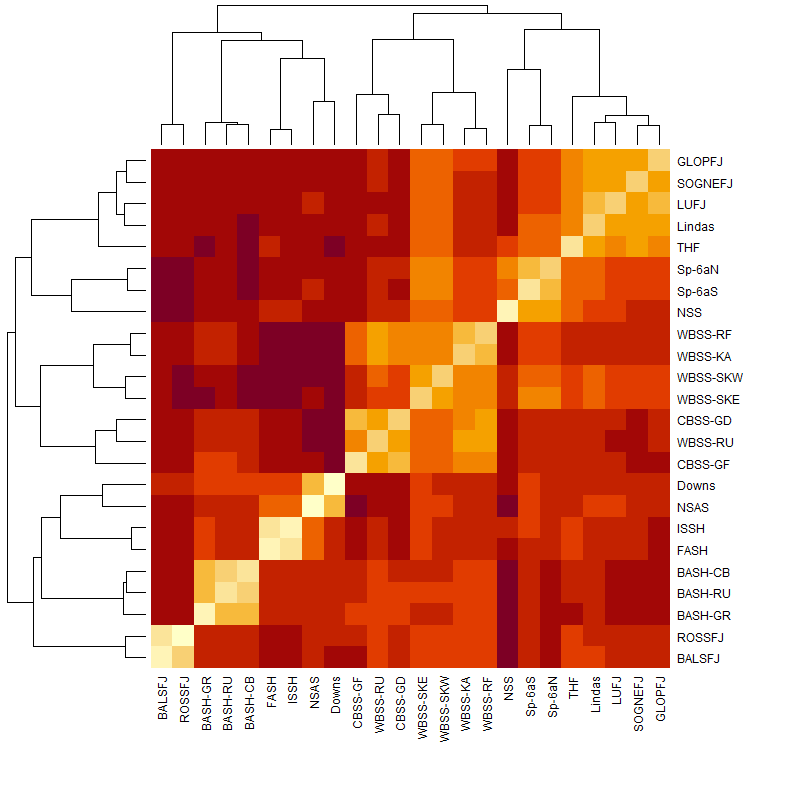 |

**Figure S1**. Reference baseline samples: Principal Component Analysis (PCA) biplot of the fish collected in 24 geographically explicit locations (a) and heatmap of pairwise *F*_ST_ and associated dendrogram (b). Spawning time represents the major driver of the differentiation as displayed in the PCA first axis and in the major dichotomic division of the dendrogram. *F*_ST_ and corresponding P-values can be found in **Table S2**.

| a)  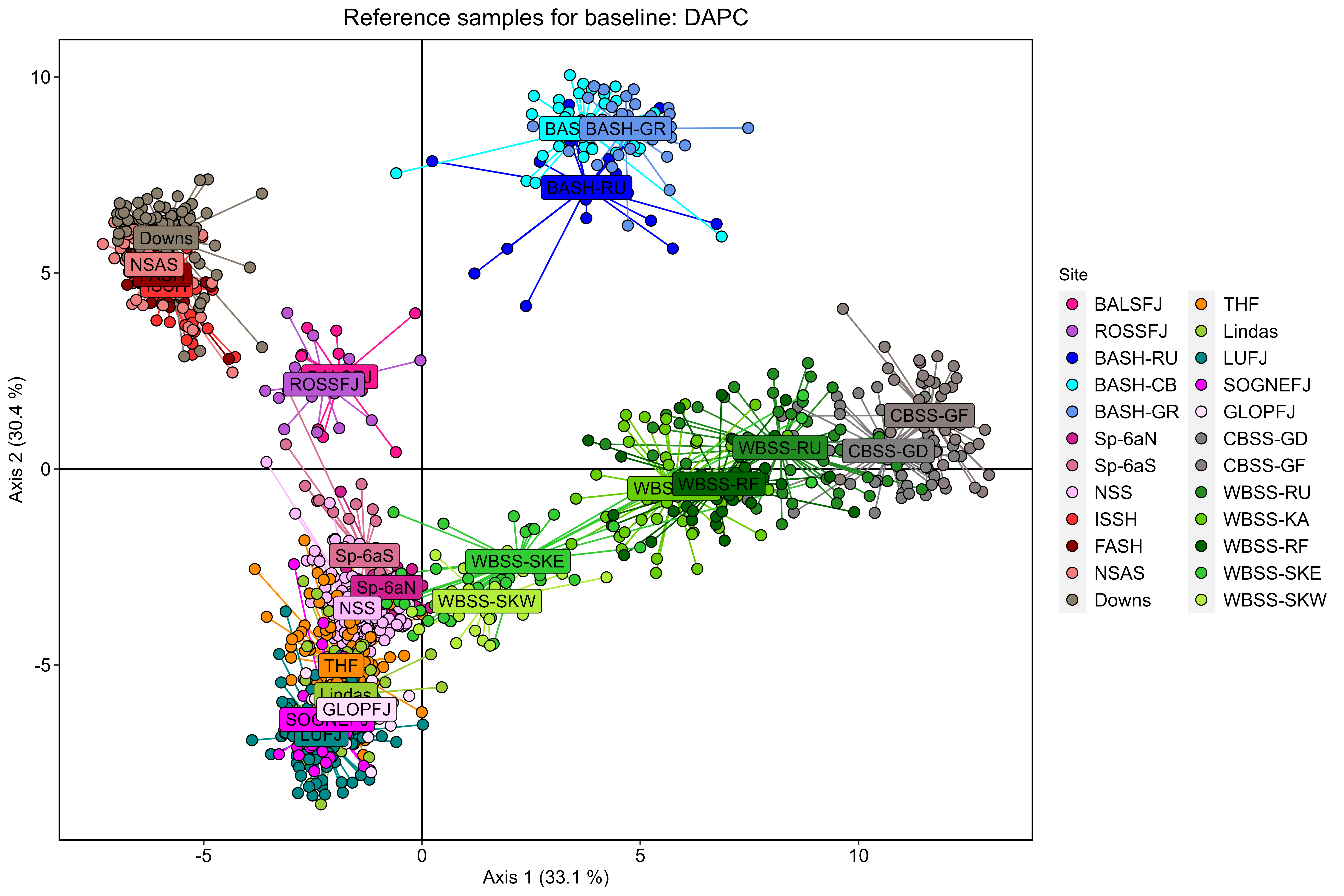 |
| --- |
| b)  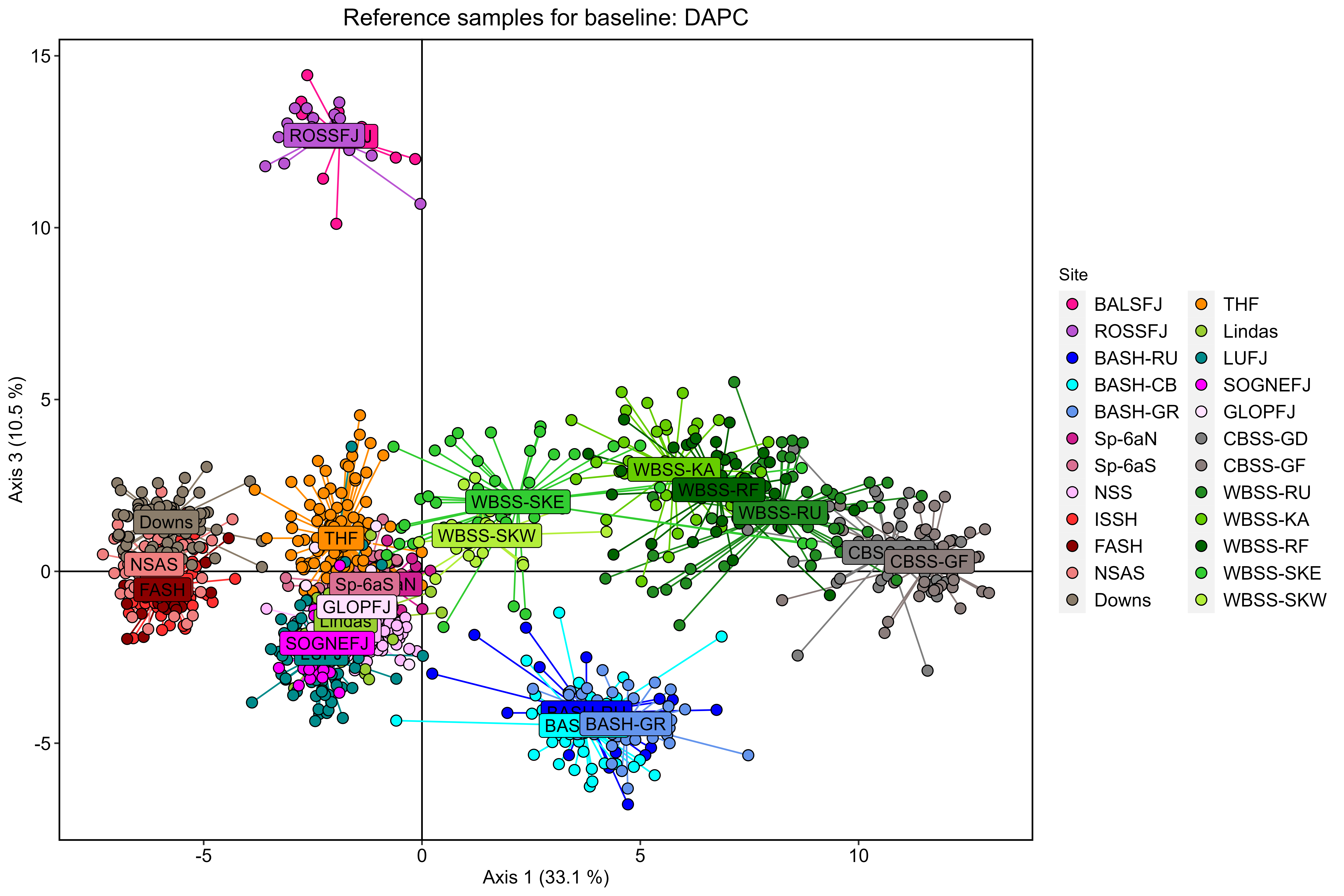 |
| c)  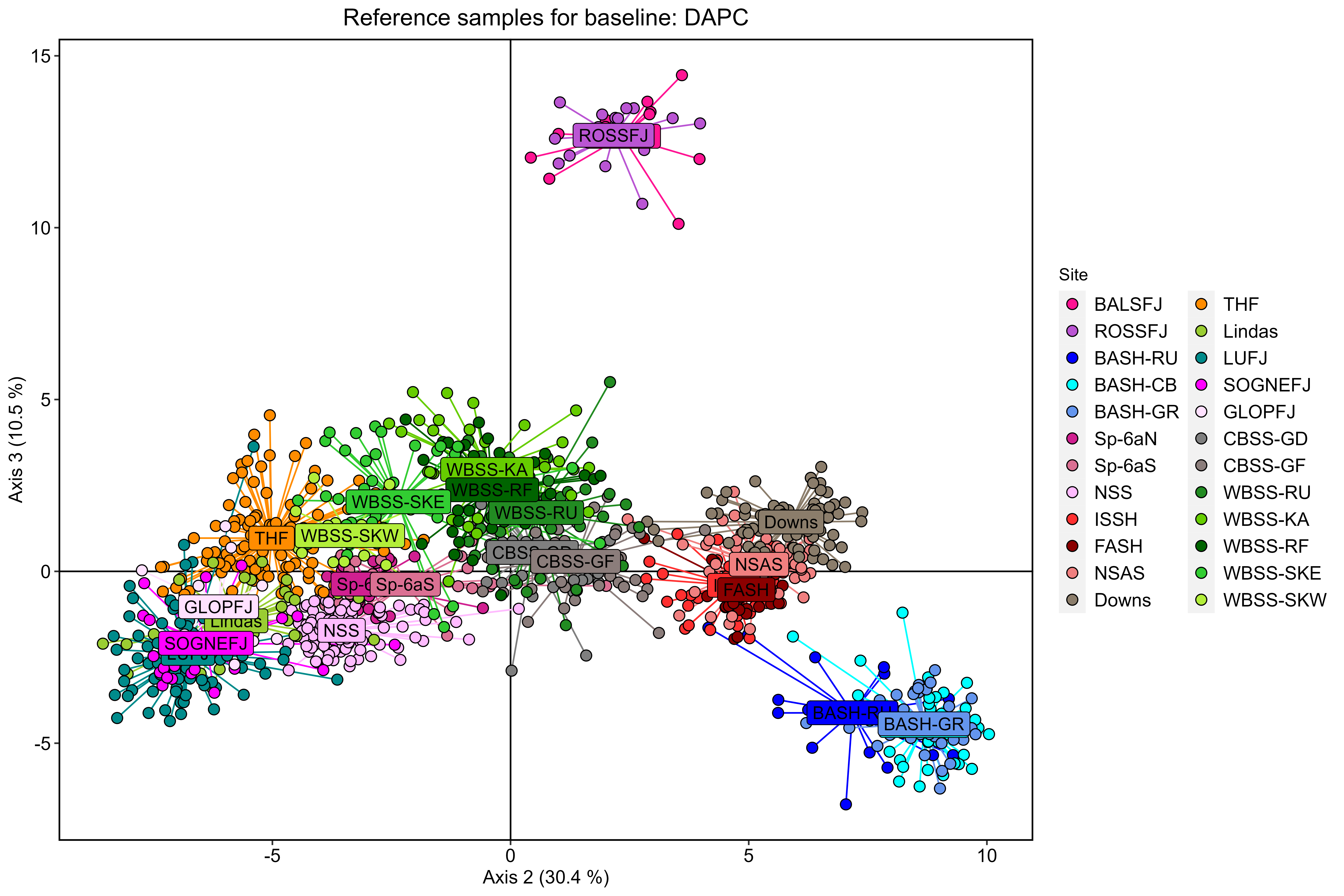 |

**Figure S2**. Reference baseline samples: Discriminant Analysis of Principal Components (DAPC) of the 24 geographically explicit sampling sites built after retaining 40 principal components and 3 discriminant functions.

| a)  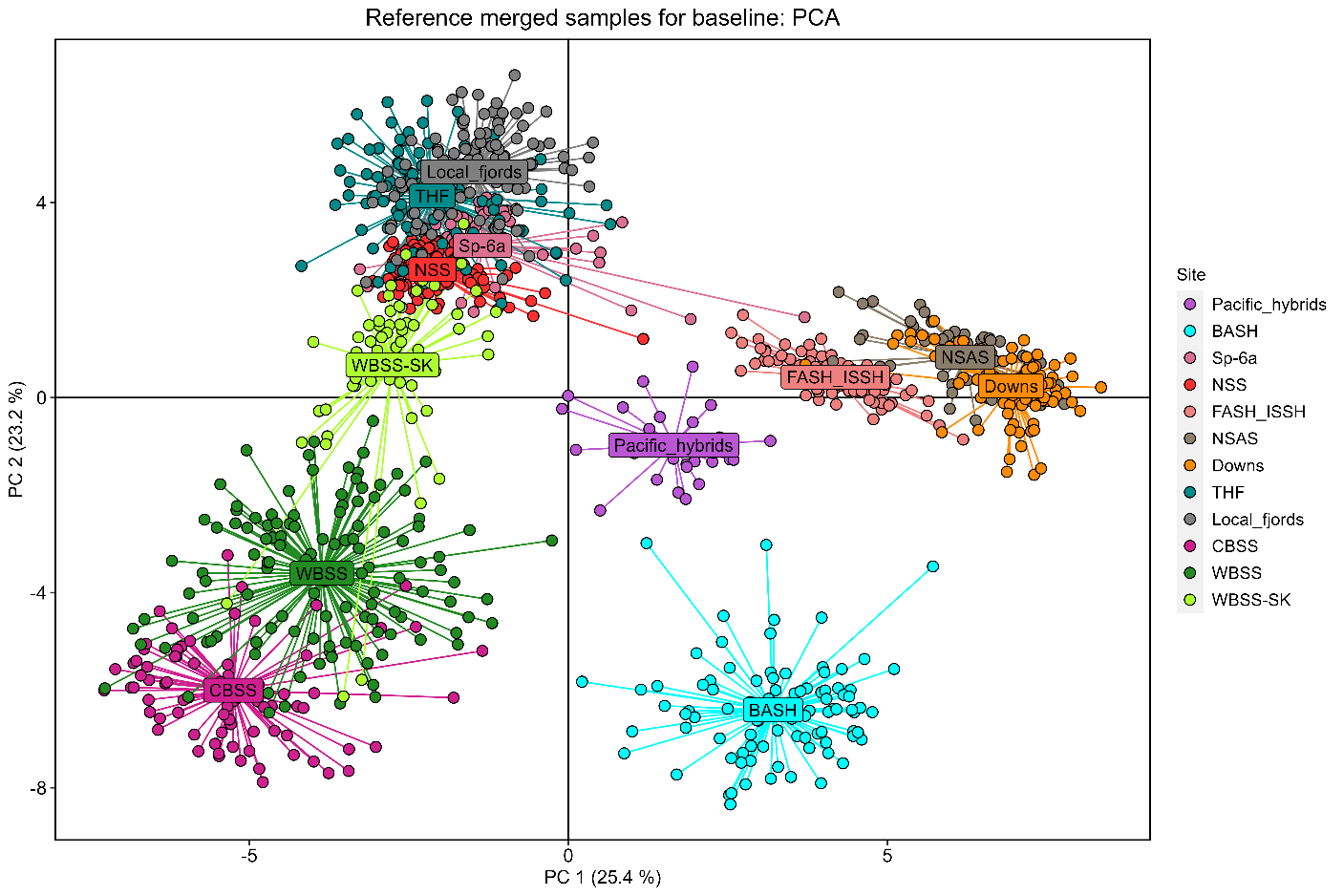 |
| --- |
| b)  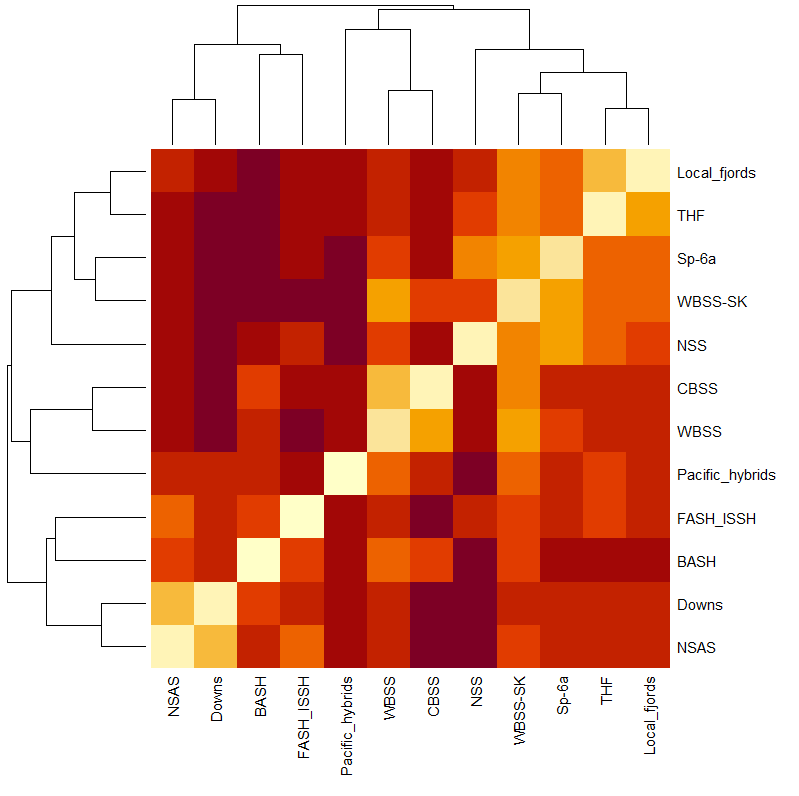 |

**Figure S3.** Reference baseline resulting after merging samples displaying null/very little genetic differentiation: Principal Component Analysis (PCA) biplot of the subsequent 12 pooled samples (a) and heatmap of pairwise F_ST_ and associated dendrogram (b). Spawning time represents the major driver of the differentiation as displayed in the PCA first axis and in the main dichotomic division of the dendrogram. F_ST_ and corresponding P-values can be found in **Table S3**.

| a)  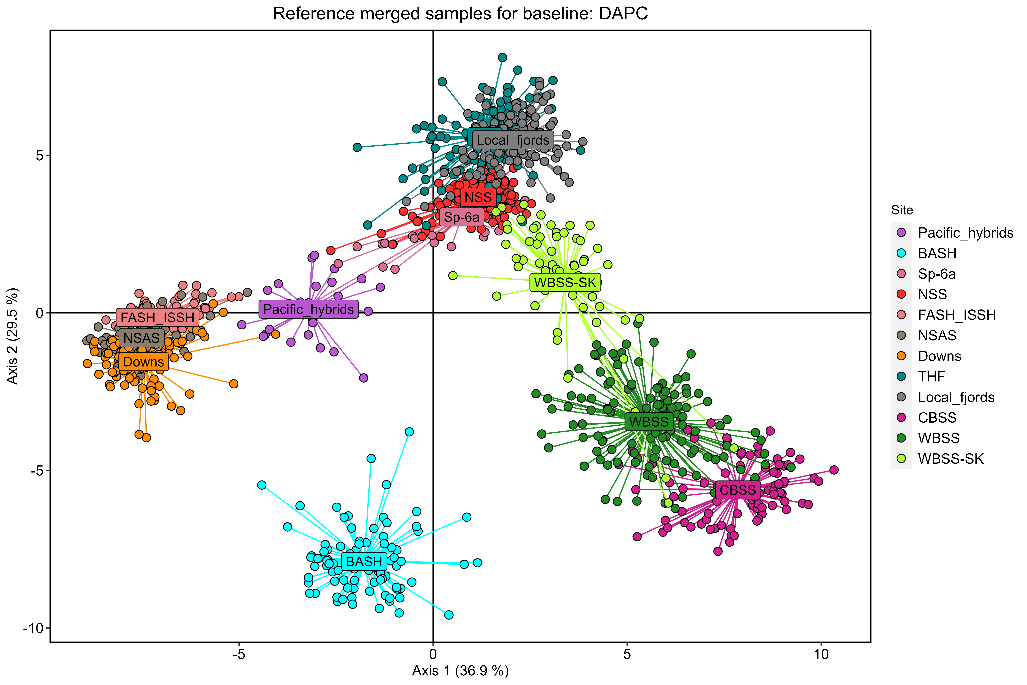 |
| --- |
| b)  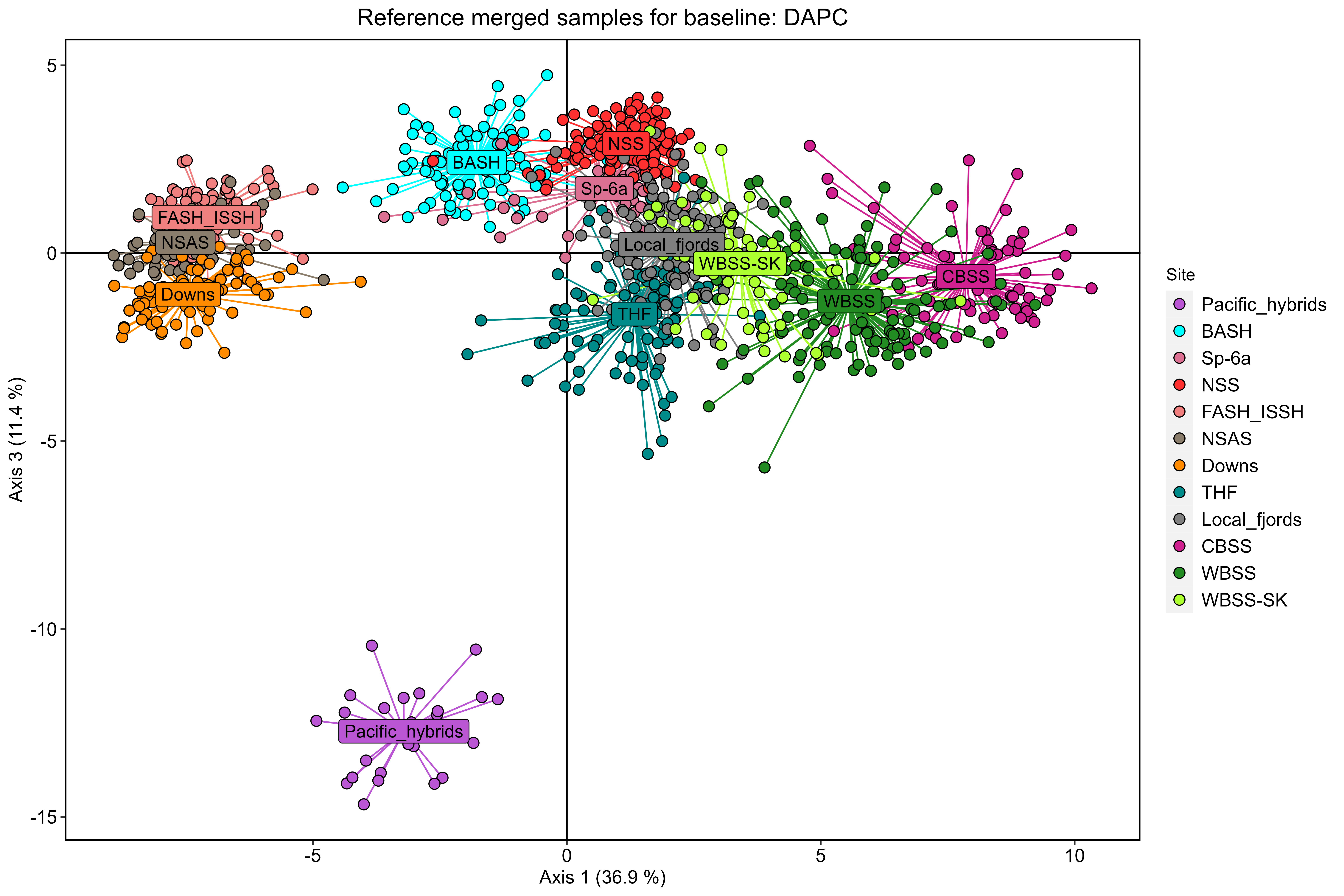 |
| c)  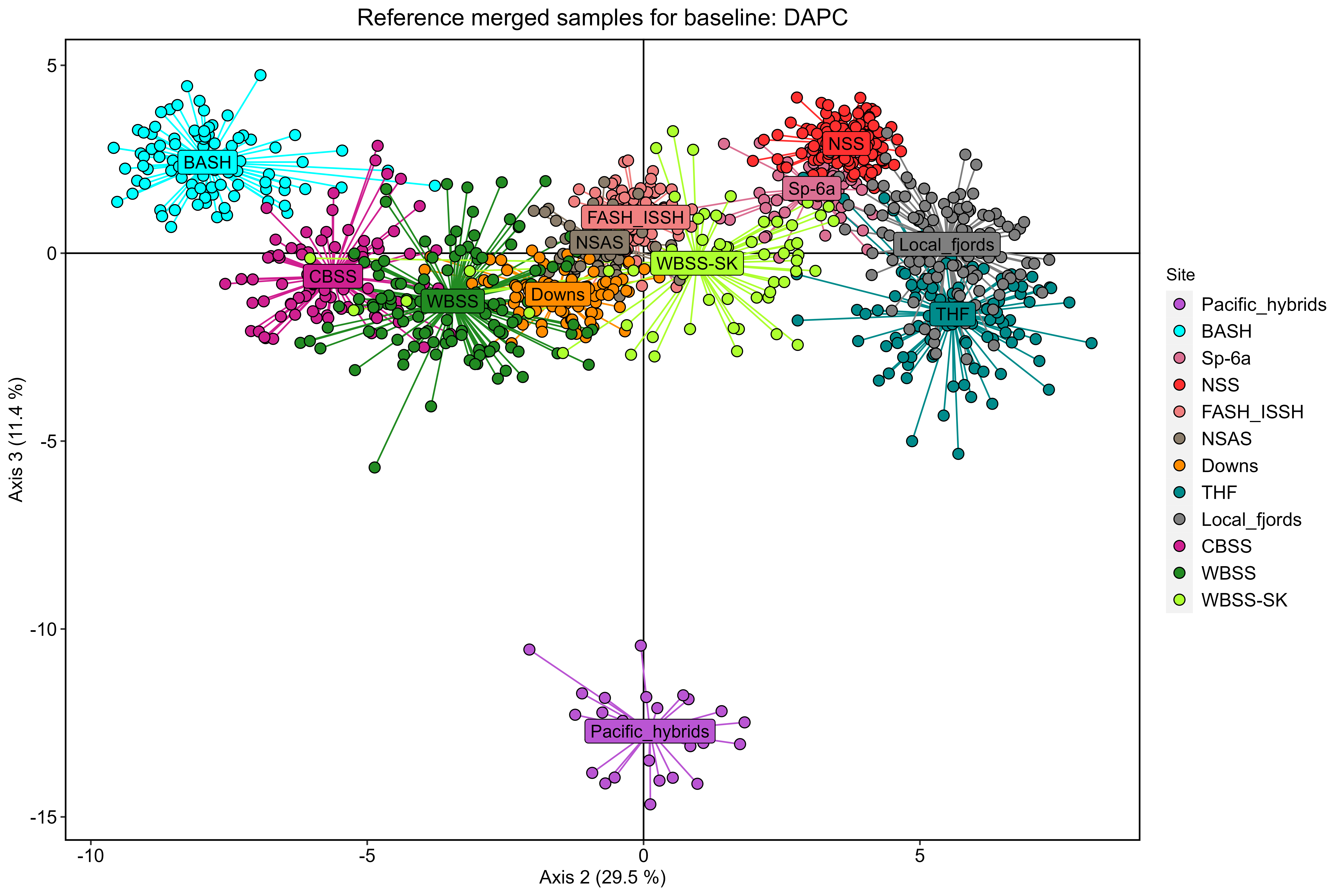 |

**Figure S4**. Reference baseline after sample merging: Discriminant Analysis of Principal Components (DAPC) of the 12 merged samples built after retaining 20 principal components and 3 discriminant functions.

| a)  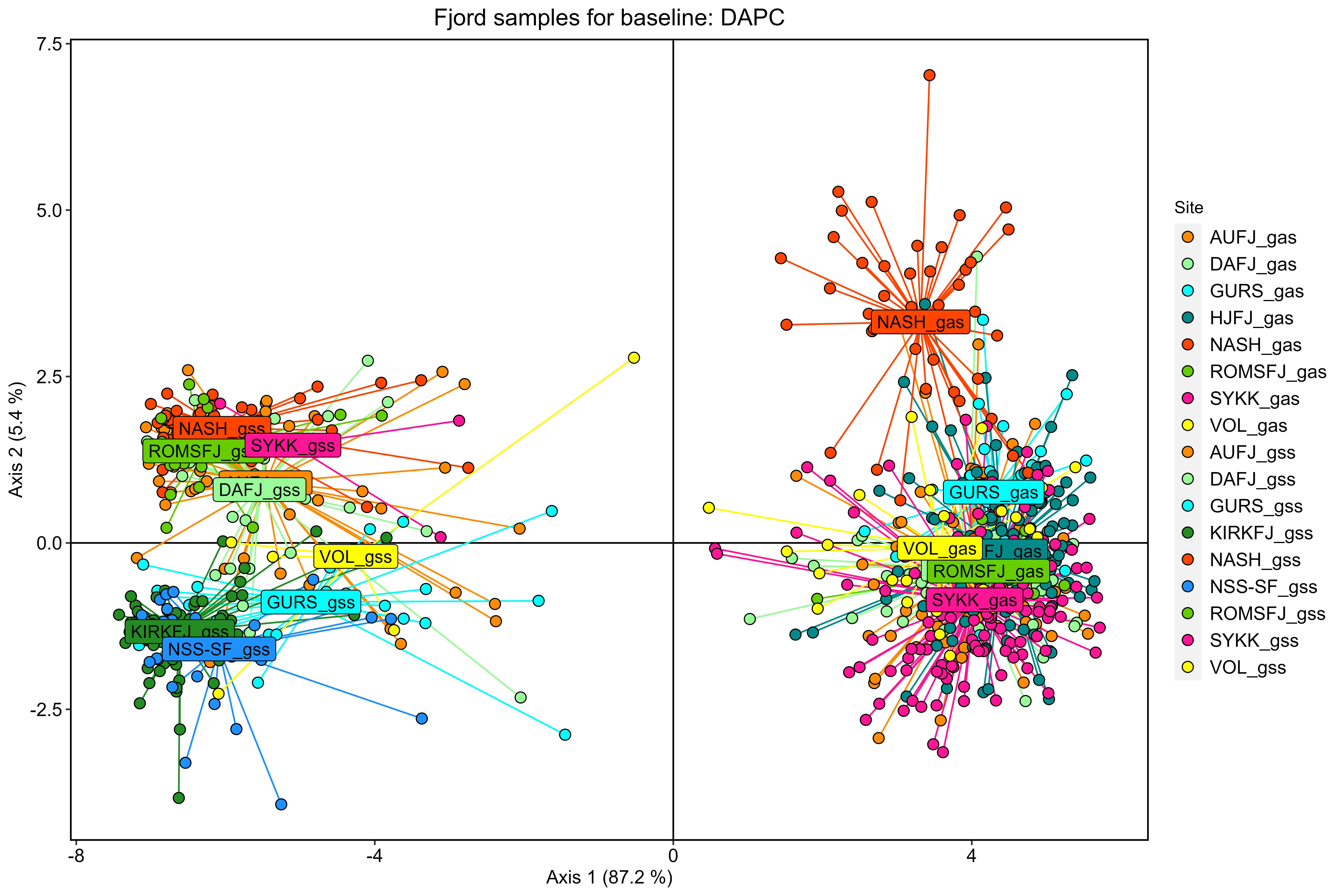 |
| --- |
| b)  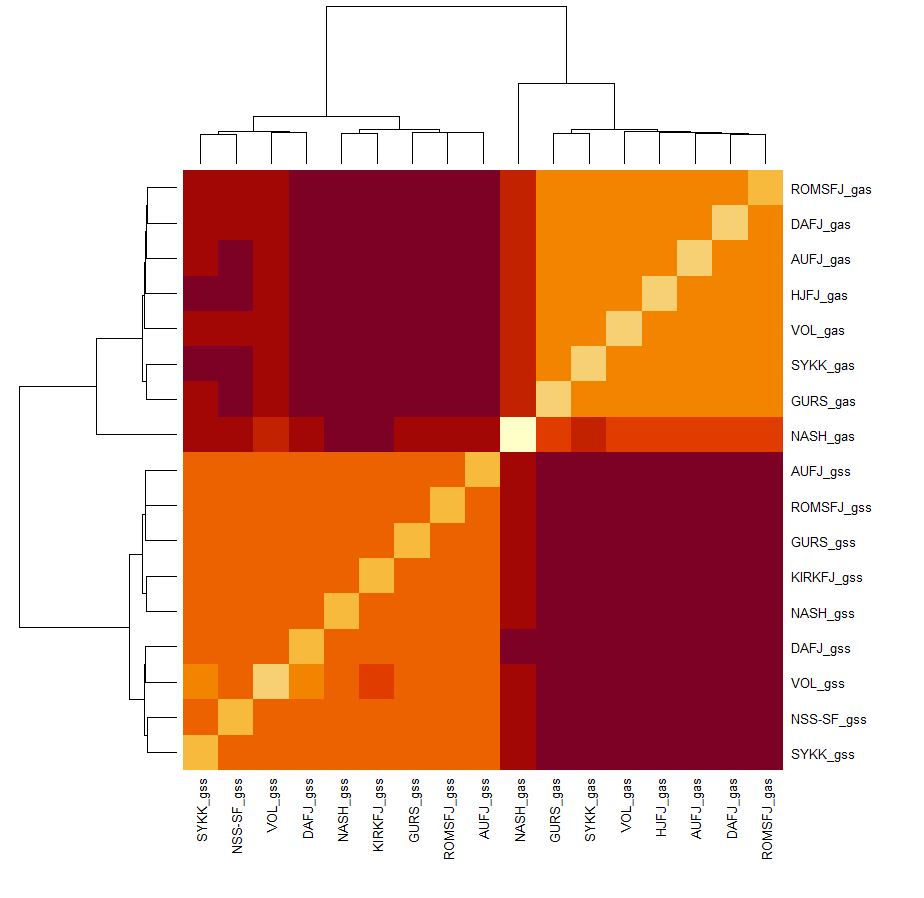 |

**Figure S5**. Potentially new baseline: Principal Component Analysis (PCA) biplot of the fish collected in the Norwegian fjords (a) and heatmap of pairwise *F*_ST_ with associated dendrogram (b). Spawning time represents the major driver of the differentiation as displayed in the first PCA axis as in the main dichotomic division of the dendrogram. *F*_ST_ and corresponding P-values can be found in **Table S4**.

| a)  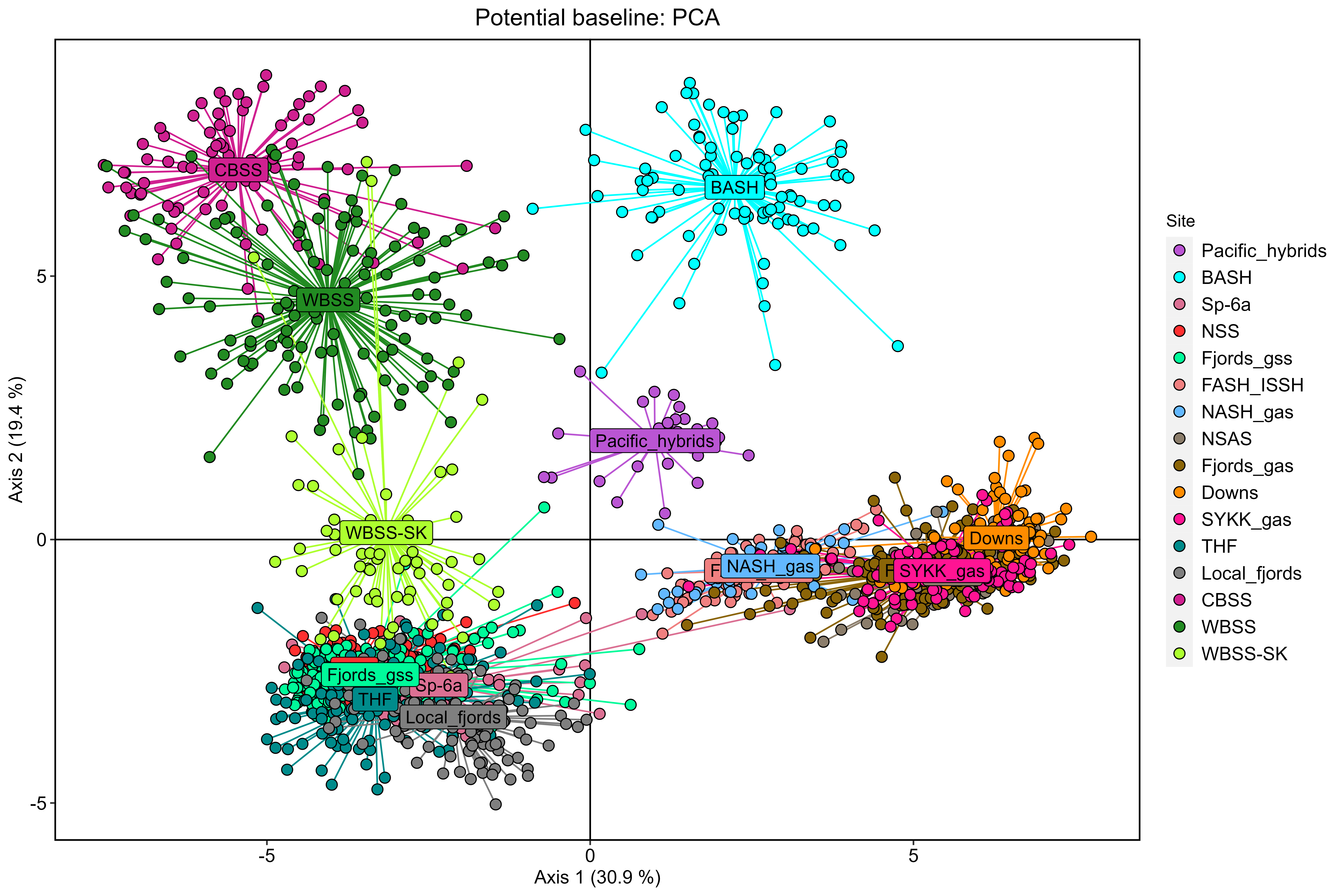 |
| --- |
| b)  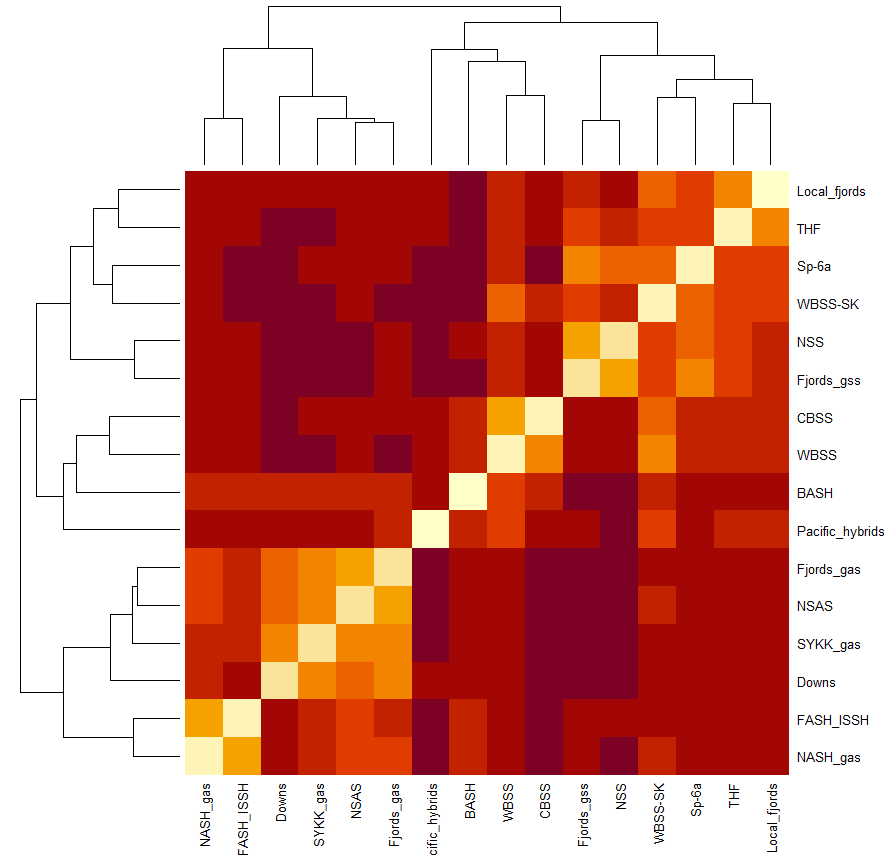 |

**Figure S6**. Joint reference and potentially new baseline: Principal Component Analysis (PCA) biplot (a) and heatmap of pairwise *F*_ST_ and associated dendrogram (b). *F*_ST_ and corresponding P-values can be found in **Table S5.**


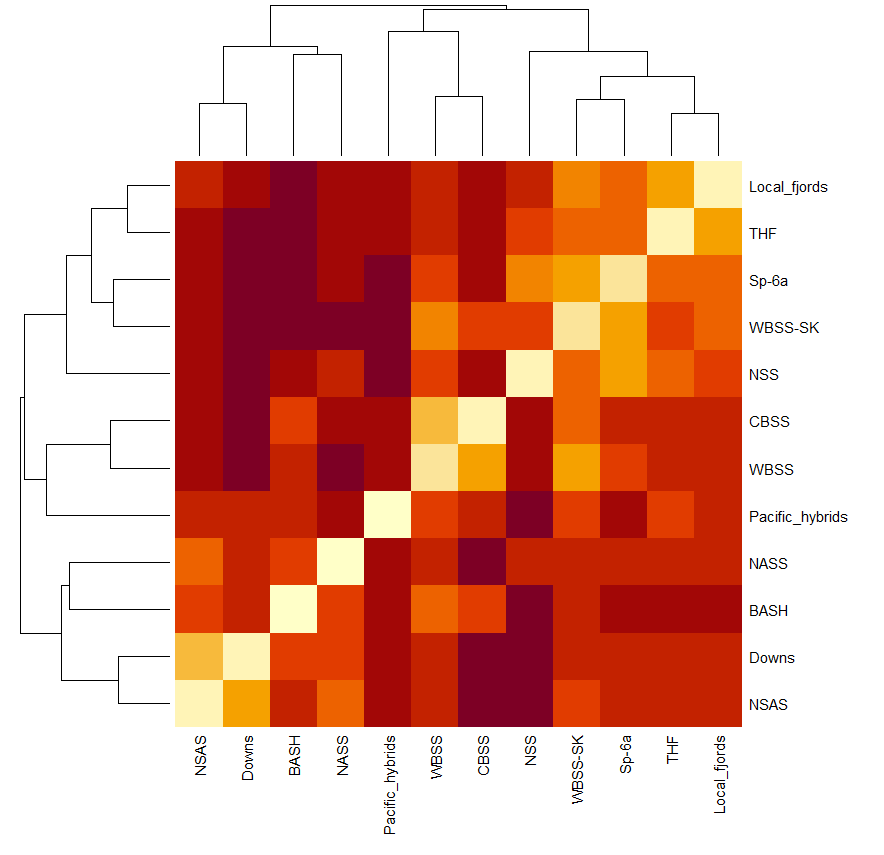


**Figure S7**. Final baseline: Heatmap of pairwise *F*_ST_ and associated dendrogram. *F*_ST_ and corresponding P-values can be found in **Table S6.**

| \| a)  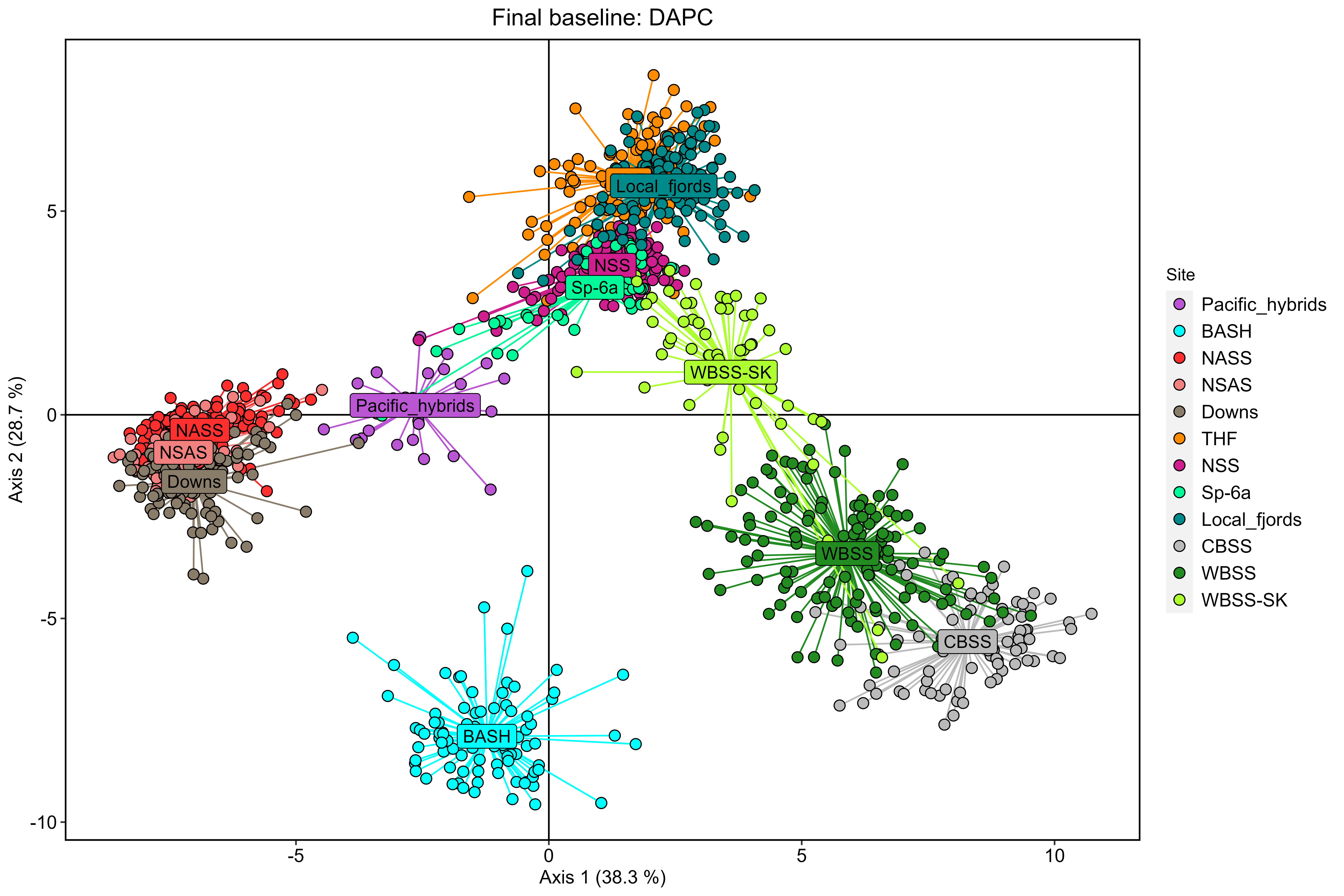 \| \| --- \| \| b)  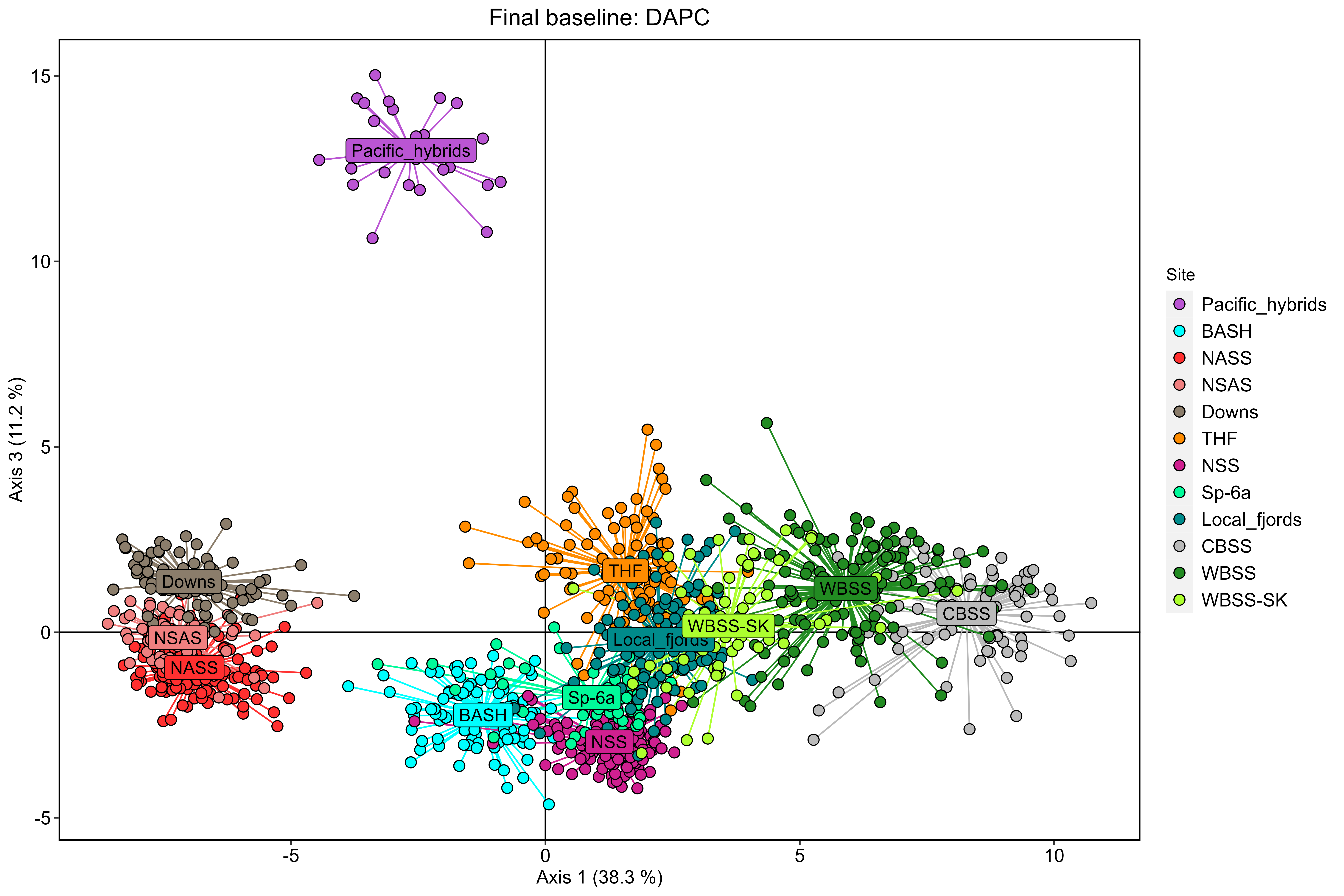 \| \| c)  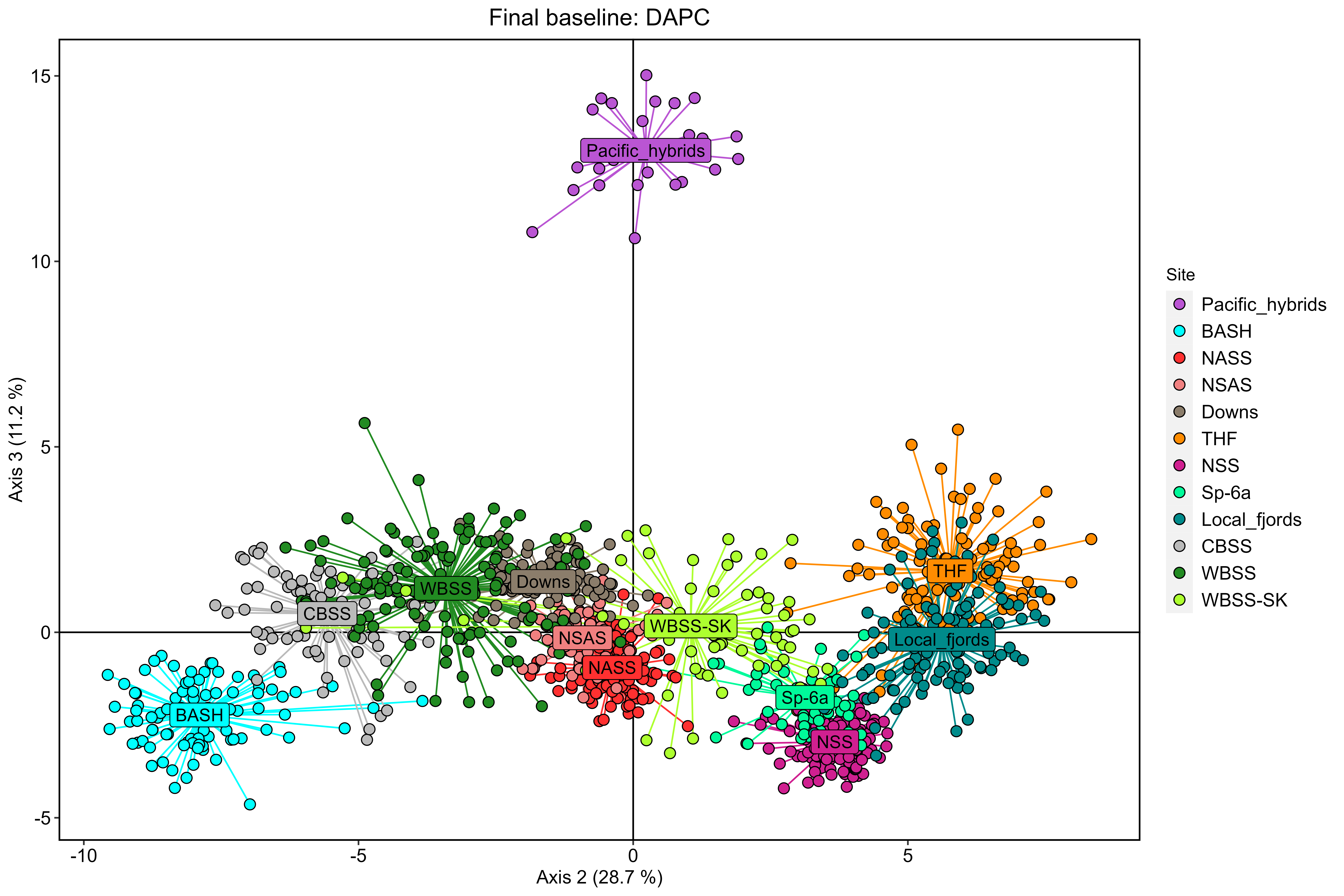 \| |
| --- | --- | --- | --- |
|  |
|  |

**Figure S8**. Final baseline: Discriminant Analysis of Principal Components (DAPC) built after retaining 25 principal components and 3 discriminant functions.

| a)  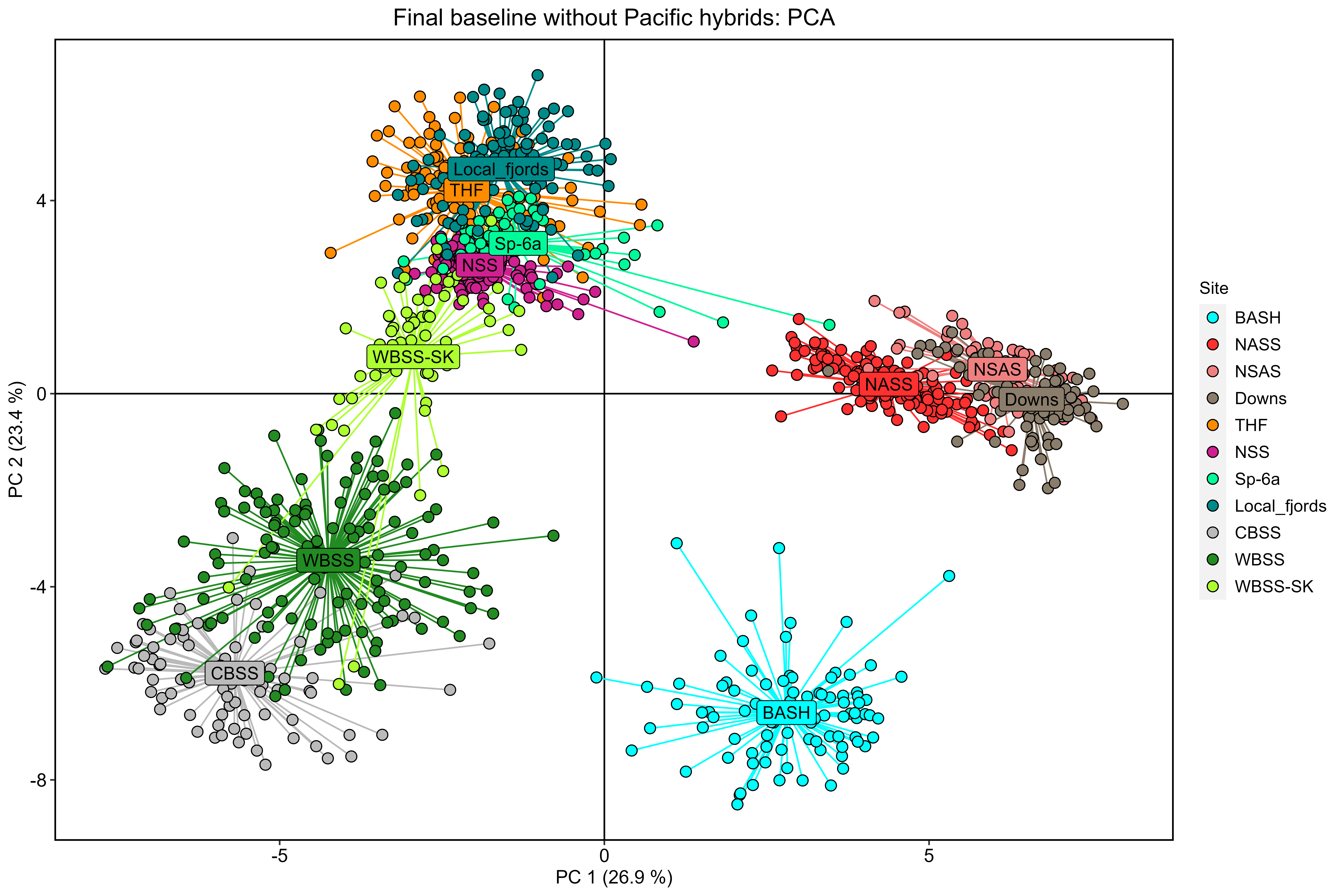 |
| --- |
| b)  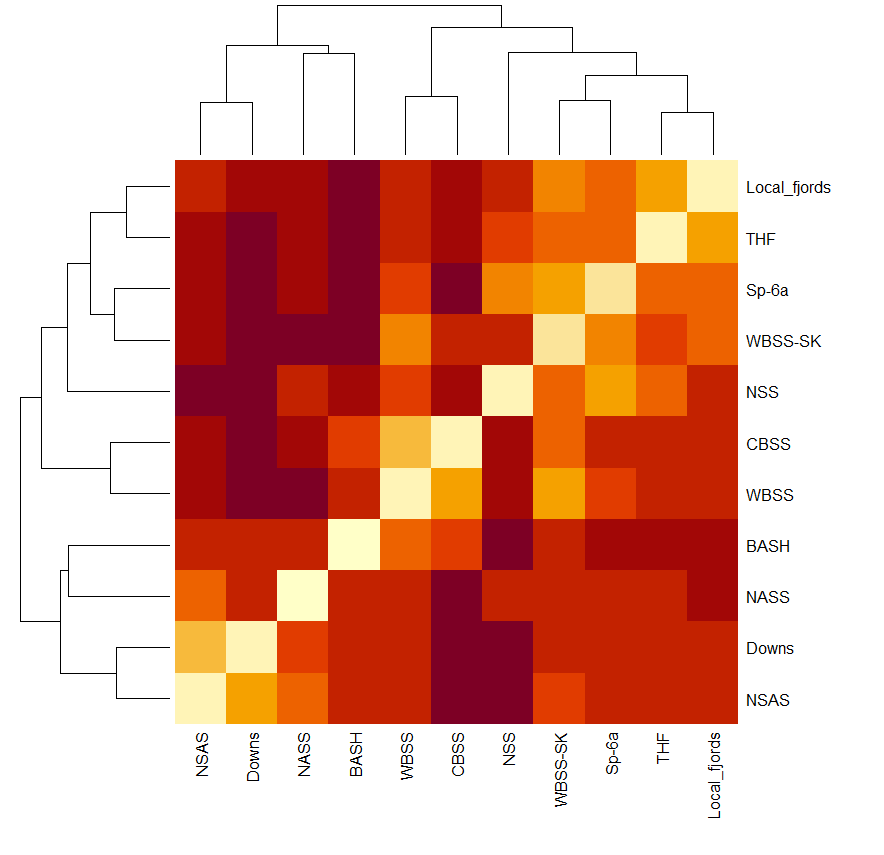 |

**Figure S9**. Final baseline without Pacific hybrids: Principal Component Analysis (PCA) (a) and heatmap of pairwise *F*_ST_ and associated dendrogram.

| a)  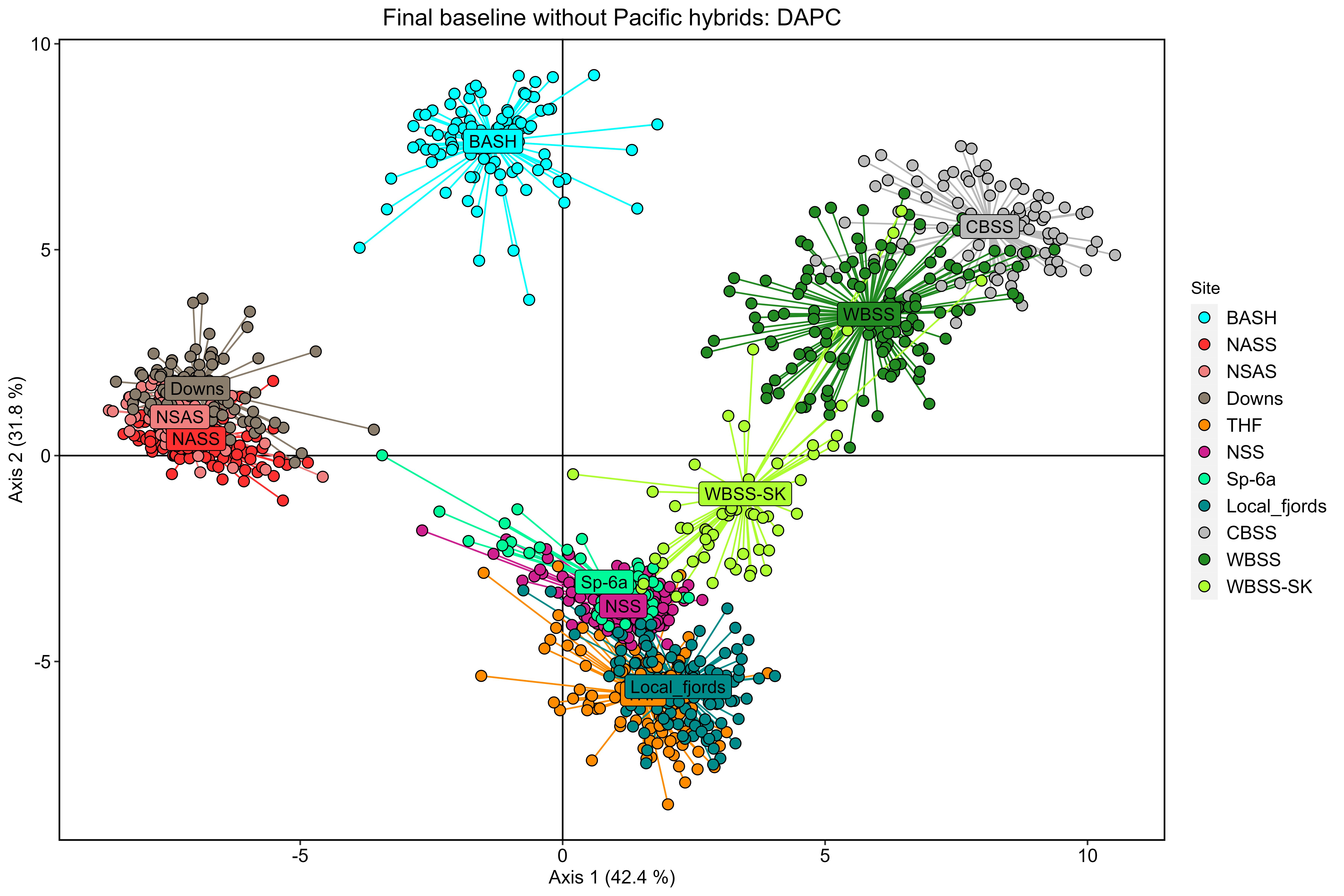 |
| --- |
| b)  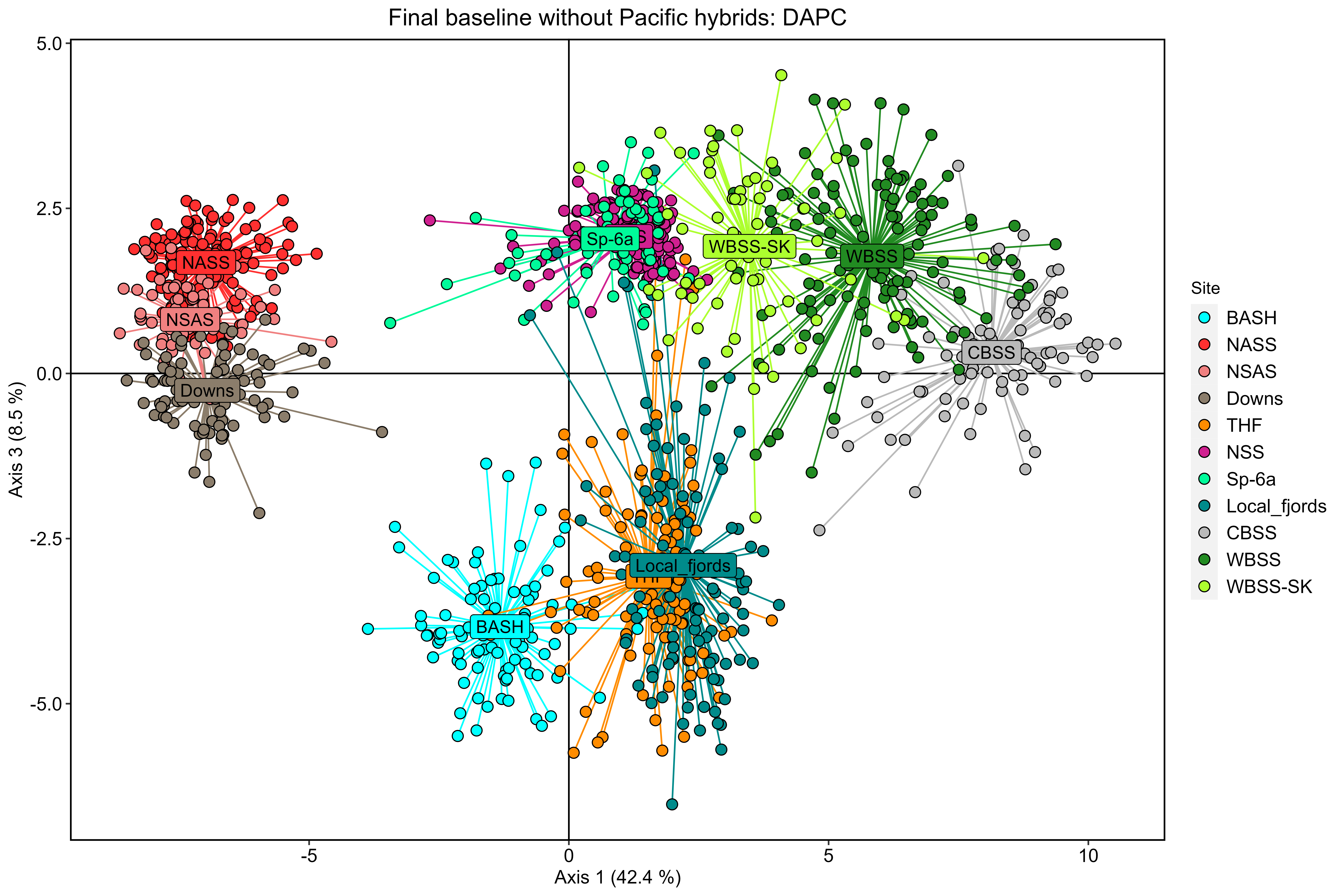 |
| c)  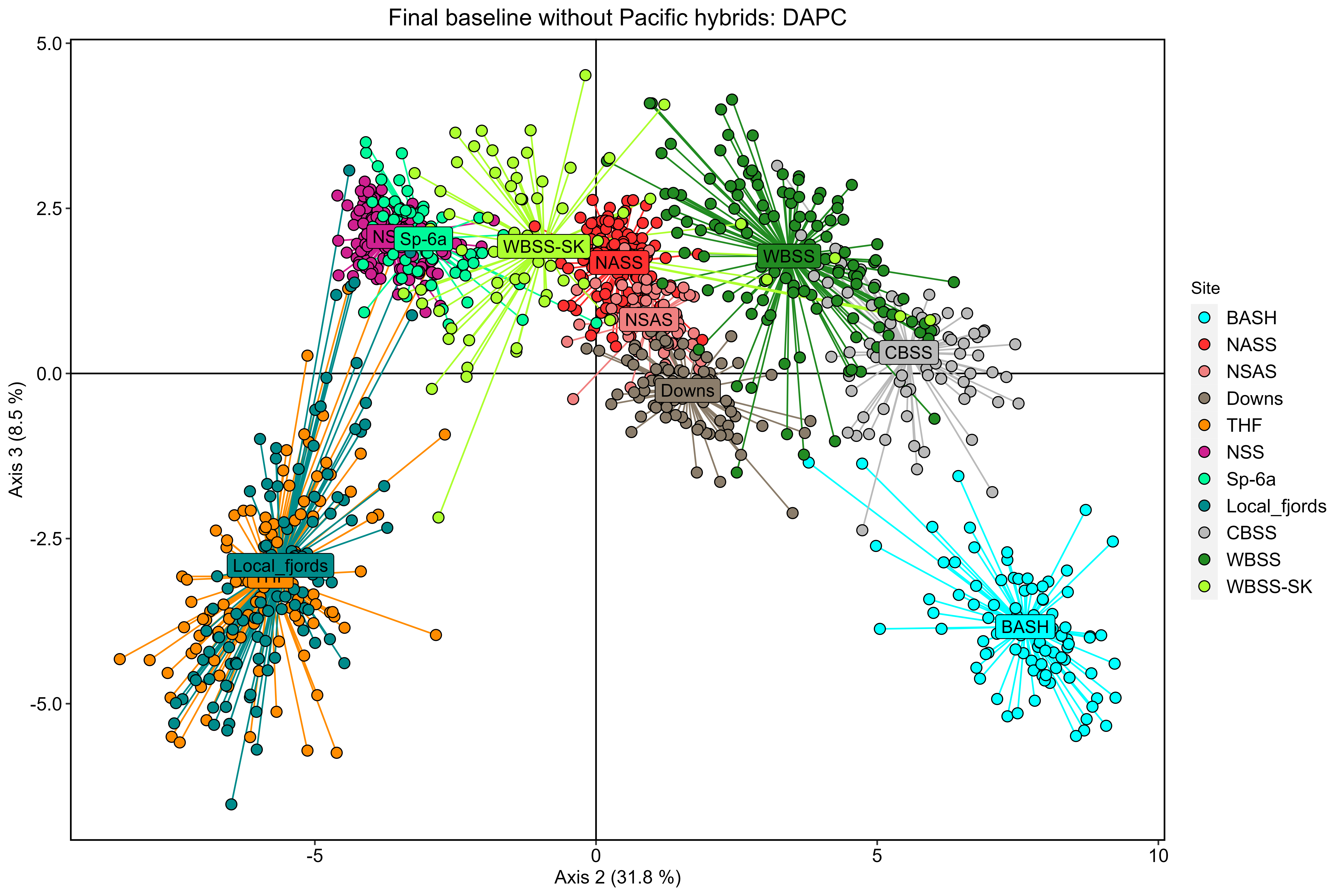 |

**Figure S10**. Final baseline without Pacific hybrids: Discriminant Analysis of Principal Components (DAPC) built after retaining 30 principal components and 3 discriminant functions.

**
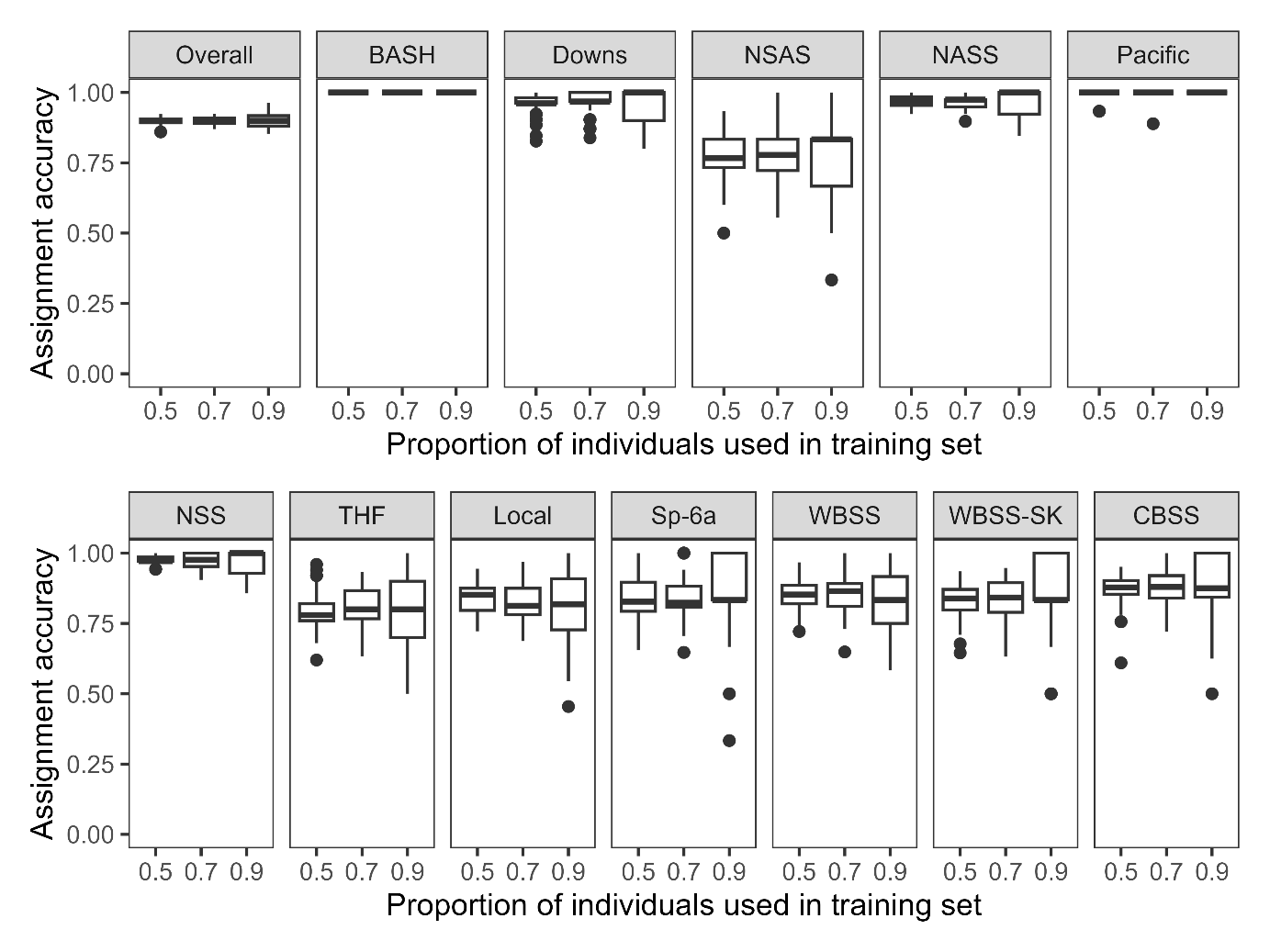
**

**Figure S11.** Self-assignment accuracy of the final baseline estimated with Monte-Carlo cross-validation in *assignPOP*. From each group of the baseline populations 50%, 70%, or 90% were kept in the training dataset.

**
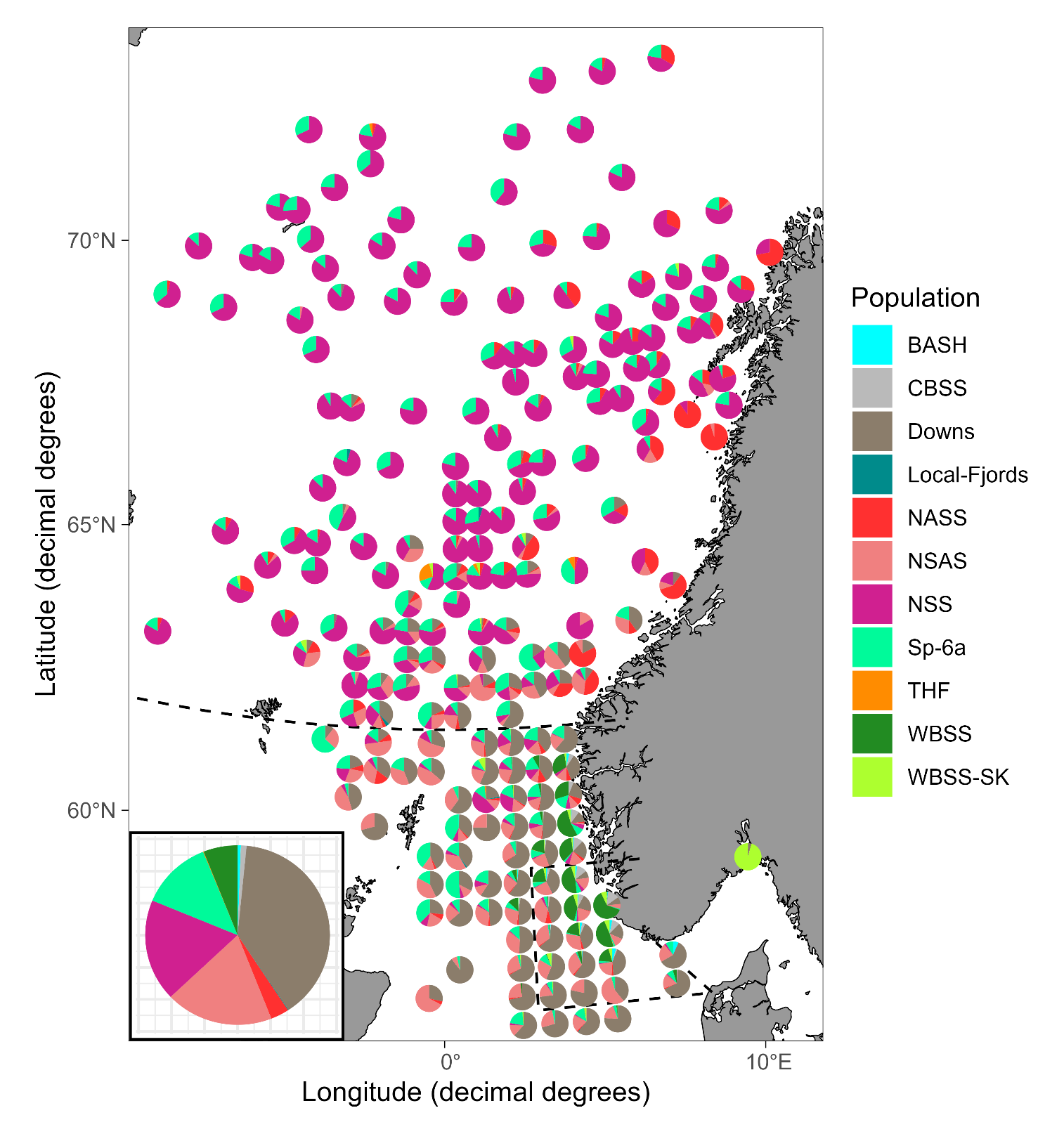
**

**Figure S12.** Assignment results based on *assignPOP* for all mixed-population samples combined for all years and ICES rectangles (1° longitude X 0.5° latitude). Note that the size of the pie charts does not reflect neither catch size nor sample size. Pie chart in the left corner represents the overall distribution of all samples combined. Population abbreviations are explained in the legend of Fig. 3.

**
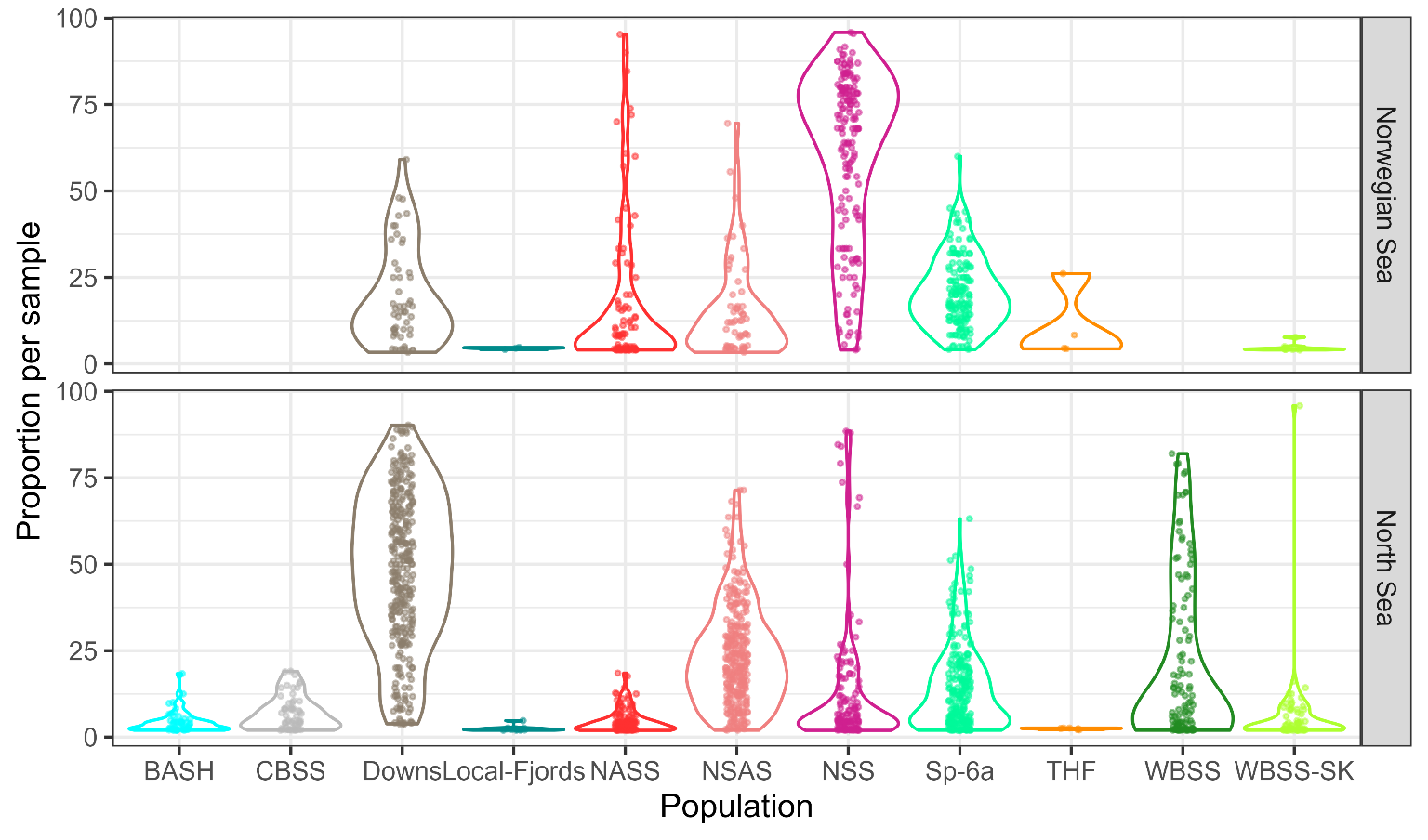
**

**Figure S13.** Violin plot showing the distribution of population proportions for all individual mixed-population samples (N = 457, indicated by points) in the Norwegian Sea (north of 62°N) and the North Sea (south of 62°N). Assignment results based on *assignPOP* for all mixed-population samples. Note that the absence (null observation) per populations is not included. Population abbreviations are explained in the legend of Fig. 3.

**
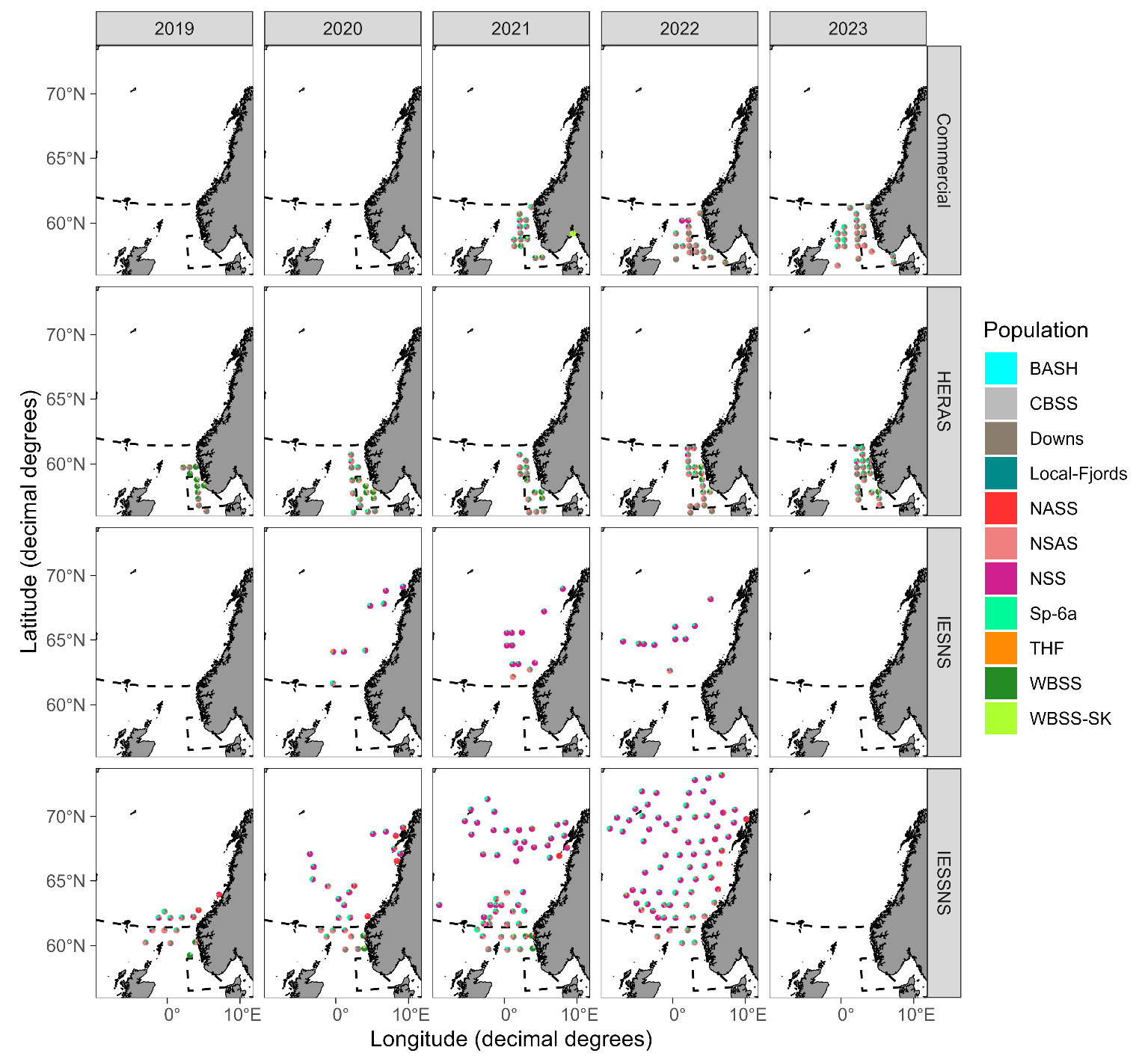
**

**Figure S14.** Assignment results based on *assignPOP* for all mixed-population samples split per year, sampling type (commercial and survey data), and ICES rectangles (1° longitude X 0.5° latitude). Note that the size of the pie charts does not reflect neither catch size nor sample size. Pie chart in the left corner represents the overall distribution of all samples combined. Population abbreviations are explained in the legend of Fig. 3.

**
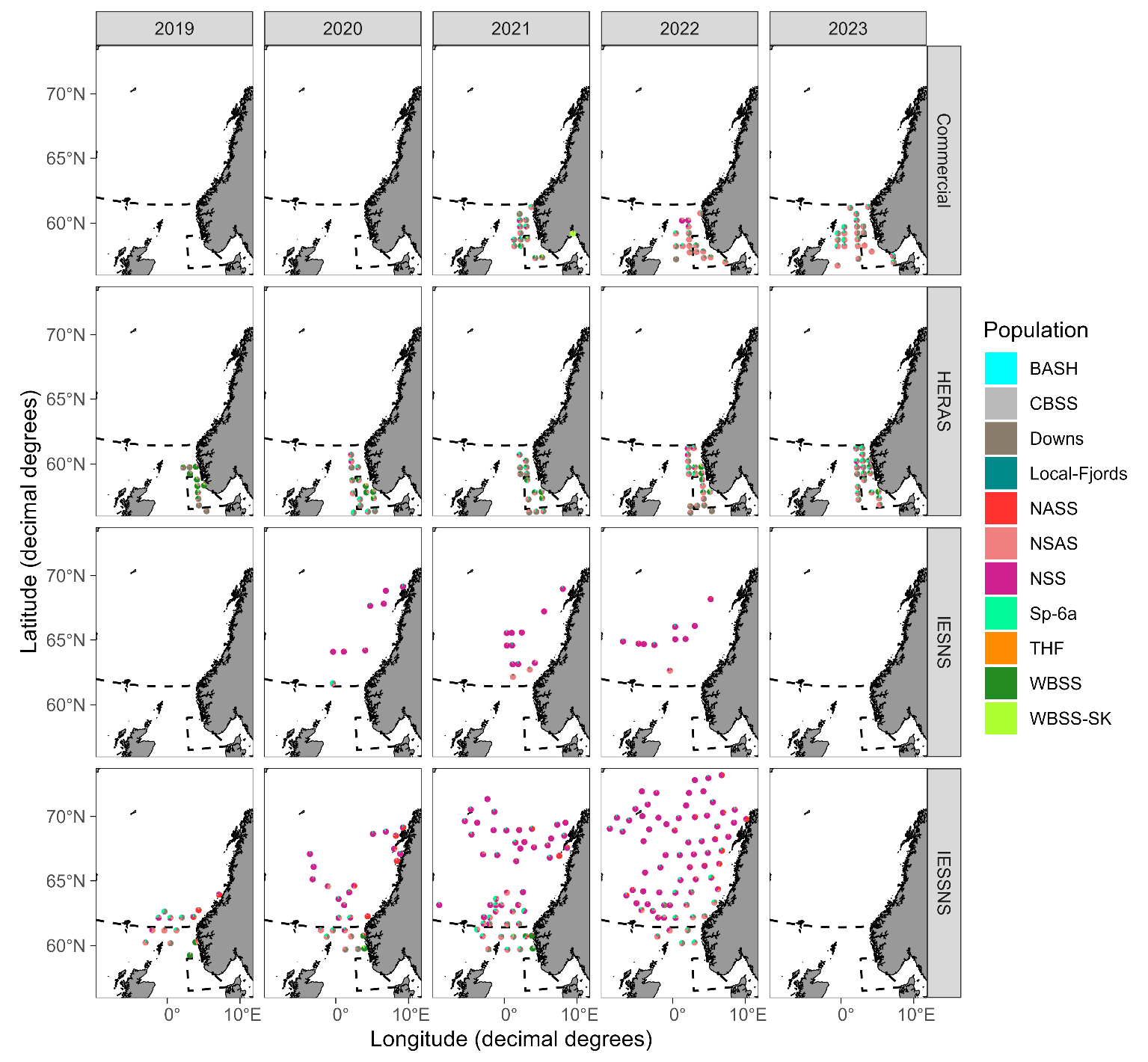
**

**Figure S15.** Assignment results based on *rubias* for all mixed-population samples split per year, sampling type (commercial and survey data), and ICES rectangles (1° longitude X 0.5° latitude). Note that the size of the pie charts does not reflect neither catch size nor sample size. Pie chart in the left corner represents the overall distribution of all samples combined. Population abbreviations are explained in the legend of Fig. 3.
